# A novel high-dimensional model for identifying regional DNA methylation QTLs

**DOI:** 10.1093/biostatistics/kxaf032

**Published:** 2025-10-26

**Authors:** Kaiqiong Zhao, Archer Y Yang, Karim Oualkacha, Yixiao Zeng, Kathleen Klein, Marie Hudson, Inés Colmegna, Sasha Bernatsky, Celia M T Greenwood

**Affiliations:** Department of Mathematics and Statistics, York University, 4700 Keele Street, Toronto, ON, M3J 1P3, Canada; Department of Mathematics and Statistics, McGill University, 805 Sherbrooke Street West, Montréal, Québec, H3A 2K6, Canada; Mila-Québec AI Institute, 6666 Saint-Urbain Street, Montréal, QC, H2S 3H1, Canada; Département de Mathématiques, Université du Québec à Montréal, 201 Avenue du Président-Kennedy, Montréal, QC, H2X 3Y7, Canada; Lady Davis Institute for Medical Research, Jewish General Hospital, 3755 Chemin de la Côte-Sainte-Catherine, Montréal, QC, H3T 1E2, Canada; Quantitative Life Science, McGill University, 845 Sherbrooke Street West, Montréal, QC, H3A 0G4, Canada; Lady Davis Institute for Medical Research, Jewish General Hospital, 3755 Chemin de la Côte-Sainte-Catherine, Montréal, QC, H3T 1E2, Canada; Lady Davis Institute for Medical Research, Jewish General Hospital, 3755 Chemin de la Côte-Sainte-Catherine, Montréal, QC, H3T 1E2, Canada; Department of Medicine, McGill University, 3655 Promenade Sir William Osler, Montréal, QC, H3G 1Y6, Canada; Department of Medicine, McGill University, 3655 Promenade Sir William Osler, Montréal, QC, H3G 1Y6, Canada; The Research Institute of the McGill University Health Centre, 2155 Guy Street, Montréal, QC, H3H 2R9, Canada; Department of Medicine, McGill University, 3655 Promenade Sir William Osler, Montréal, QC, H3G 1Y6, Canada; The Research Institute of the McGill University Health Centre, 2155 Guy Street, Montréal, QC, H3H 2R9, Canada; Lady Davis Institute for Medical Research, Jewish General Hospital, 3755 Chemin de la Côte-Sainte-Catherine, Montréal, QC, H3T 1E2, Canada; Quantitative Life Science, McGill University, 845 Sherbrooke Street West, Montréal, QC, H3A 0G4, Canada; Department of Epidemiology, Biostatistics and Occupational Health, McGill University, 2001 McGill College, Montréal, QC, H3A 1G1, Canada; Department of Human Genetics, McGill University, 3666 McTavish Street, Montréal, QC, H3A 1Y2, Canada; Gerald Bronfman Department of Oncology, McGill University, 5100 de Maisonneuve Boulevard West, Montréal, QC, H4A 3T2, Canada

**Keywords:** methylation QTLs, varying coefficient model, variable selection, smoothness control, proximal gradient descent

## Abstract

Varying coefficient models offer the flexibility to learn the dynamic changes of regression coefficients. Despite their good interpretability and diverse applications, in high-dimensional settings, existing estimation methods for such models have important limitations. For example, we routinely encounter the need for variable selection when faced with a large collection of covariates with nonlinear/varying effects on outcomes, and no ideal solutions exist. One illustration of this situation could be identifying a subset of genetic variants with local influence on methylation levels in a regulatory region. To address this problem, we propose a composite sparse penalty that encourages both sparsity and smoothness for the varying coefficients. We present an efficient proximal gradient descent algorithm that scales to high-dimensional predictor spaces, providing sparse solutions for the varying coefficients. A comprehensive simulation study has been conducted to evaluate the performance of our approach in terms of estimation, prediction and selection accuracy. We show that the inclusion of smoothness control yields much better results over sparsity-only approaches. An adaptive version of the penalty offers additional performance gains. We further demonstrate the utility of our method in identifying regional mQTLs from asymptomatic samples in the CARTaGENE cohort. The methodology is implemented in the R package sparseSOMNiBUS, available on GitHub.

## 1. INTRODUCTION

DNA methylation is a key epigenetic mechanism that regulates gene expression, drives tissue differentiation, and contributes to disease susceptibility. It primarily occurs at cytosine-guanine dinucleotides (CpG sites), and its variation is often influenced by genetic factors (Gaunt et al. 2016; Hannon et al. 2018). Single-nucleotide polymorphisms (SNPs) that modulate methylation levels are known as methylation quantitative trait loci (mQTLs). Mapping mQTLs is critical for understanding the genetic regulation of the epigenome and mitigating confounding in epigenome-wide association studies (Hannon et al. 2016; Van Dongen et al. 2016; Taylor et al. 2019).

### 1.1. Motivation for regional mQTL mapping with smooth genetic effects

Traditional mQTL mapping typically tests one SNP–one CpG association at a time, treating CpG sites independently (Zhou and Stephens 2014; Fan et al. 2019). However, these approaches overlook the spatial correlation in DNA methylation observed across neighboring sites. Bisulfite sequencing (BS), which offers base-pair resolution, enables detection of these local dependencies and has shown promising results in mQTL studies (Schmitz et al. 2013; Cheung et al. 2017).

**Fig. 1.**
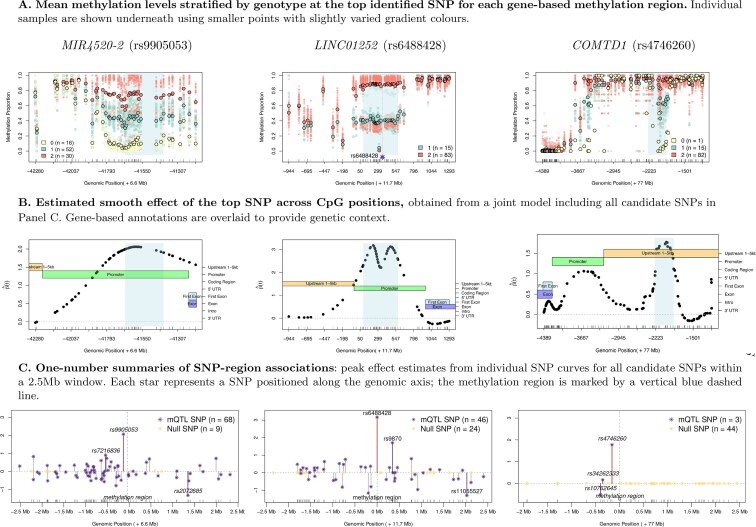
Regional mQTL patterns across three gene-based methylation regions. Each region is defined as the first exon and 2 kb upstream of the corresponding protein-coding gene. Shown are three representative regions—*MIR4520-2*, *LINC01252*, and *COMTD1*—ranked 7th, 1st, and 11th, respectively, out of 12,283 gene-defined regions (see Table 2). The rows display A) methylation proportions by genotype, B) estimated smooth SNP effects, and C) peak effect magnitudes across all candidate SNPs within a ± 2.5 Mb window. Together, these patterns illustrate the need to model both smooth and sparse genetic effects in regional mQTL analysis. Additional examples are shown in Fig. S11.

Our motivating dataset includes DNA methylation measurements from Targeted Custom Capture Bisulfite Sequencing, designed to capture methylation levels in biologically relevant regions. It profiles approximately 5 million CpG sites in whole blood samples from 98 individuals. As shown in Fig. 1A and Fig. S11A, methylation levels exhibit strong spatial correlation within regions, and SNP effects tend to be regionally structured—affecting clusters of neighboring CpGs rather than isolated sites. Although abrupt shifts can occur at individual CpG sites, the magnitude of genetic effects more often varies smoothly across nearby positions, particularly within regulatory domains. These empirical patterns motivate the use of *smooth effect modeling* for regional mQTL analysis. Assuming smooth genetic effects allows for information sharing across CpGs, helping to address missing values and low coverage—challenges frequently encountered in bisulfite sequencing data. This assumption is supported by prior studies showing strong spatial correlation in methylation levels among CpGs located within 1 kilobase pair (kbp) (Eckhardt et al. 2006), and consistent co-methylation across species and tissues (Affinito et al. 2020).

Therefore, this article aims to identify regional mQTLs that drive coordinated, smoothly varying methylation changes across subsets of adjacent CpGs.

To capture coordinated methylation changes across neighboring CpGs, we model the *joint effects of multiple candidate SNPs*. A multivariable framework—assessing each SNP’s contribution while adjusting for others—can reveal variants that may have weak marginal associations but exert meaningful effects in combination, particularly when linked SNPs jointly influence methylation patterns. To this end, we adopt a varying coefficient (VC) regression framework to simultaneously estimate multiple SNP effects that vary smoothly along genomic positions.

### 1.2. Statistical challenges: unified control of sparsity and smoothness

While traditional VC models, including our earlier work (Zhao et al. 2021, 2024) and that of Wood (2011), offer flexible tools for modeling smooth covariate effects, they are not designed for high-dimensional predictor spaces. In regional mQTL mapping, hundreds or thousands of candidate SNPs often lie within or near a regulatory region, while sample sizes remain small due to sequencing costs and sample availability (as seen in our motivating dataset; see Table 2 and Table S20). Low-dimensional methods face significant challenges when estimating the varying coefficients in such a high-dimensional setting (Fan et al. 2014; Chouldechova and Hastie 2015). Moreover, only a small subset of the candidate SNPs is expected to independently influence the methylation patterns. Traditional VC models using quadratic smoothness penalties cannot provide sparse solutions for the varying coefficients, and are thus unsuitable for regional mQTL mapping. These limitations motivate the need to extending VC models to jointly address sparsity and smoothness, enabling scalable estimation and interpretable results.

**Sparsity control** in VC models becomes more challenging when covariate effects vary with respect to structured or multi-dimensional modifiers, motivating methods that jointly select both main predictors and their modifiers. The pliable LASSO (Tibshirani and Friedman 2020) addresses this by modeling varying coefficients as *linear* functions of modifiers for simultaneous selection of both predictors and their interactions. Along these lines, the structural varying-coefficient regression (svReg) method (Kim et al. 2021) introduces hierarchical group penalties to accommodate structured dependencies within both predictors and modifiers.

However, in the context of bisulfite sequencing data, the modifier of interest—genomic position—is fixed and linearly ordered along the DNA sequence. Thus, genomic position naturally serves as a one-dimensional modifier (Fig. 1). In our setting, high dimensionality arises from the large number of main predictors (SNPs), each with potentially *nonlinear* effects along this 1D modifier. To enable variable selection in such VC models, many sparse penalized regression methods have been developed, differing mainly in their choices of penalty functions. Major classes include LASSO (Lin and Zhang 2006), group LASSO (Meier et al. 2009; Wang and Xia 2009; Ravikumar et al. 2009; Huang et al. 2010; Wei et al. 2011; Gertheiss et al. 2013; Barber et al. 2017), SCAD (Wang et al. 2007, 2008; Noh and Park 2010) and L0-penalization (Xue and Qu 2012).

Notably, beyond sparsity, VC models require **smoothness regularization** to control the complexity of nonlinear coefficient functions—particularly in our setting, where SNP effects vary along genomic position and are estimated using rich spline expansions. Classical approaches address smoothness either by limiting the number of basis functions (Ravikumar et al. 2009; Huang et al. 2010; Xue and Qu 2012) or by imposing quadratic penalties, as in penalized or smoothing splines (Wood 2011). The former relies on ad hoc truncation and offers limited flexibility, as the optimal basis dimension is difficult to determine and often context-dependent. The latter starts with a comparatively large number of basis expansion and then impose smoothness penalties, enabling finer control without relying on rigid tuning of basis dimension. Recent work has also explored adaptive smoothness strategies in nonparametric regression (Park et al. 2023; Wang et al. 2024), but these methods typically address smoothness alone.

Despite efforts to combine sparsity and smoothness, existing methods often address them separately. For example, Wang et al. (2008) model smooth coefficient functions using spline basis expansions and apply a group SCAD penalty for sparsity, with smoothness controlled by selecting the basis dimension from a small set of discrete values. Wang and Xia (2009) propose a two-step procedure: they first select a smoothing bandwidth assuming no sparsity constraints, and then tune the sparsity shrinkage parameter under the selected bandwidth. These approaches rely on separate tuning steps or discrete modeling choices that are difficult to optimize and often sensitive to their exact specification. Moreover, this limits flexibility in capturing heterogeneous signal patterns and undermines stability in high-dimensional settings, where stepwise tuning often leads to inconsistent selection. Therefore, we argue that a **unified** penalization strategy that jointly controls sparsity and smoothness through continuous tuning is preferable.

### 1.3. Overview of the proposed method

To address these challenges, we propose a *novel sparse high-dimensional varying coefficient model* for regional mQTL mapping. Our method integrates sparsity and smoothness within a unified framework, enabling continuous, data-adaptive control over both. Leveraging spline-based estimation with adaptive penalties, the model flexibly captures a range of effect shapes—including smooth trends and moderate local deviations—without oversmoothing or imposing restrictive functional forms. As shown in Fig. 1B and Fig. S11B, the method recovers smoothly varying SNP effects while remaining sensitive to strong localized signal fluctuations. Simultaneously, it shrinks less important effects to zero (Fig. 1C, Figs S11C, S12 to S14), enabling variable selection in high-dimensional settings.

Specifically, we formulate the model using a binomial likelihood that accounts for read depth, with SNP effects represented as smooth functional coefficients varying along genomic position. To jointly enforce sparsity and smoothness, we introduce a composite penalty function inspired by the sparsity-smoothness framework of Meier et al. (2009), with two tuning parameters that separately control model complexity and functional smoothness.

For estimation, we develop an efficient proximal gradient descent algorithm, noted for its scalability with high-dimensional predictors and its capacity to accommodate non-differentiable penalty functions. A backtracking line search is implemented to adaptively determine step sizes and ensure stable convergence. Automatic selection of tuning parameters is integrated via cross-validation. We further extend the method to allow covariate-specific adaptive penalization, allowing different levels of regularization across SNPs. The full framework is implemented in the open-source R package sparseSOMNiBUS, available on GitHub.

The remainder of the article is organized as follows. Section 2 introduces the proposed model and penalized likelihood. Section 3 details the proximal gradient descent algorithm with backtracking line search. Section 4 extends the method to allow adaptive penalization across covariates. Section 5 presents simulation studies. Section 6 demonstrates the utility of the method in a real-data application involving regional mQTL mapping in the CARTaGENE cohort. We conclude with a discussion in Section 7.

## 2. A HIGH-DIMENSIONAL VARYING COEFFICIENT MODEL FOR mQTL MAPPING

We consider DNA methylation measures over a genomic region from $ N $ independent samples. Let $ m_{i} $ be the number of measured CpG sites for the $ i $-th sample, $ i\,=\,1,2 , \ldots, N $. Let $ t_{ij} $ be the genomic position (in base pairs) for the $ i $-th sample at the $ j $-th CpG site, $ j\,=\,1,2 , \ldots, m_{i} $. Methylation levels at a site are quantified by the number of methylated reads $ Y_{ij} $ and the total number of reads $ X_{ij} $. For each sample, genotype information on $ P $ candidate SNPs is denoted by $ \mathbf{Z}_{i}=\left(Z_{i1},Z_{i2},\ldots, Z_{iP}\right)\in\mathbb{R}^{P} $. We consider high-dimensional varying coefficient models with a binomial outcome $ Y_{ij} $ and $ P $ covariates/SNPs $ \left(Z_{i1},Z_{i2},\ldots, Z_{iP}\right)\in\mathbb{R}^{P} $ connected through


(2.1) \begin{align*}\log\dfrac{\pi_{ij}}{1-\pi_{ij}}=\beta_{0}(t_{ij})+\sum\limits_{p=1}^{P}\beta_{p}(t_{ij})Z_{ip},\end{align*}

where $ \pi_{ij}=\mathbb{E}(Y_{ij})/X_{ij} $ is the individual’s methylation proportion and $ \left\{\beta_{p}(t_{ij})\right\}_{p\,=\,0}^{P}:\mathbb{R}\rightarrow\mathbb{R} $ are smooth univariate functions.

### 2.1. The sparsity-smoothness penalty

Given the vast array of candidate SNPs, only a subset is expected to influence regional methylation patterns. This motivates the need for sparse functional estimators, where $ \widehat{\beta}_{p}(t)=0 $ for specific indices $ p\in\left\{1 , \ldots, P\right\} $. For nonzero coefficients, it is crucial to use a sufficient number of basis functions to capture complex functional patterns, while allowing the level of smoothness—whether reflecting broad trends or localized deviations—to adapt flexibly to the data. To achieve this, we propose a composite penalty function. This approach is inspired by the sparsity-smoothness penalty (SSP) framework proposed by Meier et al. (2009), which is designed for variable selection in high-dimensional additive models. Specifically, we define our penalty function as


(2.2) \begin{align*}\mathcal{L}^{\mathrm{SSP}}(\boldsymbol{\theta})=\lambda\sum\limits_{p=1}^{P}\sqrt{(1-\alpha)J_{1}\left(\beta_{p}(t)\right)+\alpha J_{2}\left(\beta_{p}(t)\right)},\end{align*}

where


\begin{align*} J_{1}(\beta_{p}(t))=\|\beta_{p}(t)\|_{2}^{2}=\int\left(\beta_{p}(t)\right)^{2} dt , \qquad J_{2}(\beta_{p}(t))=M^{2}\int\left(\beta_{p}^{\prime\prime}(t)\right)^{2}dt , \end{align*}

with $ M=\sum_{i\,=\,1}^{N}m_{i} $. Here $ J_{1} $ quantifies the L2-norm of the functional coefficients $ \beta_{p}(t) $, and $ J_{2} $ controls the smoothness of $ \beta_{p}(t) $. The squared root over both $ J_{1} $ and $ J_{2} $ enables the sparsity of $ \beta_{p}(t) $ at the function level.

The amount of penalization in (2.2) is jointly controlled by two tuning parameters, $ \lambda\geq 0 $ and $ 0\leq\alpha < 1 $. The parameter $ \lambda $ governs overall model complexity, while $ \alpha $ adjusts the relative emphasis on smoothness versus function magnitude. When $ \alpha\,=\,0 $, no smoothness constraint would be imposed, and as $ \alpha\rightarrow 1 $, smoother estimates would be favored. The scaling constant $ M^{2} $ ensures $ J_{1}(\beta_{p}(t)) $ and $ J_{2}(\beta_{p}(t)) $ are on comparable scales, thereby facilitating the specification of candidate values for $ \alpha $ during cross-validation.

Note that setting $ \alpha\,=\,1 $ yields a degenerate case: the penalty becomes purely $ J_{2} $ (smoothness only), which does not penalize constant or linear components (since $ \beta_{p}^{\prime\prime}(t)=0 $ for such functions). Consequently, $ \alpha\,=\,1 $ can lead to non-unique, unbounded solutions, and is therefore not permitted in our framework. Also note that the penalty function in (2.2) does not apply to the intercept term. Unlike the SNP effects, the intercept function $ \beta_{0}(t) $ is left unpenalized and is estimated directly from the data without regularization.

### 2.2. Basis representation

For each function coefficient $ \beta_{p}(t_{ij}) $, we use natural cubic spline parameterization with a reasonably large number of basis functions. Without loss of generality, we use the same expansion dimension $ K $ for all the functional coefficients in (2.1), ie


\begin{align*}\beta_{p}(t)=\boldsymbol{\theta}_{p}^{T}\mathbf{B}(t)=\sum\limits_{k=1}^{K}\theta_{p, k}b_{k}(t),\mathrm{for}p=0 , \ldots, P , \end{align*}

where $ \mathbf{B}(t)=(b_{1}(t),\ldots, b_{K}(t))^{T} $ consists of $ K $ natural cubic basis functions $ b_{k}(t):\mathbb{R}\rightarrow\mathbb{R} $ and $ \boldsymbol{\theta}_{p}=(\theta_{p , 1},\ldots , \theta_{p, K})^{T} $ is a vector of coefficients with $ \theta_{p, k} $ being the coefficient for the $ k $-th basis of the $ p $-th covariate. Specific expressions for basis functions $ b_{k}(t) $ used in our implementation can be found in Equation (SA.5) in the Supplementary Material.

Under this basis parametrization, the $ J_{1} $ and $ J_{2} $ in (2.2) can be expressed as


\begin{align*} J_{1}\left(\beta_{p}(t)\right)=\boldsymbol{\theta}_{p}^{T}\boldsymbol{\Omega}^ {(1)}\boldsymbol{\theta}_{p},\qquad J_{2}\left(\beta_{p}(t)\right)=\boldsymbol {\theta}_{p}^{T}\boldsymbol{\Omega}^{(2)}\boldsymbol{\theta}_{p},\end{align*}

where $ \boldsymbol{\Omega}^{(1)} $ and $ \boldsymbol{\Omega}^{(2)} $ are two $ K\times K $ matrices with the $ (k, k^{\prime}) $-th element $ [\boldsymbol{\Omega}^{(1)}]_{k, k^{\prime}}=\int b_{k}(t)b_{k^{\prime}}(t)dt $, and $ [\boldsymbol{\Omega}^{(2)}]{}_{k, k^{\prime}}=M^{2}\int b_{k}^{\prime\prime}(t) b_{k^{\prime}}^{\prime\prime}(t)dt $, respectively, for $ k, k^{\prime}\in\{1 , \ldots, K\} $. Notably, the sparsity-penalty matrix $ \boldsymbol{\Omega}^{(1)} $ and the (unscaled) smoothness-penalty matrix $ \boldsymbol{\Omega}^{(2)}/M^{2} $ are constants that depend solely on the specified set of basis functions and do not vary with covariates $ \boldsymbol{Z}_{i} $ or outcomes $ \left\{Y_{ij},X_{ij}\right\} $. We have derived a closed-form expression for $ \boldsymbol{\Omega}^{(1)} $ when using natural cubic spline basis functions with $ K $ knots placed at $ t_{1},t_{2},\ldots, t_{K} $. For detailed mathematical formulations and proofs, please see Theorem S1 in Appendix SA of the Supplementary Material. The smoothness-penalty matrix $ \boldsymbol{\Omega}^{(2)}/M^{2} $ is commonly used in regularization for traditional smoothing spline methods (Wahba 1980; Parker and Rice 1985; Wahba et al. 1995; Wood 2011).

Consequently, we can compactly write the sparsity-smoothness penalty (2.2) as:


(2.3) \begin{align*}\mathcal{L}^{\mathrm{SSP}}(\boldsymbol{\theta})=\lambda\sum\limits_{p=1}^{P}\sqrt{\boldsymbol{\theta}_{p}^{T}\boldsymbol{H}_{\alpha}\boldsymbol{\theta}_{p}}\end{align*}

where $ \mathbf{H}_{\alpha}=(1-\alpha)\boldsymbol{\Omega}^{(1)}+\alpha\boldsymbol{\Omega}^{(2)} $. This formulation introduces a generalized group lasso penalty for any given $ \alpha $, aligning with the model proposed by Yuan and Lin (2006).

*Relations with other regularization methods.* The composite sparsity-smoothness penalty function in (2.3) represents a broad class of regularization methods applicable to high-dimensional generalized additive models. Specifically, setting $ \alpha\,=\,0 $ aligns the SSP penalty closely with the Sparse Additive Models (SpAM) introduced by Ravikumar et al. (2009) and the method by Wei et al. (2011). This formulation decouples the choice of smoother complexity from the sparsity constraint, which, while flexible, means the estimation accuracy could be highly sensitive to the choice of basis dimensions (also verified in Fig. 3A). Furthermore, when $ \boldsymbol{H}_{\alpha} $ equals an identity matrix, the SSP penalty simplifies to an ordinary group Lasso problem, mirroring the variable selection method described by Huang et al. (2010). In this case, the sparsity penalty is imposed directly on the basis coefficients $ \boldsymbol{\theta}_{p} $ other than the entire functional component $ \beta_{p}(t) $.

### 2.3. Penalized maximum likelihood

Let $ \boldsymbol{\theta} $ be the parameter vector to be estimated. It is the vectorization of $ (P\,+\,1)\times K $-dimensional coefficient matrix $ \boldsymbol{\Theta}=(\boldsymbol{\theta}_{0},\boldsymbol{\theta}_{1},\ldots , \boldsymbol{\theta}_{P})^{T} $ by row, ie $ \boldsymbol{\theta}=\mathrm{vech}(\boldsymbol{\Theta}) $. Our estimator for the sparse high-dimensional model defined in (2.1) and (2.3) is given by the following penalized maximum likelihood problem:


(2.4) \begin{align*}\widehat{\boldsymbol{\theta}}=\underset{\boldsymbol{\theta}}{\arg\min}\left\{\ell(\boldsymbol{\theta})+\lambda\sum\limits_{p=1}^{P}\sqrt{\boldsymbol{\theta}_{p}^{T}\boldsymbol{H}_{\alpha}\boldsymbol{\theta}_{p}}\right\},\end{align*}

where $ \ell(\boldsymbol{\theta}) $ is the twice negative log likelihood for model (2.1), expressed as


(2.5) \begin{align*}\ell(\boldsymbol{\theta})=-2\sum\limits_{i=1}^{N}\sum\limits_{j=1}^{m_{i}}\left\{{Y_{ij}}\log(\pi_{ij})+(X_{ij}-{Y_{ij}})\log(1-\pi_{ij})\right\}.\end{align*}

We refer to the estimate derived from (2.4) as the SSP estimator.

## 3. COMPUTATIONAL ALGORITHM

To solve the optimization problem in (2.4), we develop a proximal gradient descent algorithm. This algorithm addresses the non-differentiability of the penalty function and is noted for its scalability with high-dimensional predictors. In addition, we ensure convergence by carefully determining the step size via a backtracking line search strategy, which systematically fine-tunes the step size to maximize the reduction of the objective function in each iteration, thereby facilitating efficient and reliable convergence.

### 3.1. Proximal gradient descent algorithm

We start by decomposing the matrix $ \boldsymbol{H}_{\alpha} $ as $ \boldsymbol{H}_{\alpha}=\boldsymbol{L}_{\alpha}^{T}\boldsymbol{L}_{\alpha} $, where $ \boldsymbol{L}_{\alpha} $ is an upper triangular matrix with positive diagonal entries. Let $ \boldsymbol{\eta}_{p}=\boldsymbol{L}_{\alpha}\boldsymbol{\theta}_{p} $ and define $ \widetilde{\mathbb{X}}_{p}=\mathbb{X}_{p}\boldsymbol{L}_{\alpha}^{-1} $, where $ \mathbb{X}_{p} $ is the $ M\times K $ design matrix for the $ p $-th covariate, whose $ k $-th column is stacked with elements $ b_{k}(t_{ij})\times Z_{pi} $ where $ Z_{0i}\equiv 1 $. Using these new notations, the optimization problem (2.4) reduces to:


\begin{align*}\widehat{\boldsymbol{\eta}}=\mathop{\rm arg\, min}\limits_{\boldsymbol{\eta}}\left\{\ell(\boldsymbol{\eta})+\lambda\sum\limits_{p=1}^{P}\sqrt{\boldsymbol{\eta}_{p}^{T}\boldsymbol{\eta}_{p}}\right\}.\end{align*}

$ \ell(\boldsymbol{\eta}) $ is defined in (2.5), with $ \boldsymbol{\pi}=[1+\exp(\widetilde{\mathbb{X}}\boldsymbol{\eta})]^{-1} $ and $ \widetilde{\mathbb{X}}=\left[\widetilde{\mathbb{X}}_{0}\;|\;\widetilde{\mathbb {X}}_{1}\;|\ldots|\widetilde{\mathbb{X}}_{P}\right]\in\mathbb{R}^{M\times(P\,+\,1)K} $. The algorithm involves determining $ \widehat{\boldsymbol{\eta}}_{p} $ for given $ \lambda $ and $ \alpha $, and then calculating $ \widehat{\boldsymbol{\theta}}_{p}=\boldsymbol{L}_{\alpha}^{-1}\widehat{\boldsymbol{\eta}}_{p} $, for each $ p\,=\,0,1 , \ldots, P $.

*Iterative update for* $ \boldsymbol{\eta} $. Given $ \boldsymbol{\eta}^{(0)} $, we iteratively update $ \boldsymbol{\eta}^{(s)} $ using a proximal operator


(3.6) \begin{align*}\boldsymbol{\eta}^{(s)}\longleftarrow\mathrm{prox}_{t_{s}}\left[\boldsymbol{\eta}^{(s-1)}-t_{s}\nabla\ell(\boldsymbol{\eta}^{(s-1)})\right],\end{align*}

continuing until $ \boldsymbol{\eta} $ converges. The *proximal operator $ \mathrm{prox}_{f}:\mathbb{R}^{PK}\rightarrow\mathbb{R}^{PK} $* is defined as


\begin{align*}\mathrm{prox}_{t}(\mathbf{u})=\mathop{\rm arg\, min}\limits_{\boldsymbol{\eta}}\left(\frac{1}{2t}\|\mathbf{u}-\boldsymbol{\eta}\|_{2}^{2}+\lambda\sum\limits_{p=1}^{P}\sqrt{\boldsymbol{\eta}_{p}^{T}\boldsymbol{\eta}_{p}}\right).\end{align*}

Notably, the analytical solution to the proximal operator used in our model can be efficiently computed for each component of the vector $ \mathbf{u} $:


\begin{align*} [\mathrm{prox}_{t}(\mathbf{u})]_{p}=\left(1-\frac{t\lambda}{\sqrt{\mathbf{u}_{p}^{T}\mathbf{u}_{p}}}\right)_{+}\mathbf{u}_{p},\quad\mathrm{for}p=1 , \ldots, P.\end{align*}

The final proximal operator output $ \mathrm{prox}_{t}(\mathbf{u}) $ is thus $ \mathrm{prox}_{t}(\mathbf{u})=([\mathrm{prox}_{t}(\mathbf{u})]_{1}^{T},\ldots, [\mathrm{prox}_{t}(\mathbf{u})]_{p}^{T})^{T} $.

*Backtracking line search for the step size.* To guarantee convergence, the step size $ t_{s} $ for each iteration $ s $ is determined using a backtracking line search. We define the generalized gradient $ G_{t}(\boldsymbol{\eta}) $ as $ G_{t}(\boldsymbol{\eta})=\frac{1}{t}\left[\boldsymbol{\eta}-\mathrm{prox}_{t}(\boldsymbol{\eta}-t\nabla\ell(\boldsymbol{\eta}))\right] $. The update formula in (3.6) can then be written as $ \boldsymbol{\eta}^{(s)}=\boldsymbol{\eta}^{(s-1)}-t_{s}G_{t_{s}}(\boldsymbol{\eta}^{(s-1)}). $ We initialize $ t\,=\,t_{\mathrm{init}} > 0 $ and adjust $ t $ by multiplying by a factor $ \delta $ ($ 0 \lt\delta < 1 $), ie $ t\leftarrow\delta t $, until


\begin{align*}\ell\left(\boldsymbol{\eta}^{(s-1)}-tG_{t}(\boldsymbol{\eta}^{(s-1)})\right)\leq\ell(\boldsymbol{\eta}^{(s-1)})-t\nabla\ell(\boldsymbol{\eta}^{(s-1)})^{T} G_{t}(\boldsymbol{\eta}^{(s-1)})+\dfrac{t}{2}\|G_{t}(\boldsymbol{\eta}^{(s-1)})\|_{2}^{2}.\end{align*}

Once this condition is satisfied, we set $ t_{s}\leftarrow t $ and proceed to update $ \boldsymbol{\boldsymbol{\eta}}^{(s)} $ using the selected step size. The proposed overall estimating algorithm is summarized in Algorithm S1 in the Supplementary Material.

### 3.2. Choosing the tuning parameters

The algorithm in the previous section computes the estimates for $ \boldsymbol{\theta} $ for given values of tuning parameters $ \lambda $ and $ \alpha $. We use cross-validation (CV) to select the values of $ \lambda $ and $ \alpha $ by minimizing the averaged prediction errors in the validation sets, called mean CV errors. In our case, the prediction error in the validation set for the $ o $-th CV fold, $ \mathcal{V}^{o} $, is quantified by the mean deviance $ \dfrac{1}{M_{o}}\sum_{i, j\in\mathcal{V}^{o}}\left\{-2\left[{Y_{ij}}\log(\widehat{\pi}_{ij})+(X_{ij}-{Y_{ij}})\log(1-\widehat{\pi}_{ij})\right]\right\} $, where $ M_{o} $ is the total number of observations in $ \mathcal{V}^{o} $. In our R package sparseSOMNiBUS, we also allow users to select the value of $ \lambda $ based on the “one-standard-error” rule (1-SE-rule) (Friedman et al. 2010). Under the option of 1-SE-rule, we select the largest value of $ \lambda $ such that the mean CV error is within 1 SE of the minimum. This strategy generally favors parsimonious models. Figure S1 shows a simple example of the procedure for selecting the tuning parameter $ \lambda $ based on these criteria.

#### 3.2.1. Derive $ \lambda_{\max} $

For a given value of $ \alpha $, we can derive the smallest $ \lambda $ that gives the entire effect vector $ \boldsymbol{\widehat{\theta}}_{1}=\ldots=\boldsymbol{\widehat{\theta}}_{P}=\boldsymbol{0} $ in our optimization problem (2.4). This value is referred to as $ \lambda_{\max} $ in the regularization path for $ \lambda $. The derivation of $ \lambda_{\max} $ involves calculating the optimality conditions for the nonlinear programming problem in (2.4), specifically the Karush-Kuhn-Tucker (KKT) conditions. As explained in Appendix SB of the Supplementary Material, we determine that the smallest $ \lambda $ giving $ \boldsymbol{\theta}_{1}=\boldsymbol{\theta}_{2}=\ldots=\boldsymbol{\theta}_{p} =\boldsymbol{0} $ is


(3.7) \begin{align*}\lambda_{\max}=\max\limits_{p\in\{1,2 , \ldots, P\}}\left\{\sqrt{\boldsymbol{b}_{p}^{T}\boldsymbol{H}_{\alpha}^{-1}\boldsymbol{b}_{p}}\right\}.\end{align*}

In (3.7), $ \boldsymbol{b}_{p}=2\left[\mathbb{X}_{p}^{T}(\boldsymbol{Y}-\boldsymbol{\Lambda_{X}}{\boldsymbol{\pi}}_{0})\right] $ is the sub-vector of $ -\nabla\ell(\boldsymbol{\theta}) $ corresponding to $ \boldsymbol{\theta}_{p} $ evaluating from an intercept-only model. Here, $ \boldsymbol{Y}\in\mathbb{R}^{M} $ is the vector concatenating $ Y_{ij} $, $ \boldsymbol{\Lambda_{X}}\in\mathbb{R}^{M\times M} $ is the diagonal matrix with read-depth values $ X_{ij} $, and $ {\boldsymbol{\pi}}_{0}\in\mathbb{R}^{M} $ consists of elements $ [1+\exp({-\beta_{0}(t_{ij})})]^{-1} $.

#### 3.2.2. The warm start strategy

For a given $ \alpha $, we construct a sequence of $ L $ values for $ \lambda $ decreasing from $ \lambda_{\max} $ to $ \tau\lambda_{\max} $ on the log scale, where $ \tau $ is a small constant. The defaults in our package are set to $ L\,=\,100 $, $ \tau\,=\,0.01 $ if $ M < (P\,+\,1)K $, and $ \tau\,=\,0.001 $ if $ M\geq(P\,+\,1)K $, following Friedman et al. (2010). We then fit a sequence of models from $ \lambda_{\max} $ to $ \tau\lambda_{\max} $ using the warm start strategy (Friedman et al. 2007). That is, the solution for the $ l $-th $ \lambda $ is used as the initial value for the $ (l\,+\,1) $-th $ \lambda $. This strategy provides a good initialization for the optimization problem at a new $ \lambda $ and leads to considerable computational speedups.

## 4. THE ADAPTIVE SPARSITY-SMOOTHNESS PENALTY

Similar to the adaptive LASSO (Zou 2006), we can introduce weights to allow for different amounts of penalties for individual functional components in the model. Specifically, we define the adaptive sparsity-smoothness penalty function as


(4.8) \begin{align*}\mathcal{L}^{\mathrm{SSP, adp}}(\boldsymbol{\theta})=\lambda\sum\limits_{p=1}^{P}\sqrt{w_{1, p}(1-\alpha)J_{1}\left(\beta_{p}(t)\right)+w_{2, p}\alpha J_{2}\left(\beta_{p}(t)\right)},\end{align*}

where $ w_{1, p} $ and $ w_{2, p} $ are data-adaptive weights. A typical choice for the weights would be set


\begin{align*} w_{1, p}=\dfrac{1}{\sqrt{J_{1}\left(\widehat{\beta}_{p, int}(t)\right)}}\mathrm{and}w_{2, p}=\dfrac{1}{\sqrt{J_{2}\left(\widehat{\beta}_{p, int}(t)\right)}},\end{align*}

where $ \widehat{\beta}_{p, int}(t) $ is the ordinary SSP estimator. We then compute the estimator for $ \boldsymbol{\theta} $ similarly as described in Section 3. We refer to the estimator obtained from this adaptive approach as SSP estimator.

## 5. SIMULATION STUDY

We assessed our proposed estimator through simulations, comparing our general SSP estimator with its two variants: SSP0, which excludes smoothness penalty (ie $ \alpha\,=\,0 $), and the group LASSO (gLASSO) estimator, obtained by fixing $ \boldsymbol{H}_{\alpha}=\boldsymbol{I} $. We also compared these methods to the method implemented in mgcv (Wood 2017), which fits generalized additive models (GAM) without sparsity constraints. In some scenarios, we further evaluated the adaptive SSP variant and SSP with the 1-SE rule. For all sparsity-based approaches (SSP, SSP0 and gLASSO), tuning parameters were selected via 5-fold cross-validation. We used a grid of 100 values for $ \lambda $ (constructed as described in Section 3.2) and a grid of 12 values for $ \alpha $: $ [0 , 0.1 , \ldots , 0.9 , 0.95 , 0.99] $. Unless otherwise specified, functional parameters were expanded using natural cubic splines of rank $ K\,=\,10 $ across all methods.

### 5.1. Simulation design

**Fig. 2.**
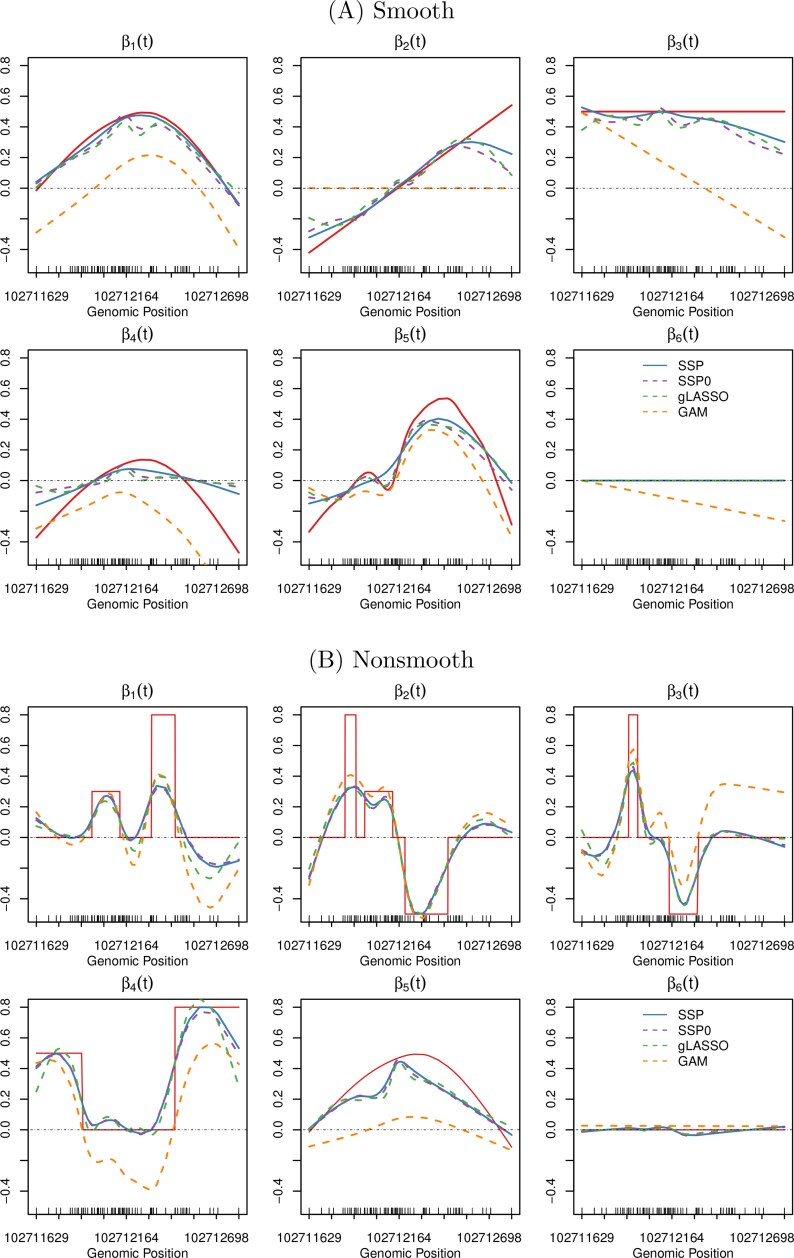
Estimates of the first 6 varying coefficients of one simulation run of A) Example 1 ($ P\,=\,100, \rho\,=\,0 $) and B) Example 2 ($ P\,=\,100, \rho\,=\,0 $), using the SSP, SSP0, group LASSO and GAM approaches. The red curves are the true $ \beta_{p}(t) $ used to generate the data. The results over 100 simulation runs are shown in Figs S1 to S4 for Example 1 and Figs S5 to S8 for Example 2.

Each simulation used a methylation region of 123 CpG sites, with locations matching those in the *BANK1* gene from Zhao et al. (2021). We varied the number of candidate SNPs ($ P\,=\,50,100,150,200,1000 $), the number of true mQTLs ($ P_{\mathrm{true}}=5 $ or 10), and the shape and magnitude of the effect curves $ \beta_{p}(t) $. Two settings for $ \beta_{p}(t),p\,=\,1 , \ldots P_{\mathrm{true}} $ were considered: **Example 1**: Smooth effects with varied shapes—constant, linear, quadratic-like, or bimodal for different SNPs (Fig. 2A), with nonzero effects spanning the full region. **Example 2**: Nonsmooth effects with sharp transitions (Fig. 2B), used to test robustness when smoothness assumption is violated.

To model linkage disequilibrium (LD), SNPs were simulated under a block-diagonal correlation structure with within-block correlation $ \rho\,=\,0 $, 0.3, or 0.7, representing no, moderate, and strong dependence. Each block contained 20 SNPs; for example, $ P\,=\,50 $ yields two blocks of 20 SNPs and one block of 10 SNPs. In the $ \rho > 0 $ settings, the $ P_{\mathrm{true}} $ true mQTLs were placed within a single block with pairwise correlation $ \rho $.

Complete simulation details are provided in Appendix SC, with parameter settings summarized in Table S3. Simulations were generated under the assumed model in (2.1). While these scenarios simplify real-world methylation (see Section 6) and LD structures, they provide a controlled framework for rigorous, interpretable evaluation of the proposed estimators.

### 5.2. Performance measures

We compared the performance of SSP, SSP0, group LASSO and GAM in terms of estimation accuracy, prediction error and variable selection accuracy. Note that GAM, implemented via the mgcv package, uses quadratic smoothness penalties and does not produce sparse solutions. Therefore, variable selection performance was accessed only for the sparsity-based approaches.

*Estimation.* To evaluate estimation accuracy, we computed Monte Carlo estimates of the integrated mean squared error (IMSE), integrated squared bias (IBIAS$ {}^{2} $), and integrated variance (IVAR) for each functional coefficient $ \beta_{p}(t) $. Let $ \left\{\widehat{\beta}^{(r)}(t),r\,=\,1 , \ldots, R\right\} $ denote estimates of $ \beta(t) $ from $ R $ simulation runs, omitting the subscript $ p $ for clarity. The simulation-based mean estimate is defined as $ \widehat{E}(t)=\dfrac{1}{R}\sum_{r\,=\,1}^{R}\widehat{\beta}^{(r)}(t) $. The three performance measures are then given by:


\begin{align*}\mathrm{IBIAS}^{2} & =\sum\limits_{t}\left\{\left[\widehat{E}(t)-\beta(t)\right]^{2}\right\},\;\mathrm{IVAR}=\sum\limits_{t}\left\{\dfrac{1}{R}\sum\limits_{r=1}^{R}\left[\widehat{\beta}^{(r)}(t)-\widehat{E}(t)\right]^{2}\right\},\\\text{and }\mathrm{IMSE} & =\sum\limits_{t}\left\{\dfrac{1}{R}\sum\limits_{r=1}^{R}\left[\widehat{\beta}^{(r)}(t)-\beta(t)\right]^{2}\right\}.\end{align*}

These quantities are computed separately for each $ \beta_{p}(t) $ in the model.

*Prediction.* We used the hold-out test sets to calculate four prediction measures—deviance errors, root mean square error (RMSE), and correlation between the predicted and observed proportions in the raw and transformed scales (shortly denoted as CorRaw and CorTrans, respectively). The deviance error is defined as $ \dfrac{1}{M}\sum_{i, j\in\mathrm{test}}\left[\ell(\widehat{\pi}_{ij}; Y_{ij},X_{ij})-\ell(\pi_{ij}; Y_{ij},X_{ij})\right] $, where $ \ell(\pi; Y_{ij},X_{ij})=-2\left[{Y_{ij}}\log(\pi)+(X_{ij}-{Y_{ij}})\log(1-\pi)\right] $, $ \widehat{\pi}_{ij} $ and $ {\pi}_{ij} $ are the predicted and true mean for the $ j $-th CpGs from the $ i $-th sample in the test set, respectively. We define the RMSE as $ \left\{\dfrac{1}{M}\sum_{i, j\in\mathrm{test}}\left[h(\widehat{\pi}_{ij})-h(Y_{ij}/X_{ij})\right]^{2}\right\}^{0.5} $, where $ h(\pi)=\arcsin(2\pi-1) $ is a variance stabilizing transformation of binomial variables (Korthauer et al. 2019). Similarly, CorRaw is calculated as the sample correlation between $ \widehat{\pi}_{ij} $ and $ Y_{ij}/X_{ij} $, and CorTran is the correlation between $ h(\widehat{\pi}_{ij}) $ and $ h(Y_{ij}/X_{ij}) $. We reported the mean and standard deviation (SD) of these four measures over all simulation runs to compare different methods.

*Selection.* We used the number of true positives (TP) and false positives (FP) at each simulation run for evaluating the variable selection performances.

### 5.3. Simulation results

An index of figures and tables by simulation setting and evaluation criterion is provided in Table S4.

#### 5.3.1. The role of the smoothness control in SSP

Figure 2A displays the estimated functions from one simulation run of Example 1 ($ P\,=\,100 , \rho\,=\,0 $). It clearly shows that when the true underlying function is **smooth**, estimates from SSP0 and gLASSO are too wiggly compared to the truth. Results across 100 simulation runs are shown in Figs S2 to S4, with corresponding BIAS$ {}^{2} $, IVAR and IMSE values reported in Table 1A. These results confirm that adding smoothness control reduces both estimation bias and variance compared to sparsity-only methods (SSP0 and gLASSO). This improvement is consistent under stronger SNP correlations ($ \rho > 0 $, Tables S5 and S6) and higher dimensionality ($ P\,=\,1000 $, Table S15). SSP also achieves smaller prediction error (top 2 panels of Table 1B and Table S9) and slightly better variable selection performance, with more TPs and fewer FPs (top 2 panels in Table 1C). This superiority remains in Examples 3 and 4 (smaller sample sizes and effect sizes than Example 1) under various combinations of $ P $ and $ P_{true} $ (see Tables S17 to S19 for estimation; Table 1B for prediction and Table 1C for selection).

**Table 1.** The accuracy of estimation, prediction, and variable selection with SSP, SSP0, group LASSO and GAM, under selected simulation examples and settings.

(A): Estimation: Example 1 (smooth, $ N=50,P_{true}=5,P=100,\rho=0 $)																
		IBIAS$ {}^{2} $				IVAR						IMSE				
		SSP	SSP0	gLASSO	GAM	SSP	SSP0	gLASSO	GAM			SSP	SSP0	gLASSO		GAM
$ \beta_{1}(t) $		0.260	0.497	0.589	4.681	0.182	0.284	0.320	3.745			0.441	0.781	0.910	8.426	
$ \beta_{2}(t) $		0.671	1.289	1.165	1.052	0.183	0.241	0.232	3.570			0.854	1.530	1.397	4.623	
$ \beta_{3}(t) $		0.567	1.114	1.081	12.110	0.287	0.439	0.467	8.101			0.855	1.554	1.548	20.211	
$ \beta_{4}(t) $		0.473	0.740	0.917	0.066	0.154	0.183	0.158	2.432			0.627	0.922	1.075	2.498	
$ \beta_{5}(t) $		0.951	0.961	1.026	0.473	0.298	0.368	0.365	2.174			1.249	1.329	1.391	2.647	
$ \beta_{6}(t) $		4.2e−05	1.6e−04	8.7e−05	3.5e−02	1.3e−02	1.4e−02	1.3e−02	1.800			1.3e−02	1.4e−02	1.3e−02	1.835	
$ \beta_{7}(t) $		2.0e−04	2.4e−04	7.7e−05	1.1e−02	6.8e−03	6.4e−03	5.3e−03	2.451			7.0e−03	6.6e−03	5.4e−03	2.462	
$ \beta_{8}(t) $		1.1e−04	1.6e−04	1.1e−04	7.6e−03	9.5e−03	9.4e−03	8.1e−03	2.014			9.6e−03	9.6e−03	8.2e−03	2.022	
$ \beta_{9}(t) $		1.2e−04	1.1e−04	1.3e−04	4.4e−02	7.2e−03	8.5e−03	7.2e−03	1.735			7.3e−03	8.6e−03	7.4e−03	1.779	
$ \beta_{10}(t) $		1.2e−04	1.1e−04	9.0e−05	1.3e−02	7.1e−03	7.7e−03	6.8e−03	2.228			7.2e−03	7.8e−03	6.9e−03	2.241	
$ {\sum_{1}^{100}} $ ^a^		2.931	4.613	4.787	21.429	1.939	2.464	2.401	225.656		4.870		7.077	7.188	247.085	

(B): Prediction						(C): Variable selection					
		Deviance				TP			FP		
	$ \rho $	SSP	SSP0	gLASSO	GAM	SSP	SSP0	gLASSO	SSP	SSP0	gLASSO
Example 1 (smooth, $ N=50,P_{true}=5,P=100 $)											
	0	0.026(0.010)	0.037(0.015)	0.038(0.016)	1.527(0.930)	5.00(0.00)	4.97(0.17)	4.97(0.17)	38.17(8.12)	42.66(7.41)	40.78(7.06)
	0.3	0.027(0.014)	0.037(0.018)	0.039(0.019)	1.573(1.135)	4.97(0.23)	4.94(0.28)	4.94(0.28)	34.68(7.84)	37.89(6.89)	36.97(6.72)
	0.7	0.026(0.013)	0.036(0.021)	0.040(0.024)	1.559(1.077)	4.95(0.22)	4.89(0.35)	4.88(0.36)	31.86(8.89)	35.73(7.39)	33.22(6.70)
Example 1 (smooth, $ N=50,P_{true}=5,P=1000 $)											
	0	0.049(0.027)	0.064(0.035)	0.068(0.038)	NA^b^	4.84(0.37)	4.80(0.41)	4.77(0.45)	82.51(16.07)	91.97(14.83)	90.57(15.12)
	0.3	0.043(0.031)	0.054(0.035)	0.058(0.036)	NA	4.91(0.28)	4.82(0.46)	4.80(0.50)	77.49(18.30)	83.24(14.41)	83.81(16.57)
Example 2 (nonsmooth, $ N=50,P_{true}=5,P=100 $)											
	0	0.175(0.039)	0.176(0.039)	0.165(0.037)	0.977(0.581)	5.00(0.00)	5.00(0.00)	5.00(0.00)	47.39(8.18)	46.94(8.15)	40.12(7.13)
	0.3	0.158(0.041)	0.160(0.042)	0.152(0.040)	1.235(0.615)	5.00(0.00)	5.00(0.00)	5.00(0.00)	46.68(6.67)	45.67(6.87)	38.96(7.17)
Example 2 (nonsmooth, $ N=50,P_{true}=5,P=1000 $)											
	0	0.204(0.066)	0.205(0.066)	0.196(0.066)	NA	4.98(0.14)	4.98(0.14)	4.98(0.14)	105.73(18.71)	101.63(18.00)	88.05(14.95)
$ P $	$ P_{true} $	Example 3 & 4 (smooth, low-magnitude effects, $ N=50,\rho=0 $)									
50	5	0.049(2e−02)	0.064(2e−02)	0.066(2e−02)	0.634(4e−01)	4.60(0.64)	4.42(0.76)	4.40(0.77)	16.48(4.79)	18.16(4.61)	16.77(4.24)
	10	0.148(5e−02)	0.169(5e−02)	0.166(4e−02)	0.690(3e−01)	8.54(1.09)	8.24(1.22)	8.23(1.25)	20.38(4.02)	21.14(3.40)	20.43(3.11)
100	5	0.071(3e−02)	0.080(3e−02)	0.081(3e−02)	0.593(2e−01)	4.22(0.84)	3.96(0.86)	3.91(0.93)	23.28(6.93)	24.38(6.76)	23.57(6.70)
	10	0.190(7e−02)	0.204(7e−02)	0.201(6e−02)	0.915(7e−01)	7.50(1.11)	7.28(1.20)	7.04(1.23)	30.66(5.32)	31.76(5.43)	30.85(6.23)
150	5	0.072(3e−02)	0.084(3e−02)	0.085(3e−02)	0.596(3e−01)	3.96(0.78)	3.72(0.81)	3.72(0.80)	27.56(6.00)	29.16(6.48)	28.13(6.80)
	10	0.192(7e−02)	0.202(6e−02)	0.198(6e−02)	0.918(4e−01)	6.62(1.28)	6.36(1.26)	6.36(1.17)	35.98(5.49)	37.94(5.35)	36.06(5.25)
200	5	0.087(4e−02)	0.098(4e−02)	0.096(4e−02)	0.598(3e−01)	3.82(0.87)	3.68(0.89)	3.62(0.90)	29.80(7.35)	30.84(7.07)	29.66(6.79)
	10	0.236(1e−01)	0.242(9e−02)	0.236(8e−02)	0.804(4e−01)	6.18(1.35)	5.80(1.34)	5.55(1.38)	39.80(7.33)	41.36(7.52)	39.34(7.35)
1000	5	0.108(4e−02)	0.115(4e−02)	0.117(4e−02)	NA	2.42(1.20)	2.34(1.14)	2.45(1.10)	45.28(11.28)	44.78(8.58)	64.91(138.89)
	10	0.272(8e−02)	0.272(7e−02)	8.565(4e+01)	NA	3.66(1.33)	3.36(1.44)	3.28(1.83)	60.64(10.74)	60.26(10.19)	74.13(137.10)

(A): IBIAS$ {}^{2} $, IVAR and IMSE of the first 10 varying coefficients. (B): Average (standard deviation) values of the deviance errors over 100 simulations. (C): Average (standard deviation) values of the number of TP and FP.

a Sum of the corresponding estimation measures across all varying coefficients in the model.

b GAM involves no sparsity regularizations and cannot estimate a model with 1000 smooth components for $ N\,=\,50 $.

**Fig. 3.**
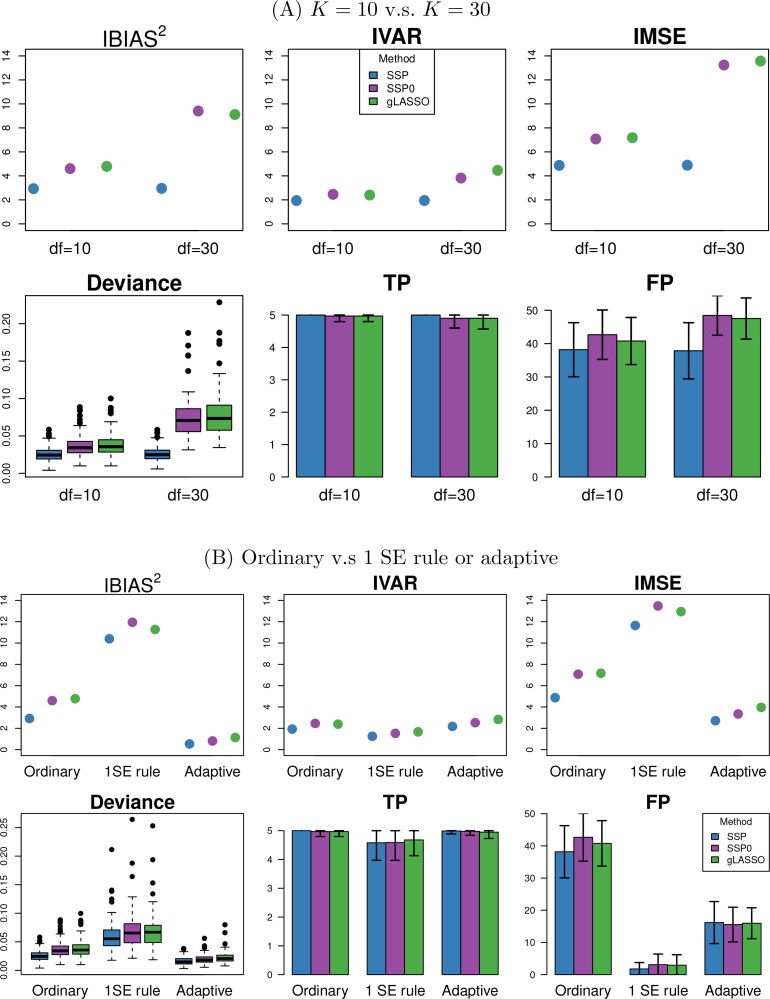
Performance measures A) of SSP, SSP0 and gLASSO when using 10 or 30 basis functions to expand $ \beta_{p}(t) $, labeled as “df = 10” and “df = 30,” and B) using the ordinary, 1SE rule and adaptive version of SSP, SSP0 and gLASSO. Data were generated from Example 1 ($ P_{true}=5, P\,=\,100, \rho\,=\,0 $). The top three panels show the values of IBIAS$ {}^{2} $, IVAR and IMSE aggregated from all the 100 varying coefficients in the model. The bottom left panel displays the distribution of deviance errors. The “TP” and “FP” panels display the mean values of TP and FP numbers, as well as their SD (indicated by the error bar), over 100 simulation runs.

Figure 3A further illustrates the role of smoothness control when the underlying functions are smooth. We compare the performance of SSP, SSP0, and gLASSO using a relatively large number of basis functions ($ K\,=\,30 $) to expand the $ \beta_{p}(t) $s. For reference, results based on $ K\,=\,10 $ are also shown. The performance of SSP0 and gLASSO deteriorates with $ K\,=\,30 $, showing increased estimation error, deviance, and false positives compared to their results at $ K\,=\,10 $.

In contrast, SSP—with its smoothness penalty—is less sensitive to the basis dimension and yields nearly identical results for $ K\,=\,10 $ and $ K\,=\,30 $. In practice, a large number of basis functions may be required to capture complex functional relationships, such as genetic effects across extended methylation regions. SSP remains robust in such cases, producing stable, smooth estimates through its refined smoothness control.

When the true underlying functions are **nonsmooth** (Example 2), the estimation results are similar across the three methods—SSP, SSP0 and gLASSO—as shown in Fig. 2B and Table S14. In this case, the benefit of adding the smooth control is minimal. All three methods exhibit considerable bias in estimating the nonsmooth $ \beta_{p}(t) $ functions and show greater prediction errors compared to their performances in Examples 1 (see Table 1B). This is expected, as splines are better suited for modeling smooth functions and less effective for irregular signals with spikes or abrupt changes. Nevertheless, variable selection performances are less affected. For instance, when $ P_{\rm true}=5 $ and $ P\,=\,1000 $, gLASSO identifies on average 4.98 TPs and 88.05 FPs—comparable to results from the smooth example (Table 1C). We also observe that gLASSO yields slightly lower prediction errors and fewer FPs than SSP and SSP0 in this nonsmooth setting.

#### 5.3.2. Sparsity-based methods outperform GAM

We compare the sparsity-based methods—SSP, SSP0, and gLASSO—with GAM to assess the role of sparsity regularization in high-dimensional varying coefficient models. Table 1A shows that, without sparsity constraints, GAM exhibits substantially higher estimation variance, a pattern consistent in the nonsmooth Example 2 (Table S14) and in Examples 3 and 4 with $ n\,=\,20 $ (Table S19). GAM also shows greater estimation bias than SSP overall, although the difference is less pronounced when the true functions are nonsmooth (Table S14) or when $ P $ is small (Table S19). In terms of prediction, GAM underperforms compared to sparsity-based methods (Table 1B; Tables S9 and S16). Moreover, GAM fails to fit models when $ P\gg N $ and cannot handle the case $ P\,=\,1000 $, while the sparsity-based approaches maintain reasonable prediction accuracy as $ P $ increases, as indicated by deviance errors (Table 1B). Finally, GAM with quadratic smoothness penalties does not shrink coefficients to zero and thus cannot perform variable selection. In contrast, Our approach maintains reasonable prediction and accuracies as $ P $ increases.

#### 5.3.3. Two extensions of SSP substantially improve variable selection accuracy

Figure 3B presents results from two types of extensions of SSP: the adaptive SSP (Section 4) and the use of the 1-SE rule for selecting $ \lambda $ based on cross-validation (Section 3.2). These extensions were also applied to the two special cases of SSP—SSP0 and gLASSO. Exact performance measures are reported in Tables S7, S8 and S10 to S13.

Overall, the adaptive versions outperform their ordinary counterparts, showing reduced estimation bias, prediction error, and the number of FPs, while maintaining similar estimation variance and numbers of TPs. As expected, applying the 1-SE-rule—compared to selecting the $ \lambda $ that minimizes the CV error—substantially reduces FPs, though it results in slightly increased estimation and prediction errors and a modest reduction in true positive counts.

*Summary and recommendation.* In settings where variable selection accuracy and model parsimony are prioritized, such as in our data application (Section 6), we recommend using adaptive SSP with tuning parameters selected via cross-validation under the 1-SE rule. This combination consistently reduces false positives while maintaining strong estimation and prediction performance, making it especially suitable for high-dimensional regional mQTL mapping.

## 6. DATA APPLICATION

The dataset includes 98 asymptomatic participants from the CARTaGENE cohort and focuses on associations between DNA methylation and anti-citrullinated protein antibody (ACPA) levels, a marker of rheumatoid arthritis risk. Our previous analysis (Zhao et al. 2024) did not account for genetic influences on methylation. We now implement a two-stage analysis: Stage I, using *sparseSOMNiBUS*, identifies regional mQTLs; Stage II tests ACPA-methylation associations while adjusting for the identified mQTLs. Full details on the Stage II model and results are provided in Appendix SD, along with information on the methylation platform and data processing.

### 6.1. Methodology for regional mQTL mapping

To capture biologically meaningful methylation patterns, we defined regions as the first exon plus 2,000bp upstream of each protein-coding gene. After filtering to retain regions with at least 20 CpGs, the analysis included 12,283 regions ($ \sim$ 1.4 million CpGs).

For each region, SNPs within a $\pm$ 2.5 Mb window were selected and pruned using the *snpgdsLDpruning* function from the SNPRelate R package with an LD threshold of $r^{2} < 0.2$. Figure S10 shows the distribution of the number of CpG sites (mean: 90.0; median: 74; IQR: 49 to 112) and pruned SNPs (mean: 64.3; median: 65; IQR: 56 to 73) per region. For a subset of genes, more relaxed thresholds (0.5, 0.8, and 0.9) were also evaluated (see Table S20 to S21; Figs S12 to S15).

We applied the adaptive SSP method to each region using a grid of 100 candidate $ \lambda $ values and smoothness parameters $ \alpha\in\{0,0.2,0.5,0.8,0.999\} $. Smooth terms were modeled using natural cubic splines. The spline basis dimension was fixed at 10 for the intercept function $ \beta_{0}(t) $ and set to the minimum of 10 and the number of CpGs divided by 10 for each genetic effect function. No penalty was applied to $ \beta_{0}(t) $. Tuning parameters were selected via 5-fold cross-validation using the 1-SE rule, with adaptive weights computed based on the initial ordinary SSP fit.

### 6.2. Results from regional mQTL mapping

**Table 2.** Top 20 gene-based methylation regions identified by sparseSOMNiBUS, ranked by the largest absolute effect estimate within the methylation region for the top SNP located in a $ \pm 2.5 $Mb window around each region.

	Gene-based methylation region								Top mQTL and effect summary							Overall model summary		
				Start	End	Region	Width	No. of	No. of	Top	SNP Pos	Subregion^a^	Peak CpG	SNP-CpG	Peak	No. of	% of	
Rank	Gene	Chr	Strand	(bp)	(bp)	Start-End from TSS	(bp)	CpGs	SNPs	SNP	from TSS	with $ |\beta(t)|\geq\log(2) $	Pos from TSS	Dis (bp)	$ \beta(t) $	mQTLs	mQTLs	$ \alpha $
1	LINC01252	12	+	11,699,056	11,701,293	[−1908, +329]	2237	73	70	rs6488428	−603	[−1157, −304]	−731	−128	3.173	46	65.71	0.5
2	RHOU	1	+	228,770,873	228,771,504	[−9521, −8890]	631	63	62	rs34530485	+1,657,936	[−9521, −9439]	−9521	−1,667,457	2.745	62	100	0
3	MIR4520-1	17	−	6,558,772	6,559,243	[−415, +56]	471	24	79	rs138292190	+1,790,881	[−343, +56]	+56	−1,790,825	2.293	74	93.67	0.999
4	IRGM	5	+	150,226,001	150,226,772	[−84, +687]	771	28	69	rs10067831	−41,514	[−14, +362]	+238	41,752	−2.246	34	49.28	0.999
5	ARRB2	17	+	4,611,262	4,614,031	[−2527, +242]	2769	142	80	rs113739199	+13,693	[−1541, −814]	−1113	−14,806	2.152	75	93.75	0.5
6	MRI1	19	+	13,873,186	13,875,525	[−2151, +188]	2339	120	74	rs1205169	−999,059	[−344, −106]	−200	998,859	−2.073	67	90.54	0
7	MIR4520-2	17	+	6,557,720	6,558,815	[−1048, +47]	1095	50	77	rs9905053	−91,311	[−752, +47]	−363	90,948	2.062	68	88.31	0.999
8	SDHAP3	5	−	1,594,510	1,595,407	[−15,712, −14,815]	897	76	76	rs73023414	−500,387	[−15,300, −14,815]	−14,852	485,535	1.942	75	98.68	0.2
9	LCLAT1	2	+	30,669,597	30,670,231	[−526, +108]	634	46	64	rs74910977	+107,411	[−526, −212]	−364	−107,775	−1.822	22	34.38	0.8
10	LINC02610	2	−	239,139,981	239,141,410	[−1092, +337]	1429	82	79	rs72992741	+6677	[−73, +92]	−32	−6709	−1.776	55	69.62	0
11	COMTD1	10	−	76,995,611	76,998,860	[−3090, +159]	3249	121	47	rs4746260	+153,526	[−2431, −1983]	−2211	−155,737	1.774	3	6.38	0.2
12	LINC01347	1	−	243,264,696	243,265,547	[−501, +350]	851	49	77	rs12121709	+1,771,372	[−174, +125]	−70	−1,771,442	1.663	77	100	0
13	SPRN	10	−	135,237,983	135,238,107	[+14, +138]	124	22	65	rs12243610	+612,428	[+14, +138]	+14	−612,414	1.654	64	98.46	0.5
14	PM20D1	1	−	205,819,039	205,819,649	[−373, +237]	610	32	68	rs56161922	−1,990,557	[−333, +237]	+237	1,990,794	−1.6	67	98.53	0.2
15	ADCY10P1	6	+	41,068,456	41,068,915	[−317, +142]	459	43	71	rs991762	−145,363	[−317, +142]	−317	14,5046	−1.578	55	77.46	0.8
16	CCDC92	12	−	124,457,079	124,459,702	[−29,594, −26,971]	2623	222	64	rs10773053	−149,977	[−27,711, −27,567]	−27,647	122,330	1.534	20	31.25	0
17	NLRP7	19	−	55,477,454	55,478,183	[−19,310, −18,581]	729	35	95	rs2914359	+1,687,189	[−19,310, −18,882]	−19,310	−1,706,499	−1.523	89	93.68	0.8
18	ZMAT5	22	−	30,162,947	30,164,438	[−1469, +22]	1491	67	58	rs5752590	+2,199,461	[−601, −373]	−496	−2199,957	1.522	20	34.48	0
19	SPRNP1	10	−	135,382,773	135,382,884	[+578, +689]	111	21	65	rs4880435	+734,133	[+578, +619]	+578	−733,555	1.516	63	96.92	0.5
20	NBEAP1	15	−	20,961,283	20,961,934	[−85,337, −84,686]	651	44	41	rs17766894	+294,357	[−84,954, −84,686]	−84,861	−379,218	1.501	35	85.37	0.8

Results are based on the adaptive SSP penalty, with regularization parameters selected via 5-fold cross-validation using the 1-SE rule. SNPs were LD-pruned ($ r^{2}\leq 0.2 $) to reduce computational complexity. Results under alternative LD thresholds (0.5, 0.8, 0.9) are provided in Table S20 to S21.

Abbreviations: chr: chromosome; bp: base pair; TSS: transcription start site; Pos: position; Pos from TSS: negative values indicate bases upstream of the TSS; Peak CpG: CpG site with the largest absolute effect associated with the top SNP; SNP-CpG Dis: the distance between the top SNP and peak CpG site. LD: linkage disequilibrium. $ r^{2} $: squared correlation coefficient between SNP genotypes.

a Subregion defined by contiguous positions where the absolute estimated effect exceeds $ \log(2) $, ie $ |\beta(t)|\geq\log(2) $.

We identified 1,014 regions with at least one mQTL. Table 2 lists the top 20 regions, ranked by the peak effect size of the top SNP. Distances between identified mQTLs and peak CpGs ranged from 63 bp to 2.5 Mb, with the distribution peaking below 50 kb and extending with a long, flat tail (Fig. S25), indicating enrichment of proximal associations alongside appreciable distal effects. Figure 1 and Fig. S11 illustrate representative regional patterns, where SNPs exhibited broad effects on methylation. Figure S19 shows the estimated functional coefficients for the intercept and top three SNPs, demonstrating the model’s ability to capture localized, position-specific genetic effects in a joint multivariable framework with adaptive SNP-specific penalties.

A key feature of sparseSOMNiBUS is its data-driven selection of the smoothness parameter $ \alpha $, enabling adaptive control over the complexity of fitted functional coefficients. With a sufficiently rich spline basis ($ K $), the method captures both smooth, localized peaks and broader regional effects when signals are strong and coordinated, though it may miss weak, isolated signals. Figures S17 to S18 show how varying $ \alpha $ influences effect estimation, demonstrating the method’s adaptability across signal types. Figure S20 shows that regions with more CpGs tended to select lower $ \alpha $ values, favoring flexible fits, while regions with detected mQTLs more often selected higher $ \alpha $, reflecting broader, structured methylation shifts. These patterns highlight the importance of adaptive smoothness control in capturing both localized and broad methylation effects.

To manage computational burden, we applied a stringent LD pruning threshold ($ r^{2} < 0.2 $) for the genome-wide scan and evaluated more relaxed thresholds (0.5, 0.8, and 0.9) for top regions. Higher thresholds increased model dimensionality (Table S20) and computation time (Table S21), but lead to fewer detected mQTLs per region (Table S20; Figs S12 to 14). This reduction is likely due to two factors: (i) increased SNP collinearity introduces redundancy, destabilizing selection and causing previously identified representative SNPs to drop out under penalization; and (ii) more conservative selection with a larger candidate SNP set, consistent with simulation results (Table 1C). Notably, top-ranked regions and lead SNPs remained largely stable across thresholds (Table S20; Figs S12 to S14), supporting the robustness of our findings to LD pruning choice.

*Computational scalability.* sparseSOMNiBUS exhibited favorable computational scaling: runtime increased approximately linearly with the number of SNPs, CpGs, and sample size, as shown by runtime measurements across real genomic regions (Fig. S15) and simulated data (Fig. S16), These results demonstrate its practical utility for methylation analysis across targeted regions or moderate-scale genome-wide scans.

## 7. DISCUSSION

We proposed a sparse high-dimensional generalized varying coefficient model for identifying genetic variants associated with regional DNA methylation levels. By applying separate penalties for sparsity and smoothness, our method simultaneously selects important mQTLs and estimates their effects across a methylation region, with estimation performed via a computationally efficient proximal gradient descent algorithm. Comprehensive simulations showed that incorporating smoothness control substantially improves results when the underlying effects are smooth. For irregular or spiky effect patterns, alternative basis functions such as Fourier or wavelets may yield better performance—a direction we leave for future work. Finally, we showed that combining sparsity and smoothness regularization yields estimates that are less sensitive to the choice of basis dimension.

Applied to the CARTaGENE data, sparseSOMNiBUS identified over 1,000 regions with at least one mQTL, recovering distinct patterns of SNP influence across methylation region. The analysis demonstrated the method’s ability to capture interpretable, region-specific genetic influences and highlighted the value of adaptive smoothness in modeling diverse signal patterns across real genomic regions.

The method is implemented in the R package sparseSOMNiBUS (https://github.com/kaiqiong/sparseSOMNiBUS), which fills a gap in existing software for fitting penalized regression models to *non-binary* binomial outcomes. The package also supports a flexible class of penalty functions, including the general sparsity-smoothness penalty (SSP), sparsity-only penalty (SSP0), and group LASSO, providing users with customizable modeling options.

Our method relies on the assumption that observed methylated read counts accurately represent the underlying methylation status. In practice, errors from incomplete bisulfite conversion or other sequencing artifacts may contaminate the data. Such measurement error is unlikely to impact variable selection—since covariates with zero effect on the true outcomes are not predictive of the mismeasured outcomes either—but it can bias the estimation of nonzero varying effects. A promising future direction is to extend our high-dimensional model to account for mismeasured outcomes. Building on the error models proposed in Zhao et al. (2021, 2024), this would involve incorporating sparsity penalties into hierarchical binomial regression frameworks with latent outcome structures.

Another potential restriction of our method lies in its distributional assumptions for the outcome. In practice, methylation data may exhibit relative to the binomial model, for example, when counts follow a beta-binomial distribution. It would be valuable to evaluate the robustness of our approach under such conditions through additional simulationoverdispersions. Extending our framework to allow quasi-likelihood-based variable selection represents another promising direction, as it would relax distributional assumptions while retaining penalized regression structure. Moreover, given the well-known equivalence between smoothness penalties and Gaussian random effects (Wahba 1983; Silverman 1985), the square-root penalty formulation could also be adapted for random effect selection in mixed effect models.

## Supplementary Material

kxaf032_Supplementary_Data

## Data Availability

Simulation code is available at: https://github.com/kaiqiong/sparseSOMNiBUS_simu.

## References

[kxaf032-B1] Affinito O et al. 2020. Nucleotide distance influences co-methylation between nearby CpG sites. Genomics. 112:144–150. 31078719 10.1016/j.ygeno.2019.05.007

[kxaf032-B2] Barber RF , Reimherr M, Schill T. 2017. The function-on-scalar LASSO with applications to longitudinal GWAS. Electron J Stat. 11:1351–1389.

[kxaf032-B3] Cheung WA et al. 2017. Functional variation in allelic methylomes underscores a strong genetic contribution and reveals novel epigenetic alterations in the human epigenome. Genome Biol. 18:50–21. 28283040 10.1186/s13059-017-1173-7 PMC5346261

[kxaf032-B4] Chouldechova A , Hastie T. 2015. Generalized additive model selection [preprint]. arXiv, arXiv:1506.03850.

[kxaf032-B5] Eckhardt F et al. 2006. DNA methylation profiling of human chromosomes 6, 20 and 22. Nat Genet. 38:1378–1385. 17072317 10.1038/ng1909 PMC3082778

[kxaf032-B6] Fan J , Ma Y, Dai W. 2014. Nonparametric independence screening in sparse ultra-high-dimensional varying coefficient models. J Am Stat Assoc. 109:1270–1284. 25309009 10.1080/01621459.2013.879828 PMC4188418

[kxaf032-B7] Fan Y et al. 2019. IMAGE: high-powered detection of genetic effects on DNA methylation using integrated methylation QTL mapping and allele-specific analysis. Genome Biol. 20:220–18. 31651351 10.1186/s13059-019-1813-1 PMC6813132

[kxaf032-B8] Friedman J , Hastie T, Höfling H, Tibshirani R. 2007. Pathwise coordinate optimization. Ann Appl Stat. 1:302–332.

[kxaf032-B9] Friedman J , Hastie T, Tibshirani R. 2010. Regularization paths for generalized linear models via coordinate descent. J Stat Softw. 33:1–22. 20808728 PMC2929880

[kxaf032-B10] Gaunt TR et al. 2016. Systematic identification of genetic influences on methylation across the human life course. Genome Biol. 17:61–14. 27036880 10.1186/s13059-016-0926-z PMC4818469

[kxaf032-B11] Gertheiss J , Maity A, Staicu AM. 2013. Variable selection in generalized functional linear models. Stat. 2:86–103. 25132690 10.1002/sta4.20 PMC4131701

[kxaf032-B12] Hannon E et al. 2018. Characterizing genetic and environmental influences on variable DNA methylation using monozygotic and dizygotic twins. PLoS Genet. 14:e1007544. 30091980 10.1371/journal.pgen.1007544 PMC6084815

[kxaf032-B13] Hannon E et al. 2016. Methylation QTLs in the developing brain and their enrichment in schizophrenia risk loci. Nat Neurosci. 19:48–54. 26619357 10.1038/nn.4182 PMC4714325

[kxaf032-B14] Huang J , Horowitz JL, Wei F. 2010. Variable selection in nonparametric additive models. Ann Stat. 38:2282–2313. 21127739 10.1214/09-AOS781 PMC2994588

[kxaf032-B15] Kim R , Müller S, Garcia TP. 2021. svReg: Structural varying‐coefficient regression to differentiate how regional brain atrophy affects motor impairment for Huntington disease severity groups. Biom J. 63:1254–1271. 33871905 10.1002/bimj.202000312 PMC9012319

[kxaf032-B16] Korthauer K et al. 2019. Detection and accurate false discovery rate control of differentially methylated regions from whole genome bisulfite sequencing. Biostatistics. 20:367–383. 29481604 10.1093/biostatistics/kxy007 PMC6587918

[kxaf032-B17] Lin Y , Zhang HH. 2006. Component selection and smoothing in multivariate nonparametric regression. Ann Stat. 34:2272–2297.

[kxaf032-B18] Meier L , Van de Geer S, Bühlmann P. 2009. High-dimensional additive modeling. Ann Stat. 37:3779–3821.

[kxaf032-B19] Noh HS , Park BU. 2010. Sparse varying coefficient models for longitudinal data. Stat Sin. 20:1183–1202.

[kxaf032-B20] Park S , Oh HS, Lee J. 2023. Lévy adaptive B-spline regression via overcomplete systems. Stat Sin. 33:2715–2737.

[kxaf032-B21] Parker R , Rice J. 1985. Discussion on “some aspects of the spline smoothing approach to non-parametric regression curve fitting” (by B. W. Silverman). J R Stat Soc Series B Stat Methodol. 47:40–42.

[kxaf032-B22] Ravikumar P , Lafferty J, Liu H, Wasserman L. 2009. Sparse additive models. J R Stat Soc Series B Stat Methodol. 71:1009–1030.

[kxaf032-B23] Schmitz RJ et al. 2013. Patterns of population epigenomic diversity. Nature. 495:193–198. 23467092 10.1038/nature11968 PMC3798000

[kxaf032-B24] Silverman BW. 1985. Some aspects of the spline smoothing approach to non-parametric regression curve fitting. J R Stat Soc Series B Stat Methodol. 47:1–21.

[kxaf032-B25] Taylor DL et al. 2019. Integrative analysis of gene expression, DNA methylation, physiological traits, and genetic variation in human skeletal muscle. Proc Natl Acad Sci U S A. 116:10883–10888. 31076557 10.1073/pnas.1814263116 PMC6561151

[kxaf032-B26] Tibshirani R , Friedman J. 2020. A pliable lasso. J Comput Graph Stat. 29:215–225. 36340327 10.1080/10618600.2019.1648271 PMC9631466

[kxaf032-B27] Van Dongen J , BIOS Consortium et al. 2016. Genetic and environmental influences interact with age and sex in shaping the human methylome. Nat Commun. 7:11115–13. 27051996 10.1038/ncomms11115 PMC4820961

[kxaf032-B28] Wahba G. 1980. Spline bases, regularization, and generalized cross-validation for solving approximation problems with large quantities of noisy data. Approx Theory III. 2:905–912.

[kxaf032-B29] Wahba G. 1983. Bayesian “confidence intervals” for the cross-validated smoothing spline. J R Stat Soc Series B Stat Methodol. 45:133–150.

[kxaf032-B30] Wahba G et al. 1995. Smoothing spline ANOVA for exponential families, with application to the Wisconsin epidemiological study of diabetic retinopathy: the 1994 Neyman Memorial Lecture. Ann Stat. 23:1865–1895.

[kxaf032-B31] Wang H , Xia Y. 2009. Shrinkage estimation of the varying coefficient model. J Am Stat Assoc. 104:747–757.

[kxaf032-B32] Wang L , Chen G, Li H. 2007. Group SCAD regression analysis for microarray time course gene expression data. Bioinformatics. 23:1486–1494. 17463025 10.1093/bioinformatics/btm125

[kxaf032-B33] Wang L , Li H, Huang JZ. 2008. Variable selection in nonparametric varying-coefficient models for analysis of repeated measurements. J Am Stat Assoc. 103:1556–1569. 20054431 10.1198/016214508000000788 PMC2801925

[kxaf032-B34] Wang X , Jiang B, Liu JS. 2024. Varying coefficient model via adaptive spline fitting. J Comput Graph Stat. 33:614–624.

[kxaf032-B35] Wei F , Huang J, Li H. 2011. Variable selection and estimation in high-dimensional varying-coefficient models. Stat Sin. 21:1515–1540. 24478564 10.5705/ss.2009.316 PMC3902862

[kxaf032-B36] Wood SN. 2011. Fast stable restricted maximum likelihood and marginal likelihood estimation of semiparametric generalized linear models. J R Stat Soc Series B Stat Methodol. 73:3–36.

[kxaf032-B37] Wood SN. 2017. Generalized additive models: an introduction with R. CRC Press.

[kxaf032-B38] Xue L , Qu A. 2012. Variable selection in high-dimensional varying-coefficient models with global optimality. J Mach Learn Res. 13:1973–1998.

[kxaf032-B39] Yuan M , Lin Y. 2006. Model selection and estimation in regression with grouped variables. J R Stat Soc Series B Stat Methodol. 68:49–67.

[kxaf032-B40] Zhao K et al. 2021. A novel statistical method for modeling covariate effects in bisulfite sequencing derived measures of DNA methylation. Biometrics. 77:424–438. 32438470 10.1111/biom.13307 PMC8359306

[kxaf032-B41] Zhao K et al. 2024. Addressing dispersion in mis-measured multivariate binomial outcomes: a novel statistical approach for detecting differentially methylated regions in bisulfite sequencing data. Stat Med. 43:3899–3920. 38932470 10.1002/sim.10149

[kxaf032-B42] Zhou X , Stephens M. 2014. Efficient multivariate linear mixed model algorithms for genome-wide association studies. Nat Methods. 11:407–409. 24531419 10.1038/nmeth.2848 PMC4211878

[kxaf032-B43] Zou H. 2006. The adaptive lasso and its oracle properties. J Am Stat Assoc. 101:1418–1429.

