# Supplementary material for: A novel high-dimensional model for identifying regional DNA methylation QTLs
Source: Biostatistics. 2025 Oct 26;26(1):kxaf032. doi: 10.1093/biostatistics/kxaf032 (PMC12554007; doi:10.1093/biostatistics/kxaf032)
Supplement: kxaf032_Supplementary_Data [file kxaf032_supplementary_data.pdf]

Supplementary material to "A high-dimensional model for identifying regional DNA methylation QTLs"

Appendix

A Natural cubic spline and its sparsity-penalty matrix $\mathbf{\Omega^{(1)}}$

Splines are polynomial pieces jointed at certain values (i.e. knots). Cubic spline is the most commonly used one, which is represented by piecewise cubic polynomial with continuous first and second derivatives at the knots. To avoid erratic behaviors (high variance) of cubic fit near the boundaries, a natural cubic spline adds additional constrains that the function is linear beyond the two end-points. In this work, we use natural cubic regression spline to represent the functional parameters $\beta_p(t_{ij})$. Without loss of generality, we drop the subscript p from the notations here and consider defining a natural cubic spline function $\beta(t)$, with K given knots, $t_1, t_2, \ldots t_K$.

There are many equivalent bases definitions that can be used to expand the cubic spline $\beta(t)$. We adopt the basis used in the R package `mgcv` (Wood, 2017), where the spline is parameterized in terms of its values at the knots. One advantage of this basis definition is that it does not require any re-scaling of the predictor variable t. Let $\theta_j = \beta(t_j)$ and $\delta_j = \beta''(t_j)$, $j = 1, 2, \ldots K$. Then the spline $\beta(t)$ can be written as

$$\beta(t) = a_j^-(t)\theta_j + a_j^+(t)\theta_{j+1} + c_j^-(t)\delta_j + c_j^+(t)\delta_{j+1}, \text{ if } t_j \le t \le t_{j+1}. \tag{A.1}$$

Let $h_j = t_{j+1} - t_j$, the 'basis' functions a_j^-, a_j^+, c_j^- and c_j^+ in (A.1) are defined as

$$\begin{aligned} a_j^-(t) &= \frac{t_{j+1} - t}{h_j}, \quad c_j^-(t) = \frac{1}{6}\left[\frac{(t_{j+1} - t)^3}{h_j} - h_j(t_{j+1} - t)\right], \\ a_j^+(t) &= \frac{t - t_j}{h_j}, \quad c_j^+(t) = \frac{1}{6}\left[\frac{(t - t_j)^3}{h_j} - h_j(t - t_j)\right]. \end{aligned} \tag{A.2}$$

As explained in Section 2.3, a core part in the sparsity-penalty is the squared L2-norm of the function $\beta(t)$, which can be written as a quadratic form in terms of $\boldsymbol{\theta}$ and penalty matrix $\mathbf{\Omega^{(1)}}$,

$$\int_{t_1}^{t_K} (\beta(t))^2 \, dt = \boldsymbol{\theta}^T \mathbf{\Omega^{(1)}} \boldsymbol{\theta}.$$

In the rest of this section, $\mathbf{\Omega^{(1)}}$ is derived under the basis representation in (A.1).

A.1 Relation between spline values $\boldsymbol{\theta}$ and their second derivatives $\boldsymbol{\delta}$

The conditions that the spline should be continuous to second derivatives, at each interior knots t_j, and have zero second derivative at t_1 and t_K imply a deterministic relation between function values $\boldsymbol{\theta} = (\theta_1, \dots \theta_K)$ and second derivatives $\boldsymbol{\delta} = (\delta_1, \dots, \delta_K)$,

$$\boldsymbol{\delta} = \boldsymbol{F}\boldsymbol{\theta}. \tag{A.3}$$

The mapping matrix $\boldsymbol{F} \in \mathcal{R}^{K \times K}$ takes the form

$$\boldsymbol{F} = \begin{bmatrix} \mathbf{0} \\ \boldsymbol{B^{-1}D} \\ \mathbf{0} \end{bmatrix}, \tag{A.4}$$

where matrices $\boldsymbol{B} \in \mathcal{R}^{(K-2)\times(K-2)}$ and $\boldsymbol{D} \in \mathcal{R}^{(K-2)\times K}$ have non-zero elements,

$$\begin{aligned}
&D_{i,i} = \frac{1}{h_i}, \qquad D_{i,i+1} = -\frac{1}{h_i} - \frac{1}{h_{i+1}}, \qquad D_{i,i+2} = \frac{1}{h_{i+1}} \\
&B_{i,i} = \frac{h_i + h_{i+1}}{3}, \qquad i = 1, \dots k-2 \\
&B_{i,i+1} = \frac{h_{i+1}}{6} \qquad B_{i+1,i} = \frac{h_{i+1}}{6} \qquad i = 1, \dots k-3.
\end{aligned}$$

The detailed derivation for (A.4) can be found in (Wood, 2017, Section 5.3.1). Thus, the expansion in (A.1) can be rewritten entirely in terms of $\boldsymbol{\theta}$ as

$$\beta(t) = a_j^-(t)\theta_j + a_j^+(t)\theta_{j+1} + c_j^-(t)\boldsymbol{F_j}\boldsymbol{\theta} + c_j^+(t)\boldsymbol{F_{j+1}}\boldsymbol{\theta}, \text{ if } t_j \le t \le t_{j+1},$$

where $\boldsymbol{F_j}$ is the jth row of matrix $\boldsymbol{F}$. The expansion can be further expressed in a more compact way, $\beta(t) = \sum_{i=1}^K b_i(t)\theta_i$, where basis functions $b_i(t)$ are

$$b_i(t) = \begin{cases} a_{i-1}^+(t) + c_{i-1}^-(t)F_{i-1,i} + c_{i-1}^+(t)F_{i,i} & \text{if} \quad t_{i-1} \le t \le t_i \\ a_i^-(t) + c_i^-(t)F_{i,i} + c_i^+ F_{i+1,i} & \text{if} \quad t_i \le t \le t_{i+1} \\ c_k^-(t)F_{k,i} + c_k^+ F_{k+1,i} & \text{if} \quad t_k \le t \le t_{k+1}, \text{ and } k \ne i \text{ or } i-1. \end{cases} \tag{A.5}$$

Writing $\boldsymbol{b}(t)$ as the vector with i^{th} element $b_i(t)$, it is easy to show that

$$\int_{t_1}^{t_K} (\beta(t))^2 \, dt = \boldsymbol{\theta}^T \left\{ \int_{t_1}^{t_K} \boldsymbol{b}(t)\boldsymbol{b}(t)^T dt \right\} \boldsymbol{\theta},$$

which immediately implies $\boldsymbol{\Omega^{(1)}} = \int_{t_1}^{t_K} \boldsymbol{b}(t)\boldsymbol{b}(t)^T dt$. However, it is quite complicated, although possible, to evaluate this integral analytically, because $b_i(t)$ takes non-zero values in each of the intervals between two knots. To mitigate the problem, instead of working on the expansion solely in terms of $\boldsymbol{\theta}$, we seek to calculate the integral based on the expansion in terms of both $\boldsymbol{\theta}$ and $\boldsymbol{\delta}$, i.e. the expression in (A.1). Then, we can determine the sparsity penalty matrix $\boldsymbol{\Omega^{(1)}}$ by transforming $\boldsymbol{\delta}$ back to $\boldsymbol{\theta}$.

A.2 L2-norm of a natural cubic spline

Theorem 1. *Suppose $\beta(t)$ is a natural cubic spline function, with K knots at $t_1, t_2, \ldots t_K$, and basis expansion defined in (A.1). We have the following result for the L2-norm of $\beta(t)$,*

$$\|\beta(t)\|_2^2 = \int_{t_1}^{t_K} (\beta(t))^2 \, dt = \boldsymbol{\theta}^T \boldsymbol{\Omega}^{(\mathbf{1})} \boldsymbol{\theta}.$$

Here $\boldsymbol{\theta}$ is a vector with basis coefficients for $\theta_j = \beta(t_j)$, for $j = 1, 2, \ldots K$ and $\boldsymbol{\Omega}^{(\mathbf{1})}$ takes the form

$$\boldsymbol{\Omega}^{(\mathbf{1})} = \boldsymbol{A}_{11} + \boldsymbol{F}^T \boldsymbol{A_{12}}^T + \boldsymbol{A}_{12}\boldsymbol{F} + \boldsymbol{F}^T \boldsymbol{A}_{22}\boldsymbol{F}.$$

Specifically, $\boldsymbol{A}_{11}, \boldsymbol{A}_{12}, \boldsymbol{A}_{22} \in \mathbb{R}^{K \times K}$ are tri-diagonal matrices with elements defined in Table S1, and $\boldsymbol{F}$ is the matrix mapping the function values at the knots onto their second derivatives, as given in (A.4).

Table S1. Elements in the tri-diagonal matrices $\boldsymbol{A}_{11}, \boldsymbol{A}_{12}, \boldsymbol{A}_{22} \in \mathbb{R}^K$, which are used to define the L2-norm of natural cubic spline. $h_i = t_{i+1} - t_i$.

	$(1,1)$	(i,i), $i = 2, 3, \ldots K-1)$	(K,K)	$(i, i-1)$ and $(i-1, i)$
$\boldsymbol{A}_{11}$	$\frac{h_1}{3}$	$\frac{h_{i-1}}{3} + \frac{h_i}{3}$	$\frac{h_{K-1}}{3}$	$\frac{h_{i-1}}{6}$
$\boldsymbol{A}_{12}$	$-\frac{h_1^3}{45}$	$-\frac{h_{i-1}^3}{45} - \frac{h_i^3}{45}$	$-\frac{h_{K-1}^3}{45}$	$-\frac{7}{360} h_{i-1}^3$
$\boldsymbol{A}_{22}$	$\frac{4}{315} h_1^5$	$\frac{4}{315}\left(h_{i-1}^5 + h_i^5\right)$	$\frac{4}{315} h_{K-1}^5$	$\frac{31}{15120} h_{i-1}^5$

Proof. The basis expansion of $\beta(t)$ in (A.1) can be re-expressed as

$$\beta(t) = \sum_{i=1}^{K} d_i(t)\theta_i + \sum_{i=1}^{K} e_i(t)\delta_i,$$

where the sets of 'basis' functions $d_i(t)$ and $e_i(t)$ are defined in the Table S2

Evaluating the integral involving $d_i(t)$ and $e_i(t)$ is much easier compared to evaluating $b_i(t)$, because $d_i(t)$ and $e_i(t)$ are non-zero over no more than 2 consecutive intervals.

Concatenate the coefficients vectors $\boldsymbol{\theta}$ and $\boldsymbol{\delta}$ into a $2K-$ dimensional vector $\boldsymbol{\alpha} = (\boldsymbol{\theta}, \boldsymbol{\delta})^T$ and define $\boldsymbol{q}(t)$ as the basis vector joining $d_i(t)$ and $e_i(t)$, $\boldsymbol{q}(t) = (d_1(t), \ldots d_K(t), e_1(t), \ldots e_K(t))^T$. We can thus rewrite the L2-norm of $\beta(t)$ as

$$\int_{t_1}^{t_K} (\beta(t))^2 \, dt = \boldsymbol{\alpha}^T \int_{t_1}^{t_K} \boldsymbol{q}(t)\boldsymbol{q}(t)^T dt \, \boldsymbol{\alpha}. \tag{A.6}$$

Table S2. Definitions of basis functions $d_i(t)$ and $e_i(t)$ used to define a natural cubic regression spline $\beta(t)$.

	$i=1$	$i=2,3,\dots K-1$	$i=K$
$d_i(t) =$	$a_1^-(t)\mathbb{1}(t_1 \le t \le t_2)$	$a_{i-1}^+(t)$ if $t_{i-1} \le t \le t_i$<br>$a_i^-(t)$ if $t_i \le t \le t_{i+1}$<br>0 otherwise	$a_{K-1}^+(t)\mathbb{1}(t_{K-1} \le t \le t_K)$
$e_i(t) =$	$c_1^-(t)\mathbb{1}(t_1 \le t \le t_2)$	$c_{i-1}^+(t)$ if $t_{i-1} \le t \le t_i$<br>$c_i^-(t)$ if $t_i \le t \le t_{i+1}$<br>0 otherwise	$c_{K-1}^+(t)\mathbb{1}(t_{K-1} \le t \le t_K)$

It is clear that $\int_{t_1}^{t_K} \boldsymbol{q}(t)\boldsymbol{q}(t)^T dt$ is symmetric, by construction, and consists of four blocks, $\boldsymbol{A}_{11}, \boldsymbol{A}_{12}, \boldsymbol{A}_{12}^T$ and $\boldsymbol{A}_{22}$, as defined below,

$$\int_{t_1}^{t_K} \boldsymbol{q}(t)\boldsymbol{q}(t)^T dt = \begin{bmatrix} \int_{t_1}^{t_K} \boldsymbol{d}(t)\boldsymbol{d}(t)^T dt & \int_{t_1}^{t_K} \boldsymbol{d}(t)\boldsymbol{e}(t)^T dt \\ \int_{t_1}^{t_K} \boldsymbol{e}(t)\boldsymbol{d}(t)^T dt & \int_{t_1}^{t_K} \boldsymbol{e}(t)\boldsymbol{e}(t)^T dt \end{bmatrix} := \begin{bmatrix} \boldsymbol{A}_{11} & \boldsymbol{A}_{12} \\ \boldsymbol{A}_{12}^T & \boldsymbol{A}_{22} \end{bmatrix}.$$

Calculating $\boldsymbol{A}_{11}$ $\boldsymbol{A}_{11}$ is tri-diagonal because each $d_i(t)$ is non-zero over only 2 intervals. The ith leading diagonal element, for $i = 2, \dots K-1$, is given by

$$\begin{aligned}[\boldsymbol{A}_{11}]_{i,i} = \int_{t_1}^{t_K} d_i(t)^2 dt &= \int_{t_{i-1}}^{t_i} \left(\frac{t-t_{i-1}}{h_{i-1}}\right)^2 dt + \int_{t_i}^{t_{i+1}} \left(\frac{t_{i+1}-t}{h_i}\right)^2 dt \\ &= \left.\frac{(t-t_{i-1})^3}{3h_{i-1}^2}\right|_{t_{i-1}}^{t_i} - \left.\frac{(t_{i+1}-t)^3}{3h_i^2}\right|_{t_i}^{t_{i+1}} = \frac{h_{i-1}}{3} + \frac{h_i}{3}.\end{aligned}$$

The first and last leading diagonal elements are

$$[\boldsymbol{A}_{11}]_{1,1} = \frac{h_1}{3} \text{ and } [\boldsymbol{A}_{11}]_{K,K} = \frac{h_{K-1}}{3}.$$

Similarly, the off-diagonal elements $[\boldsymbol{A}_{11}]_{(i-1,i)}$ and $[\boldsymbol{A}_{11}]_{(i,i-1)}$, where $i = 2, \dots K$, are given by

$$\int_{t_1}^{t_K} d_i(t)d_{i-1}(t)dt = \int_{t_{i-1}}^{t_i} a_{i-1}^+(t)a_{i-1}^-(t)dt = \int_{t_{i-1}}^{t_i} \left(\frac{t-t_{i-1}}{h_{i-1}}\right)\left(\frac{t_i-t}{h_{i-1}}\right) dt = \frac{h_{i-1}}{6}.$$

Calculating $\boldsymbol{A}_{12}$ $\boldsymbol{A}_{12}$ is also tri-diagonal because both $d_i(t)$ and $e_i(t)$ are non-zero over only 2 intervals. The ith leading diagonal element, for $i = 2, \dots K-1$, is given by

$$\begin{aligned}
[\boldsymbol{A}_{12}]_{i,i} = \int_{t_1}^{t_K} d_i(t)e_i(t)dt &= \int_{t_{i-1}}^{t_i} a_{i-1}^{+}(t)c_{i-1}^{+}(t)dt + \int_{t_i}^{t_{i+1}} a_i^{-}(t)c_i^{-}(t)dt \\
&= \int_{t_{i-1}}^{t_i} \left(\frac{t-t_{i-1}}{h_{i-1}}\right)\frac{1}{6}\left[\frac{(t-t_{i-1})^3}{h_{i-1}} - h_{i-1}(t-t_{i-1})\right]dt \\
&\quad + \int_{t_i}^{t_{i+1}} \left(\frac{t_{i+1}-t}{h_i}\right)\frac{1}{6}\left[\frac{(t_{i+1}-t)^3}{h_i} - h_i(t_{i+1}-t)\right]dt \\
&= -\frac{h_{i-1}^3}{45} - \frac{h_i^3}{45}
\end{aligned}$$

The first and last leading diagonal elements of $\boldsymbol{A}_{12}$ are given by

$$[\boldsymbol{A}_{12}]_{1,1} = -\frac{h_1^3}{45} \text{ and } [\boldsymbol{A}_{12}]_{K,K} = -\frac{h_{K-1}^3}{45}.$$

Similarly, the off-diagonal elements $[\boldsymbol{A}_{12}]_{(i-1,i)}$ and $[\boldsymbol{A}_{12}]_{(i,i-1)}$, where $i = 2, \dots K$, where $i = 2, \dots K$ can be obtained as

$$\begin{aligned}
[\boldsymbol{A}_{12}]_{(i,i-1)} &= \int_{t_1}^{t_K} d_i(t)e_{i-1}(t)dt = \int_{t_{i-1}}^{t_i} a_{i-1}^{+}(t)c_{i-1}^{-}(t)dt + \\
&= \int_{t_{i-1}}^{t_i} \left(\frac{t-t_{i-1}}{h_{i-1}}\right)\frac{1}{6}\left[\frac{(t_i-t)^3}{h_{i-1}} - h_{i-1}(t_i-t)\right]dt \\
&= \int_{t_{i-1}}^{t_i} \frac{(t-t_{i-1})(t_i-t)^3}{6h_{i-1}^2}dt - \int_{t_{i-1}}^{t_i} \frac{(t-t_{i-1})(t_i-t)}{6}dt \\
&= \frac{h_{i-1}^3}{120} - \frac{h_{i-1}^3}{36} = -\frac{7}{360}h_{i-1}^3
\end{aligned}$$

$$\begin{aligned}
[\boldsymbol{A}_{12}]_{(i-1,i)} &= \int_{t_1}^{t_K} d_{i-1}(t)e_i(t)dt = \int_{t_{i-1}}^{t_i} a_{i-1}^{-}(t)c_{i-1}^{+}(t)dt + \\
&= \int_{t_{i-1}}^{t_i} \left(\frac{t_i-t}{h_{i-1}}\right)\frac{1}{6}\left[\frac{(t-t_{i-1})^3}{h_{i-1}} - h_{i-1}(t-t_{i-1})\right]dt \\
&= \int_{t_{i-1}}^{t_i} \frac{(t-t_{i-1})^3(t_i-t)}{6h_{i-1}^2}dt - \int_{t_{i-1}}^{t_i} \frac{(t-t_{i-1})(t_i-t)}{6}dt \\
&= \frac{h_{i-1}^3}{120} - \frac{h_{i-1}^3}{36} = -\frac{7}{360}h_{i-1}^3
\end{aligned}$$

Calculating $\boldsymbol{A}_{22}$ The ith leading diagonal element of $\boldsymbol{A}_{22}$, for $i = 2, \ldots K-1$, is given by

$$
\begin{aligned}
[\boldsymbol{A}_{22}]_{i,i} = \int_{t_1}^{t_K} e_i(t)^2 dt &= \int_{t_{i-1}}^{t_i} c_{i-1}^{+}(t)^2 dt + \int_{t_i}^{t_{i+1}} c_i^{-}(t)^2 dt \\
&= \int_{t_{i-1}}^{t_i} \left\{ \frac{1}{6} \left[\frac{(t-t_{i-1})^3}{h_{i-1}} - h_{i-1}(t-t_{i-1}) \right] \right\}^2 dt \\
&\quad + \int_{t_i}^{t_{i+1}} \left\{ \frac{1}{6} \left[\frac{(t_{i+1}-t)^3}{h_i} - h_i(t_{i+1}-t) \right] \right\}^2 dt \\
&= \frac{4}{315} \left(h_{i-1}^5 + h_i^5 \right)
\end{aligned}
$$

The first and last leading diagonal elements of $\boldsymbol{A}_{12}$ are given by

$$
[\boldsymbol{A}_{12}]_{1,1} = \frac{4}{315} h_1^5 \text{ and } [\boldsymbol{A}_{12}]_{K,K} = \frac{4}{315} h_{K-1}^5.
$$

Similarly, the off-diagonal elements $[\boldsymbol{A}_{22}]_{(i-1,i)}$ and $[\boldsymbol{A}_{22}]_{(i,i-1)}$, where $i = 2, \ldots K$, where $i = 2, \ldots K$ can be obtained as

$$
\begin{aligned}
[\boldsymbol{A}_{22}]_{(i,i-1)} &= [\boldsymbol{A}_{22}]_{(i-1,i)} = \int_{t_1}^{t_K} e_i(t) e_{i-1}(t) dt \\
&= \int_{t_{i-1}}^{t_i} c_{i-1}^{+}(t) c_{i-1}^{-}(t) dt + \\
&= \int_{t_{i-1}}^{t_i} \frac{1}{6} \left[\frac{(t-t_{i-1})^3}{h_{i-1}} - h_{i-1}(t-t_{i-1}) \right] \frac{1}{6} \left[\frac{(t_i-t)^3}{h_{i-1}} - h_{i-1}(t_i-t) \right] dt \\
&= \frac{31}{15120} h_{i-1}^5
\end{aligned}
$$

Re-express the L2-norm of $\beta(t)$ Using the relation between $\boldsymbol{\theta}$ and $\boldsymbol{\delta}$, $\boldsymbol{\delta} = \boldsymbol{F}\boldsymbol{\theta}$, we can re-express the L2-norm in (A.6) as

$$
\begin{aligned}
\int_{t_1}^{t_K} (\beta(t))^2 dt &= (\boldsymbol{\theta}^T, \boldsymbol{\theta}^T \boldsymbol{F}^T) \begin{bmatrix} \boldsymbol{A}_{11} & \boldsymbol{A}_{12} \\ \boldsymbol{A}_{12}^T & \boldsymbol{A}_{22} \end{bmatrix} (\boldsymbol{\theta}, \boldsymbol{F}\boldsymbol{\theta}) \\
&= \boldsymbol{\theta}^T \left(\boldsymbol{A}_{11} + \boldsymbol{F}^T \boldsymbol{A_{12}}^T + \boldsymbol{A}_{12} \boldsymbol{F} + \boldsymbol{F}^T \boldsymbol{A}_{22} \boldsymbol{F} \right) \boldsymbol{\theta}.
\end{aligned}
$$

Therefore, the sparsity-penalty matrix $\boldsymbol{\Omega^{(1)}}$ is given by $\boldsymbol{A}_{11} + \boldsymbol{F}^T \boldsymbol{A_{12}}^T + \boldsymbol{A}_{12}\boldsymbol{F} + \boldsymbol{F}^T \boldsymbol{A}_{22} \boldsymbol{F}$. This completes the proof. □

A.3 Natural cubic spline and its smoothness-penalty matrix $\mathbf{\Omega^{(2)}}$

As derived in (Wood, 2017, Section 5.3.1), the unscaled smoothness-penalty matrix takes the form

$$\mathbf{\Omega^{(2)}}/M^2 = \boldsymbol{D^T B^{-1} D},$$

which can be readily extracted using `smooth.construct.cr()` from `mgcv` package.

B Derive $\lambda_{\max}$

B.1 Subgradient of $h(\boldsymbol{\theta}) = \sqrt{\boldsymbol{\theta}^T \boldsymbol{H\theta}}$, $h : \mathbb{R}^K \to \mathbb{R}$

When $\boldsymbol{\theta} \neq \mathbf{0}$, the subgradient of $h(\boldsymbol{\theta})$ coincides with its gradient, and we have

$$\partial h(\boldsymbol{\theta}) = \frac{\boldsymbol{H\theta}}{\sqrt{\boldsymbol{\theta}^T \boldsymbol{H\theta}}}, \quad \text{for } \boldsymbol{\theta} \neq \mathbf{0}.$$

By the definition, a subgradient of $h(\cdot)$ at $\mathbf{0}$ is any $\boldsymbol{g} \in \mathbb{R}^K$ such that,

$$h(\boldsymbol{\theta}) \geq h(\mathbf{0}) + \boldsymbol{g^T\theta},\ \forall \boldsymbol{\theta} \in \mathbb{R}^K,$$

which implies

$$\sqrt{\boldsymbol{\theta}^T \boldsymbol{H\theta}} \geq \boldsymbol{g^T\theta}\ \forall \boldsymbol{\theta} \in \mathbb{R}^K.$$

Write the Cholesky decomposition of positive semidefinite matrix $\boldsymbol{H}$ as $\boldsymbol{H} = \boldsymbol{L}^T\boldsymbol{L}$, where $\boldsymbol{L}$ is an upper triangular matrix with positive diagonal entries. Define $\boldsymbol{x} = \boldsymbol{L\theta} \in \mathbb{R}^K$, then we have

$$\boldsymbol{\theta}^T \boldsymbol{H\theta} = \boldsymbol{\theta}^T \boldsymbol{L^T L\theta} = \boldsymbol{x^T x} = \|\boldsymbol{x}\|_2^2.$$

Thus, the set of subgradients $\partial h(\mathbf{0})$ can be rewritten as

$$\left\{\boldsymbol{g} \in \mathbb{R}^K : \frac{\boldsymbol{g^T\theta}}{\|\boldsymbol{x}\|_2} \leq 1\ \forall \boldsymbol{\theta} \in \mathbb{R}^K.\right\}.$$

We can also rewrite the inner product $\boldsymbol{g^T\theta}$ in terms of $\boldsymbol{x}$,

$$\boldsymbol{g^T\theta} = \boldsymbol{g^T L}^{-1}\boldsymbol{x} = \left[\left(\boldsymbol{L}^{-1}\right)^T \boldsymbol{g}\right]^T \boldsymbol{x}.$$

By the Cauchy-Schwarz inequality, we have

$$\left[\left(\boldsymbol{L}^{-1}\right)^T \boldsymbol{g}\right]^T \boldsymbol{x} \leq \|\left(\boldsymbol{L}^{-1}\right)^T \boldsymbol{g}\|_2 \|\boldsymbol{x}\|_2 = \sqrt{\boldsymbol{g^T L^{-1}}\left(\boldsymbol{L}^{-1}\right)^T \boldsymbol{g}} \cdot \|\boldsymbol{x}\|_2 = \sqrt{\boldsymbol{g^T H^{-1} g}} \cdot \|\boldsymbol{x}\|_2,$$

for $\forall \boldsymbol{x} \in \mathbb{R}^K$. In other words, we have derived the upper bound of $\dfrac{\boldsymbol{g^T\theta}}{\sqrt{\boldsymbol{\theta}^T \boldsymbol{H\theta}}}$ for any $\boldsymbol{\theta}$ as $\sqrt{\boldsymbol{g^T H^{-1} g}}$. The subgradient of $h(\boldsymbol{\theta})$ at $\mathbf{0}$ is thus the set

$$\partial h(\mathbf{0}) = \left\{\boldsymbol{g} \in \mathbb{R}^K : \sqrt{\boldsymbol{g^T H^{-1} g}} \leq 1\right\}.$$

B.2 Derive $\lambda_{\max}$

We aim to solve the following optimization problem

$$\widehat{\boldsymbol{\theta}} = \arg\min_{\boldsymbol{\theta}} \left\{ \ell(\boldsymbol{\theta}) + \lambda \sum_{p=1}^{P} \sqrt{\boldsymbol{\theta}_p^T \boldsymbol{H}_\alpha \boldsymbol{\theta}_p} \right\}, \tag{A.7}$$

where $\ell(\boldsymbol{\theta})$ is the twice negative log likelihood, expressed as

$$\ell(\boldsymbol{\theta}) = -2 \sum_{i=1}^{N} \sum_{j=1}^{m_i} \left\{ S_{ij} \log(\pi_{ij}) + (X_{ij} - S_{ij}) \log(1 - \pi_{ij}) \right\}. \tag{A.8}$$

For a given value of α, we can derive the smallest λ that gives the entire effect vector $\widehat{\boldsymbol{\theta}}_1 = \ldots = \widehat{\boldsymbol{\theta}}_P = \mathbf{0}$ in our optimization problem (A.7). This value is referred to as $\lambda_{\max}$ in the regularization path for λ.

The derivation of $\lambda_{\max}$ involves calculating the optimality conditions for the nonlinear programming problem in (A.7), specifically the Karush-Kuhn-Tucker (KKT) conditions. These conditions determine whether a solution is optimal by checking if $\mathbf{0}$ is a subgradient of our objective function

$$f(\boldsymbol{\theta}) = \ell(\boldsymbol{\theta}) + \lambda \sum_{p=1}^{P} \sqrt{\boldsymbol{\theta}_p^T \mathbf{H}_\alpha \boldsymbol{\theta}_p}$$

at $\boldsymbol{\theta}^\star$. For the differential part $\ell(\boldsymbol{\theta})$ of $f(\boldsymbol{\theta})$, the gradient is given by

$$\nabla \ell(\boldsymbol{\theta}) = -2 \left[\mathbb{X}^T (\boldsymbol{S} - \boldsymbol{\Lambda}_{\boldsymbol{X}} \boldsymbol{\pi}) \right],$$

where $\boldsymbol{S} \in \mathbb{R}^M$ is the vector concatenating S_{ij}, and $\boldsymbol{\Lambda}_{\boldsymbol{X}} \in \mathbb{R}^{M \times M}$ is the diagonal matrix with read-depth values X_{ij}.

Based on the results derived in B.1, we have that the subgradient for the non-differential part $h(\boldsymbol{\theta}_p) = \sqrt{\boldsymbol{\theta}_p^T \boldsymbol{H}_\alpha \boldsymbol{\theta}_p}$ of our objective function is

$$\partial h(\boldsymbol{\theta}_p) = \begin{cases} \dfrac{\boldsymbol{H}_\alpha \boldsymbol{\theta}_p}{\sqrt{\boldsymbol{\theta}_p^T \boldsymbol{H}_\alpha \boldsymbol{\theta}_p}}, & \boldsymbol{\theta}_p \neq \mathbf{0}, \\ \left\{ \boldsymbol{g} \in \mathbb{R}^K : \sqrt{\boldsymbol{g}^T \boldsymbol{H}_\alpha^{-1} \boldsymbol{g}} \leq 1 \right\}, & \boldsymbol{\theta}_p = \mathbf{0}; \end{cases} \tag{A.9}$$

The KKT conditions for $\boldsymbol{\theta}$ to be optimal in our nonlinear programming problem are

$$\begin{cases} \boldsymbol{a}_p = \lambda \dfrac{\boldsymbol{H}_\alpha \boldsymbol{\theta}_p}{\sqrt{\boldsymbol{\theta}_p^T \boldsymbol{H}_\alpha \boldsymbol{\theta}_p}}, & \text{if } \boldsymbol{\theta}_p \neq \mathbf{0}, \\ \sqrt{\boldsymbol{a}_p^T \boldsymbol{H}_\alpha^{-1} \boldsymbol{a}_p} \leq \lambda, & \text{if } \boldsymbol{\theta}_p = \mathbf{0}, \end{cases} \qquad p = 1, 2, \ldots P, \tag{A.10}$$

where $\boldsymbol{a}_p = 2\left[\mathbb{X}_p^T(\boldsymbol{S} - \boldsymbol{\Lambda}_{\boldsymbol{X}}\boldsymbol{\pi})\right] \in \mathbb{R}^K$ denotes the sub-vector of $-\nabla\ell(\boldsymbol{\theta})$ corresponding to $\boldsymbol{\theta}_p$. Considering the case when the optimal minimizer for (A.7) has components $\boldsymbol{\theta}_1 = \ldots = \boldsymbol{\theta}_p = \boldsymbol{0}$, the KKT conditions imply:

$$\lambda \geq \sqrt{\boldsymbol{b}_p^T \boldsymbol{H}_\alpha^{-1} \boldsymbol{b}_p}, \text{ for } \forall p \in \{1, 2, \ldots, P\}.$$

Here, $\boldsymbol{b}_p = 2\left[\mathbb{X}_p^T(\boldsymbol{S} - \boldsymbol{\Lambda}_{\boldsymbol{X}}\boldsymbol{\pi}_0)\right]$ is the sub-vector of $-\nabla\ell(\boldsymbol{\theta})$ corresponding to $\boldsymbol{\theta}_p$ evaluating from an intercept-only model, where $\boldsymbol{\pi}_0 \in \mathbb{R}^M$ has elements $[1+\exp(-\beta_0(t_{ij}))]^{-1}$. Therefore, we establish that the smallest λ giving $\boldsymbol{\theta}_1 = \boldsymbol{\theta}_2 = \ldots = \boldsymbol{\theta}_p = \boldsymbol{0}$ is

$$\lambda_{\max} = \max_{p \in \{1,2,\ldots,P\}} \left\{ \sqrt{\boldsymbol{b}_p^T \boldsymbol{H}_\alpha^{-1} \boldsymbol{b}_p} \right\}.$$

Algorithm S 1: Proximal gradient algorithm with backtracking line search.

Leftleft Initialize $\boldsymbol{\eta}^{(0)} = \boldsymbol{0}$; Choose some $0 < \delta < 1$; Choose $\varepsilon = 10^{-6}$; Set $s = 0$;
repeat
  $s \leftarrow s + 1$;
  Initialize $t = t_{\text{init}}$;
  repeat
    $t \leftarrow \delta t$;
  until
  $\ell(\boldsymbol{\eta}^{(s-1)} - tG_t(\boldsymbol{\eta}^{(s-1)})) \leq \ell(\boldsymbol{\eta}^{(s-1)}) - t\nabla\ell(\boldsymbol{\eta}^{(s-1)})^T G_t(\boldsymbol{\eta}^{(s-1)}) + \frac{t}{2}\|G_t(\boldsymbol{\eta}^{(s-1)})\|_2^2$;
  Set $t_s = t$;
  Update $\boldsymbol{\eta}^{(s)} \longleftarrow \text{prox}_{t_s}\left[\boldsymbol{\eta}^{(s-1)} - t_s\nabla\ell(\boldsymbol{\eta}^{(s-1)})\right]$ as defined in (**??**);
until $\|\boldsymbol{\eta}^{(s)} - \boldsymbol{\eta}^{(s-1)}\|_2 < \varepsilon$;
Return $\boldsymbol{\eta}^{(s)}$;

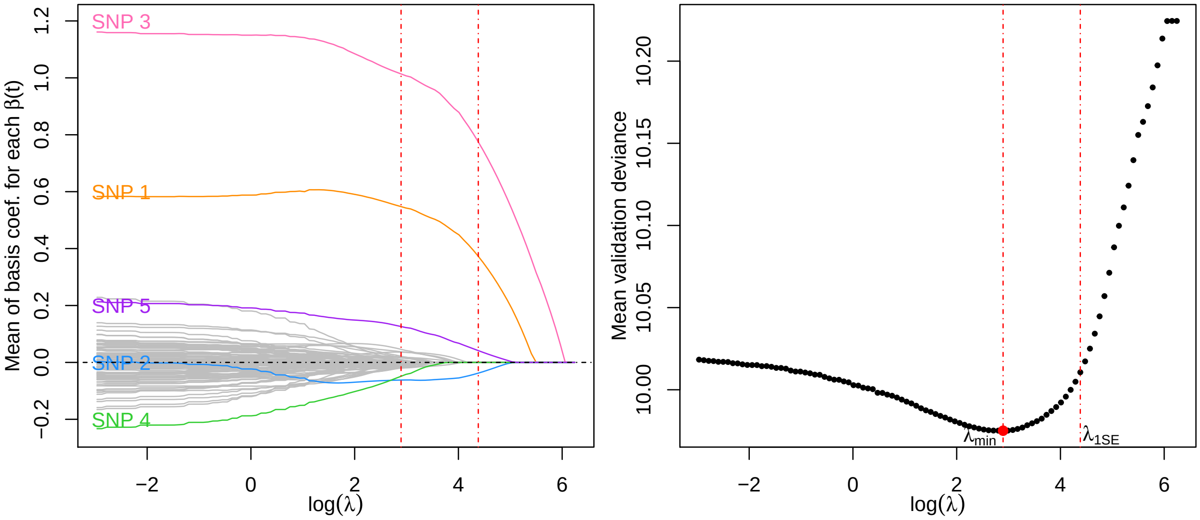

Figure S1. Regularization path for the mean of basis coefficients of each $\beta(t)$ (left), and the mean validation deviance (right), across 100 different values of λ. The optimal values of λ, denoted as λ_{min} and λ_{1SE}, were chosen based on the minimum validation deviance and the 1-SE rule, respectively. The data used in this plot were simulated from Example 1, with $P = 100$ and $P_{\text{true}} = 5$. The true mQTLs, labeled as SNP 1-5, are indicated in the plot. The value of α was set to its best choice, $\alpha = 0.95$.

C Additional simulation design

We first simulated the minor allele frequencies for each candidate SNP independently from a uniform distribution, i.e. $f_p \sim \text{Uniform}(0.1, 0.5)$, for $p = 1, 2, \ldots, P$. We then generated genotype Z_p from the truncated multivariate normal distribution with correlation matrix $\mathbf{\Sigma} \in \mathcal{R}^{P\times P}$ and appropriate thresholding for the mean such that

$$P(Z_p = 0) = (1 - f_p)^2, P(Z_p = 2) = f_p^2, \text{ and } P(Z_p = 1) = 2f_p(1 - f_p).$$

We specified $\mathbf{\Sigma}$ as a block diagonal matrix, consisting of sub-matrices $\mathbf{\Sigma}^{\text{sub}} \in \mathcal{R}^{20\times 20}$ of the form $\mathbf{\Sigma}^{\text{sub}} = (1-\rho)\boldsymbol{I} + \rho\mathbf{1}$, where $\boldsymbol{I} \in \mathcal{R}^{20\times 20}$ is an identify matrix, $\mathbf{1} \in \mathcal{R}^{20\times 20}$ is a matrix with all elements as 1. Here ρ is the correlation coefficient and we explored the settings $\rho = 0, 0.3$ or 0.7, corresponding to no, moderate and strong dependence among SNPs. To simulate realistic read depths X_{ij}, we first extracted a spatially correlated read-depth pattern from the real data, denoted as $f^X(t)$, by fitting a cubic spline to the median read-depth across positions. We then generated the read depth X_{ij} by adding Bernoulli random variables (with proportion 0.5) to $f^X(t)$. Given the values of $\{\boldsymbol{Z}, \boldsymbol{X}\}$ and $\{\beta_p(t), p = 0, 1, \ldots, P\}$ under each example and setting, we simulated the methylated counts Y_{ij} from the model in Equation 1 in the main manuscript. In addition, an independent test set of the same size was generated for model validation purposes. A total of $R = 100$ simulation runs were used.

Table S3. The shapes of the nonzero $\beta_p(t)$s associated with covariates Z_1 to Z_5 in our four simulation examples. $\beta_p(t) = 0$ for all remaining covariates except for the illustrated ones.

Example 1: (smooth, $N = 50$, $P_{true} = 5$, $P = 100$ or 1000)

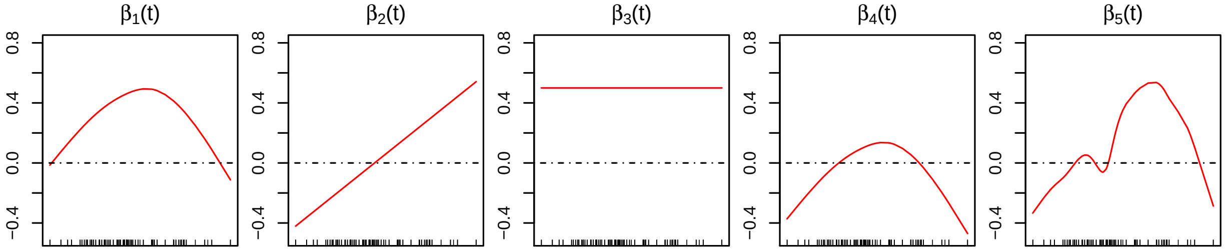

Example 2: (nonsmooth, $N = 50$, $P_{true} = 5$, $P = 100$ or 1000)

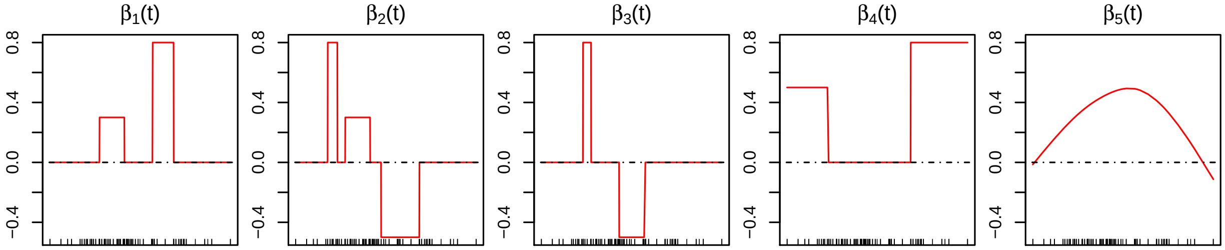

Example 3: (smooth, $N = 20$, $P_{true} = 5$, $P = 50, 100, 150, 200$ or 1000);
Similar to Example 1 but with smaller effect sizes for Z_1, Z_3 and Z_5 and smaller sample sizes.

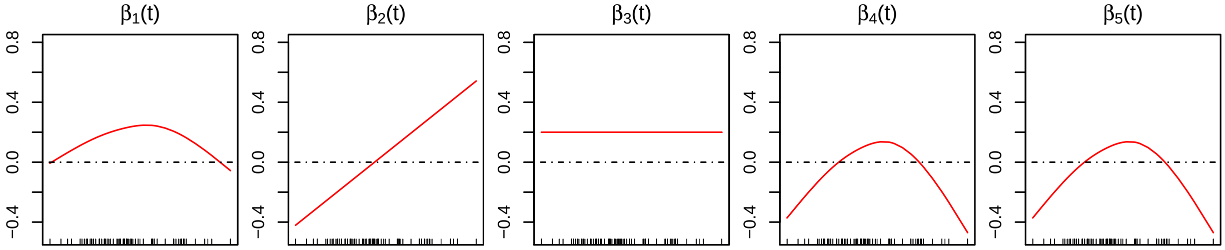

Example 4: (smooth, $N = 20$, $P_{true} = 10$, $P = 50, 100, 150, 200$ or 1000)
The same $\beta_p(t)$ for $p = 1, \ldots, 5$ as Example 3 and $\beta_p(t) = \beta_{p-5}(t)$ for $p = 6, 7, \ldots, 10$.

Table S4. A catalogue of all the simulation results

Evaluation themes	Examples & Settings	Results
Methods: SSP, SSP0, gLASSO, and GAM		
Estimation	Example 1 $(P = 100, \rho = 0)$	Table 2 ; Figures S2-S5
	Example 1 $(P = 100, \rho = 0.3)$	Table S5
	Example 1 $(P = 100, \rho = 0.7)$	Table S6
	Example 1 $(P = 1000)$	Table S15
	Example 2	Table S14;Figures S6-S9
	Examples 3 and 4	Table S17; Table S18; Table S19
Prediction	Examples 1 and 2	Table 3; Table S9
	Examples 3 and 4	Table S16
Selection	Examples 1 and 2	Table 4
	Examples 3 and 4	Table 5
Methods: ^{a}SSP, ^{a}SSP0, ^{a}gLASSO — the adaptive versions		
Estimation	Example 1 $(P = 100, \rho = 0)$	Table S7
Prediction	Example 1 $(P = 100, \rho = 0)$	Table S10
Selection	Example 1 $(P = 100, \rho = 0)$	Table S12
Methods: SSP^{1SE}, SSP0^{1SE}, gLASSO^{1SE} — with the 1-SE-rule		
Estimation	Example 1 $(P = 100, \rho = 0)$	Table S8
Prediction	Example 1 $(P = 100, \rho = 0)$	Table S11
Selection	Example 1 $(P = 100, \rho = 0)$	Table S13

Table S5. Integrated Squared Bias (IBIAS^2), Integrated Variance (IVAR) and Integrated Mean Square Error (IMSE) of the first 10 varying coefficients of **Example 1** $(P = 100, \rho = 0.3)$, using SSP, SSP0, group LASSO and GAM.

	IBIAS^2				IVAR				IMSE			
	SSP	SSP0	gLASSO	GAM	SSP	SSP0	gLASSO	GAM	SSP	SSP0	gLASSO	GAM
$\beta_1(t)$	0.223	0.421	0.554	3.245	0.243	0.374	0.460	5.150	0.466	0.795	1.014	8.395
$\beta_2(t)$	0.744	1.280	1.264	1.425	0.264	0.318	0.320	3.561	1.008	1.597	1.584	4.986
$\beta_3(t)$	0.532	0.987	0.980	7.943	0.273	0.410	0.449	9.100	0.805	1.396	1.429	17.043
$\beta_4(t)$	0.679	0.905	0.977	0.459	0.176	0.203	0.210	2.745	0.856	1.108	1.187	3.205
$\beta_5(t)$	0.900	0.798	0.882	0.344	0.354	0.422	0.428	3.491	1.254	1.220	1.310	3.835
$\beta_6(t)$	1.0e-03	1.3e-03	1.4e-03	1.2e-02	1.5e-02	1.6e-02	1.8e-02	1.654	1.6e-02	1.8e-02	1.9e-02	1.665
$\beta_7(t)$	9.3e-04	1.0e-03	9.6e-04	4.3e-02	9.4e-03	1.2e-02	1.1e-02	2.646	1.0e-02	1.3e-02	1.2e-02	2.689
$\beta_8(t)$	1.7e-03	1.7e-03	1.5e-03	2.8e-02	1.4e-02	1.7e-02	1.8e-02	1.851	1.6e-02	1.8e-02	1.9e-02	1.879
$\beta_9(t)$	3.7e-04	5.0e-04	6.2e-04	2.2e-02	8.0e-03	1.1e-02	1.1e-02	2.611	8.3e-03	1.2e-02	1.1e-02	2.633
$\beta_{10}(t)$	1.4e-03	1.6e-03	1.4e-03	2.0e-02	1.6e-02	1.8e-02	1.8e-02	3.177	1.7e-02	1.9e-02	1.9e-02	3.197
${}^{\dagger}\sum_1^{100}$	3.097	4.412	4.676	16.374	2.031	2.536	2.600	251.761	5.128	6.948	7.276	268.135

†: sum of the estimation measures across the 100 varying coefficients

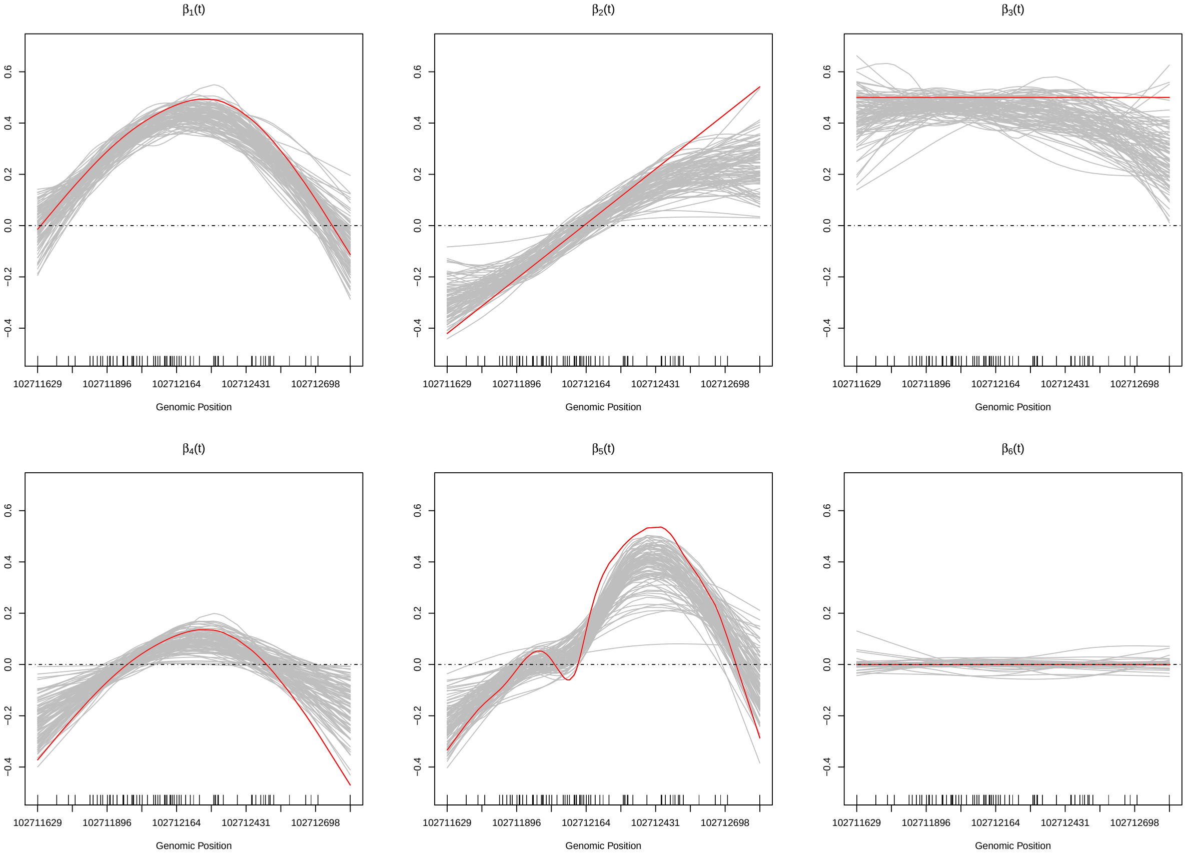

Figure S2. **SSP** estimates of the first 6 varying coefficients (gray) in Example 1 ($P = 100, \rho = 0$) over 100 simulation runs. The red curves are the truth.

Table S6. Integrated Squared Bias (IBIAS^2), Integrated Variance (IVAR) and Integrated Mean Square Error (IMSE) of the first 10 varying coefficients of **Example 1** ($P = 100, \rho = 0.7$), using SSP, SSP0, group LASSO and GAM.

	IBIAS^2				IVAR				IMSE			
	SSP	SSP0	gLASSO	GAM	SSP	SSP0	gLASSO	GAM	SSP	SSP0	gLASSO	GAM
$\beta_1(t)$	0.258	0.489	0.691	4.223	0.403	0.625	0.756	6.143	0.661	1.115	1.447	10.367
$\beta_2(t)$	0.916	1.521	1.528	1.192	0.305	0.372	0.383	4.842	1.221	1.894	1.911	6.034
$\beta_3(t)$	0.710	1.269	1.317	7.269	0.347	0.529	0.572	11.026	1.056	1.799	1.890	18.295
$\beta_4(t)$	0.917	1.109	1.108	0.755	0.204	0.223	0.231	4.967	1.121	1.332	1.340	5.722
$\beta_5(t)$	1.177	0.968	1.137	0.333	0.548	0.632	0.631	4.914	1.725	1.601	1.768	5.248
$\beta_6(t)$	3.6e-03	4.7e-03	4.2e-03	1.1e-01	1.9e-02	2.5e-02	2.5e-02	4.389	2.3e-02	2.9e-02	2.9e-02	4.497
$\beta_7(t)$	2.5e-03	3.1e-03	2.8e-03	2.8e-02	1.5e-02	2.1e-02	2.0e-02	4.244	1.7e-02	2.4e-02	2.3e-02	4.273
$\beta_8(t)$	4.3e-03	5.1e-03	3.9e-03	9.5e-02	2.5e-02	3.2e-02	2.6e-02	2.102	2.9e-02	3.7e-02	3.0e-02	2.197
$\beta_9(t)$	1.2e-03	2.4e-03	2.8e-03	1.7e-01	1.7e-02	2.3e-02	2.0e-02	3.504	1.9e-02	2.5e-02	2.3e-02	3.674
$\beta_{10}(t)$	3.1e-03	4.1e-03	3.9e-03	7.0e-02	2.1e-02	2.5e-02	2.3e-02	2.432	2.4e-02	2.9e-02	2.7e-02	2.501
$^\dagger\sum_1^{100}$	4.022	5.415	5.840	17.318	2.661	3.342	3.438	359.066	6.684	8.757	9.278	376.385

†: sum of the estimation measures across the 100 varying coefficients

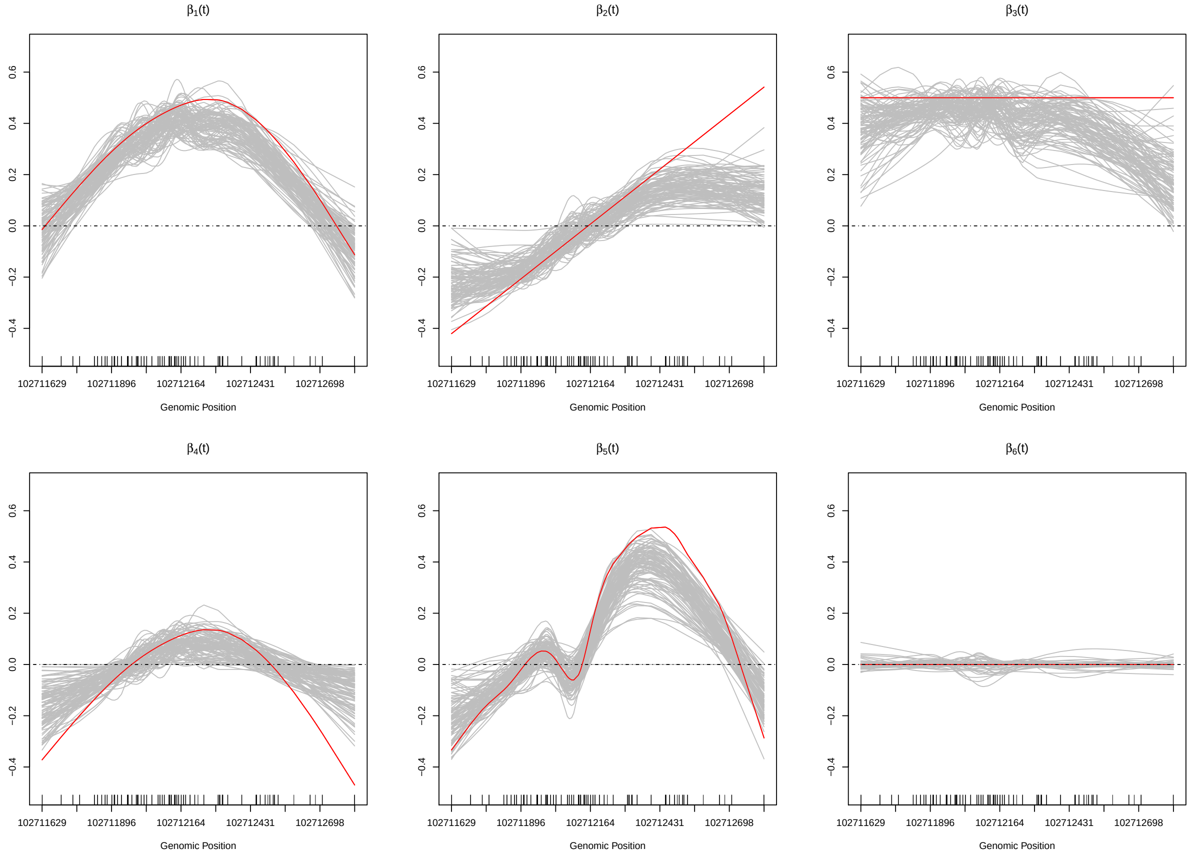

Figure S3. **SSP0** estimates of the first 6 varying coefficients (gray) in Example 1 ($P = 100, \rho = 0$) over 100 simulation runs. The red curves are the truth.

Table S7. Integrated Squared Bias (IBIAS^2), Integrated Variance (IVAR) and Integrated Mean Square Error (IMSE) of the first 10 varying coefficients of Example 1 ($P = 100, \rho = 0$), using the **adaptive** SSP, SSP0, and group LASSO.

	IBIAS^2				IVAR				IMSE			
	^{a}SSP	^{a}SSP0	^{a}SSP-fix$\alpha^\ddagger$	^{a}gLASSO	^{a}SSP	^{a}SSP0	^{a}SSP-fixα	^{a}gLASSO	^{a}SSP	^{a}SSP0	^{a}SSP-fixα	^{a}gLASSO
$\beta_1(t)$	0.022	0.033	0.023	0.029	0.248	0.371	0.239	0.412	0.270	0.404	0.262	0.442
$\beta_2(t)$	0.090	0.264	0.078	0.377	0.245	0.358	0.226	0.355	0.335	0.623	0.304	0.732
$\beta_3(t)$	0.037	0.085	0.035	0.080	0.299	0.453	0.283	0.486	0.336	0.539	0.318	0.566
$\beta_4(t)$	0.161	0.297	0.165	0.467	0.246	0.325	0.247	0.323	0.407	0.622	0.412	0.790
$\beta_5(t)$	0.214	0.126	0.257	0.180	0.341	0.384	0.377	0.439	0.555	0.510	0.634	0.619
$\beta_6(t)$	1.1e-04	1.6e-04	1.1e-04	1.4e-04	1.6e-02	1.4e-02	1.6e-02	1.4e-02	1.7e-02	1.5e-02	1.6e-02	1.4e-02
$\beta_7(t)$	9.6e-05	9.5e-05	1.0e-04	3.6e-05	5.3e-03	3.8e-03	5.1e-03	5.0e-03	5.4e-03	3.9e-03	5.2e-03	5.0e-03
$\beta_8(t)$	8.3e-05	1.4e-04	9.4e-05	1.5e-04	1.0e-02	1.0e-02	1.1e-02	9.9e-03	1.1e-02	1.0e-02	1.1e-02	1.0e-02
$\beta_9(t)$	4.9e-05	3.9e-05	4.6e-05	1.1e-04	8.0e-03	5.1e-03	7.5e-03	8.4e-03	8.1e-03	5.2e-03	7.5e-03	8.5e-03
$\beta_{10}(t)$	7.9e-05	1.7e-05	9.7e-05	7.1e-05	8.4e-03	2.8e-03	8.7e-03	5.9e-03	8.5e-03	2.8e-03	8.8e-03	6.0e-03
$^\dagger \sum_1^{100}$	0.533	0.812	0.565	1.142	2.191	2.536	2.177	2.833	2.723	3.348	2.742	3.975

†: sum of the estimation measures across the 100 varying coefficients.
‡: adaptive SSP with fix α. Here we use the α selected by the ordinary SSP method and only tune the values of λ.

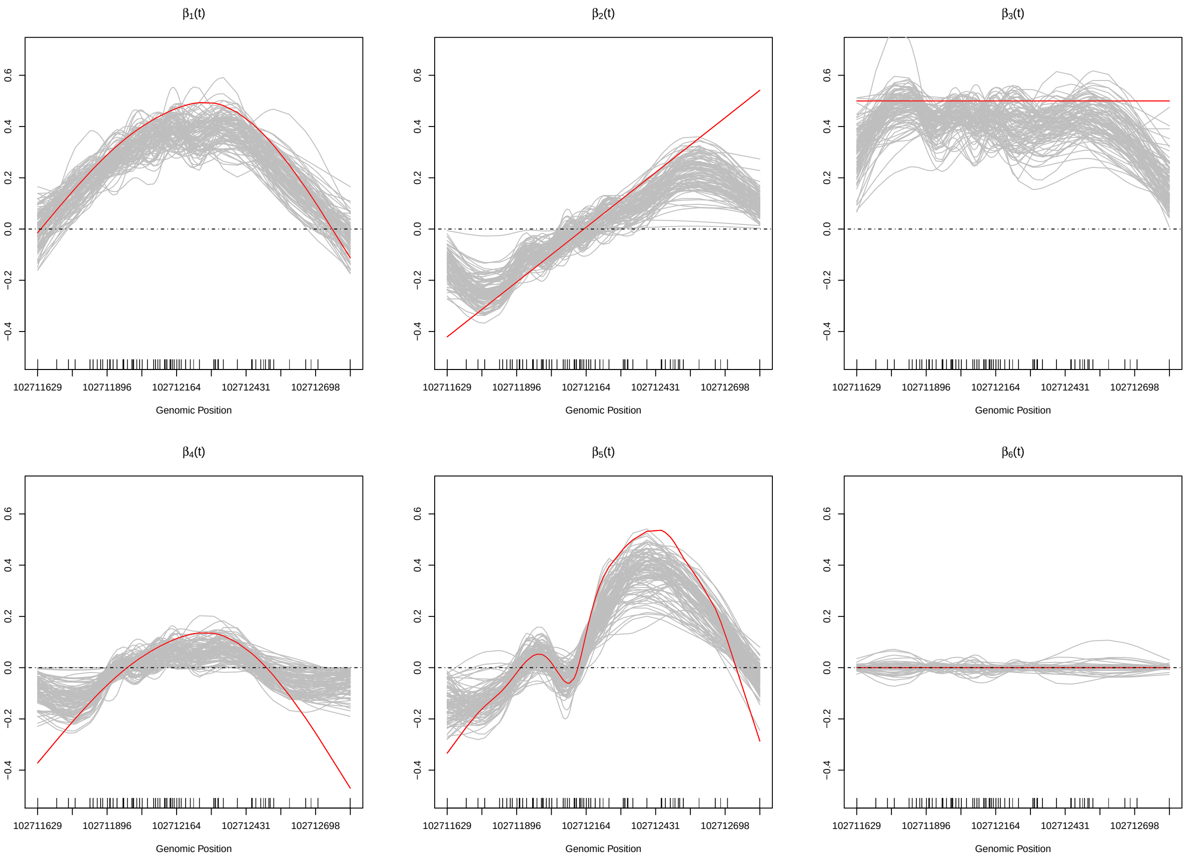

Figure S4. Group LASSO estimates of the first 6 varying coefficients (gray) in Example 1 ($P = 100, \rho = 0$) over 100 simulation runs. The red curves are the truth.

Table S8. Integrated Squared Bias (IBIAS^2), Integrated Variance (IVAR) and Integrated Mean Square Error (IMSE) of the first 10 varying coefficients of Example 1 ($P = 100, \rho = 0$), using **the 1 SE rule** for SSP, SSP0, and group LASSO.

	IBIAS2			IVAR			IMSE		
	SSP^{1SE}	SSP0^{1SE}	gLASSO^{1SE}	SSP^{1SE}	SSP0^{1SE}	gLASSO^{1SE}	SSP^{1SE}	SSP0^{1SE}	gLASSO^{1SE}
$\beta_1(t)$	1.142	1.593	1.872	0.195	0.278	0.348	1.337	1.871	2.220
$\beta_2(t)$	2.356	2.881	2.358	0.199	0.208	0.213	2.556	3.089	2.570
$\beta_3(t)$	2.198	3.019	2.829	0.374	0.512	0.575	2.571	3.531	3.404
$\beta_4(t)$	1.513	1.584	1.562	0.080	0.080	0.079	1.593	1.663	1.641
$\beta_5(t)$	3.186	2.879	2.656	0.390	0.419	0.423	3.576	3.298	3.079
$\beta_6(t)$	4.1e-07	6.4e-07	7.5e-06	4.5e-04	2.2e-04	7.4e-04	4.5e-04	2.2e-04	7.5e-04
$\beta_7(t)$	4.7e-07	5.9e-06	1.3e-06	4.7e-05	5.9e-04	1.3e-04	4.7e-05	5.9e-04	1.3e-04
$\beta_8(t)$	2.6e-08	1.0e-06	1.4e-06	5.1e-06	8.6e-05	1.6e-04	5.1e-06	8.7e-05	1.7e-04
$\beta_9(t)$	3.1e-11	4.0e-07	4.3e-08	3.1e-09	1.4e-04	4.3e-06	3.1e-09	1.4e-04	4.3e-06
$\beta_{10}(t)$	0.0e+00	4.3e-07	5.2e-07	0.0e+00	4.1e-05	2.2e-04	0.0e+00	4.2e-05	2.2e-04
$\sum_1^{100}$	10.396	11.956	11.276	1.265	1.532	1.678	11.661	13.489	12.954

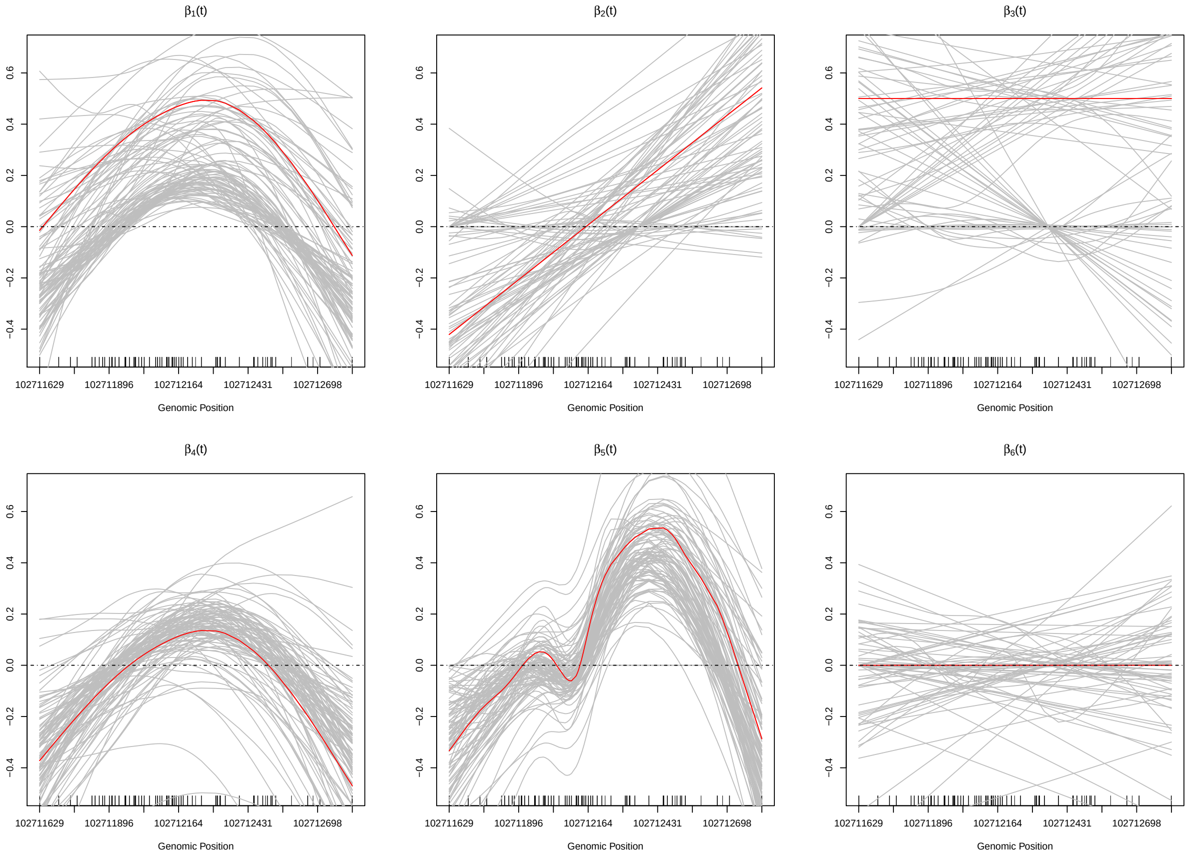

Figure S5. **GAM** estimates of the first 6 varying coefficients (gray) in Example 1 ($P = 100, \rho = 0$) over 100 simulation runs. The red curves are the truth.

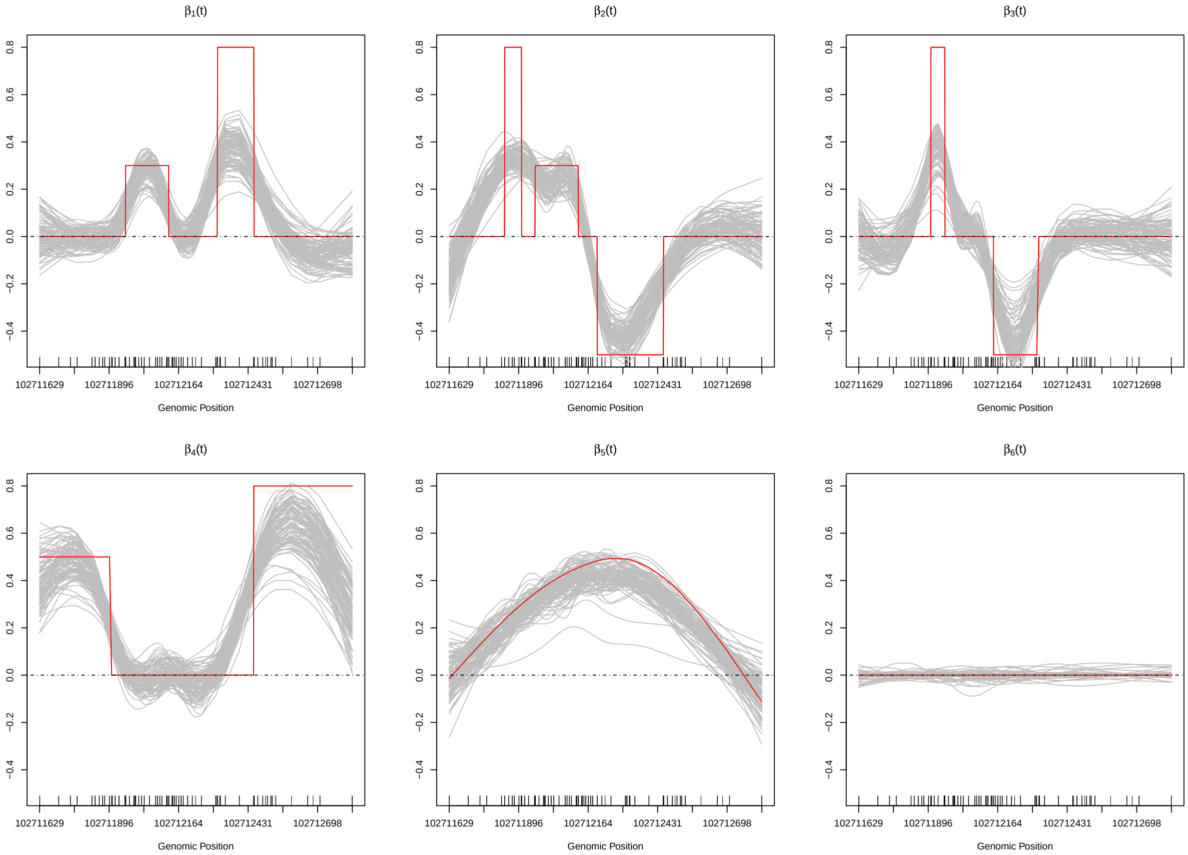

Figure S6. **SSP** estimates of the first 6 varying coefficients (gray) in Example 2 ($P = 100, \rho = 0$) over 100 simulation runs. The red curves are the truth.

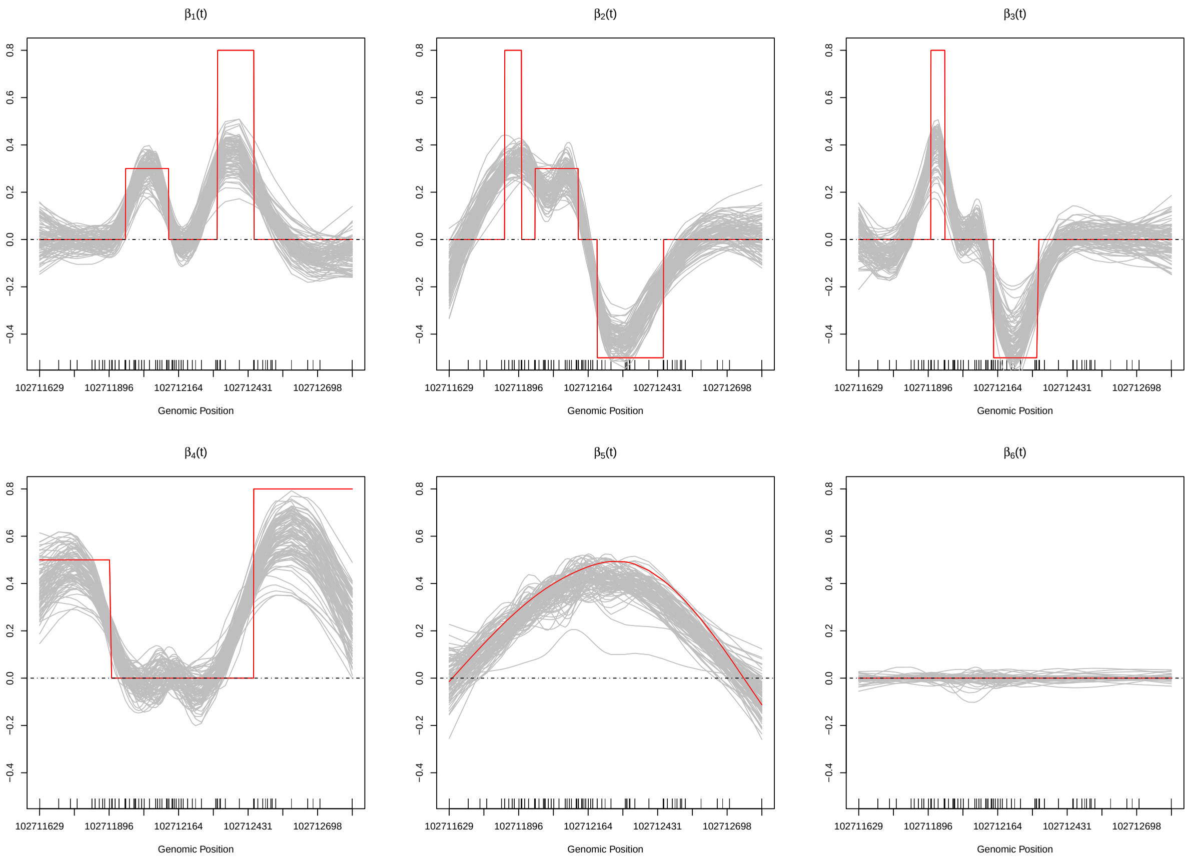

Figure S7. **SSP0** estimates of the first 6 varying coefficients (gray) in Example 2 ($P = 100, \rho = 0$) over 100 simulation runs. The red curves are the truth.

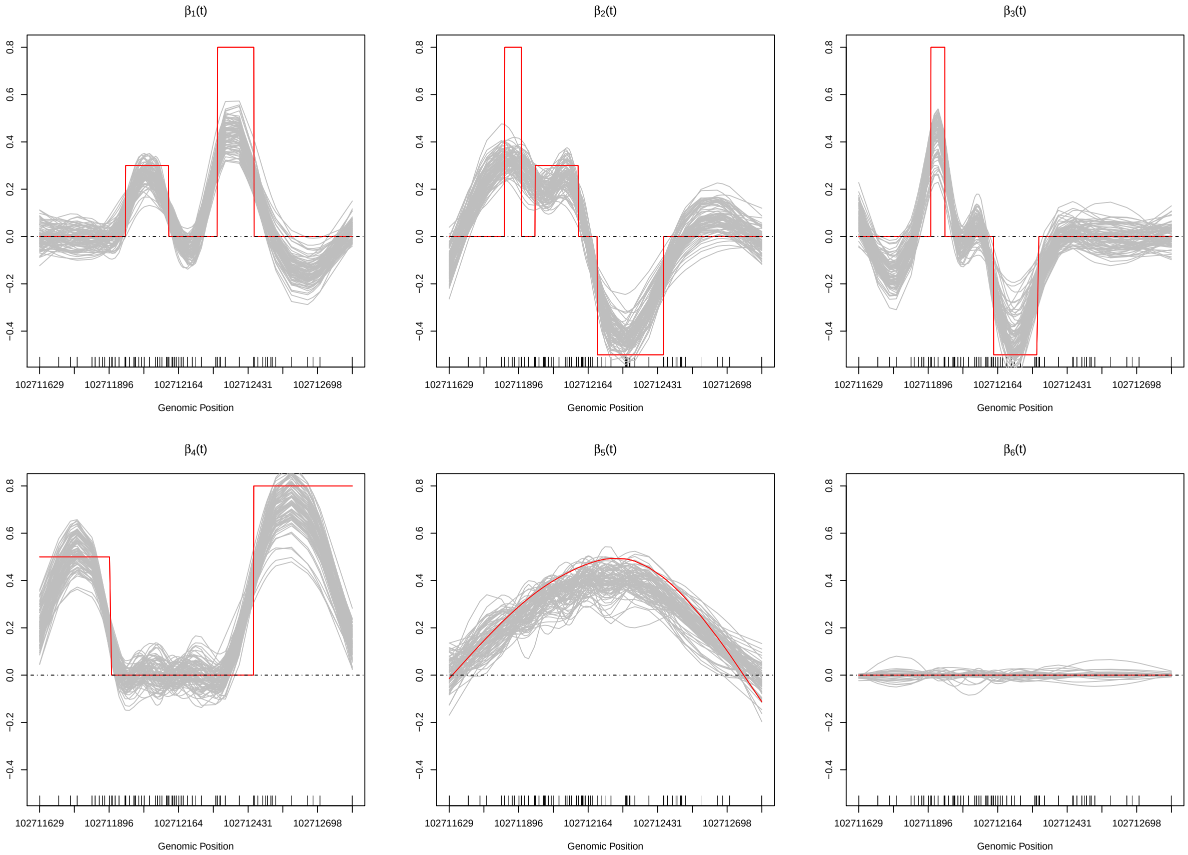

Figure S8. Group LASSO estimates of the first 6 varying coefficients (gray) in Example 2 ($P = 100, \rho = 0$) over 100 simulation runs. The red curves are the truth.

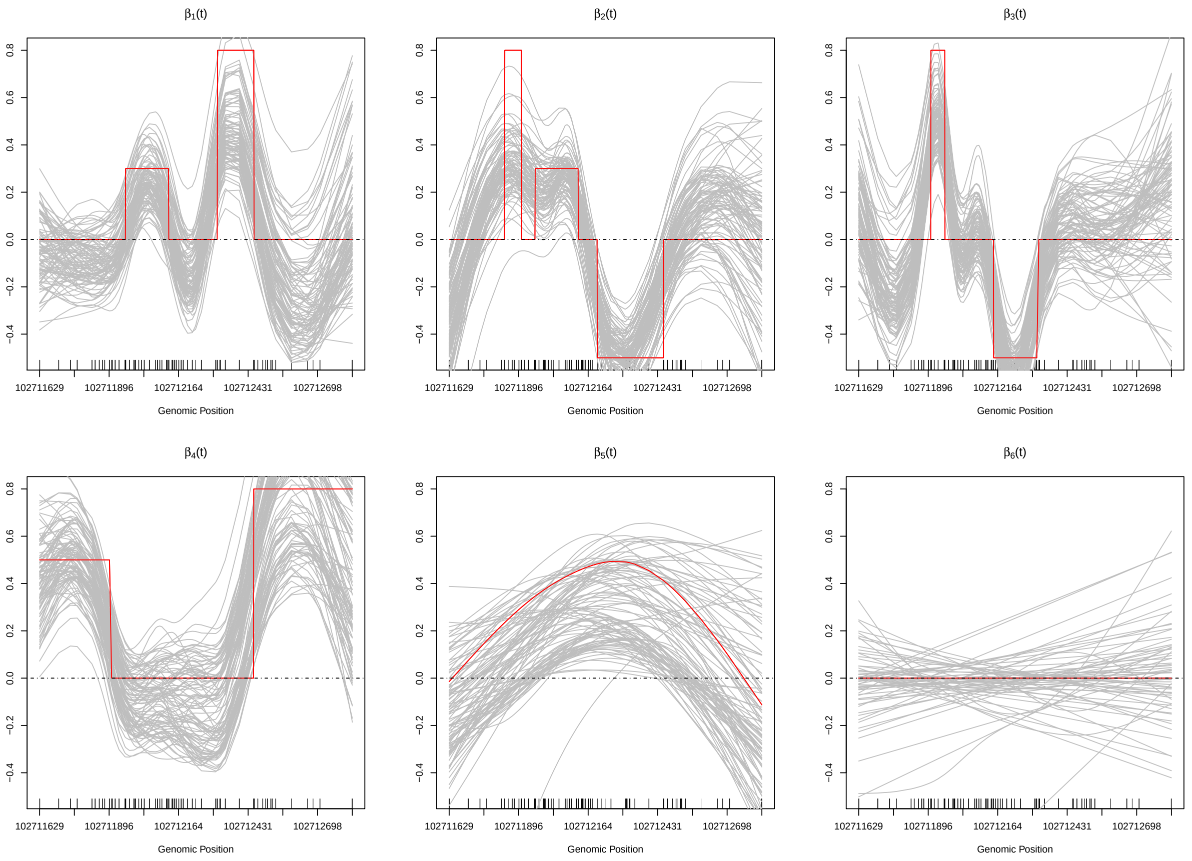

Figure S9. **GAM** estimates of the first 6 varying coefficients (gray) in Example 2 ($P = 100, \rho = 0$) over 100 simulation runs. The red curves are the truth.

Table S9. Average values of the CorRaw and CorTrans over 100 simulations for simulation **Examples 1 and 2**. Standard deviations are given in parentheses.

	CorRaw				CorTrans			
ρ	SSP	SSP0	gLASSO	GAM	SSP	SSP0	gLASSO	GAM
Example 1 (smooth, $P_{true} = 5, P = 100$)								
0	0.790(0.020)	0.788(0.020)	0.787(0.020)	0.552(0.111)	0.769(0.020)	0.766(0.020)	0.765(0.020)	0.542(0.109)
0.3	0.789(0.018)	0.787(0.018)	0.786(0.018)	0.528(0.145)	0.773(0.019)	0.771(0.019)	0.770(0.019)	0.526(0.144)
0.7	0.789(0.017)	0.788(0.017)	0.787(0.017)	0.525(0.147)	0.780(0.017)	0.778(0.018)	0.778(0.017)	0.533(0.144)
Example 1 (smooth, $P_{true} = 5, P = 1000$)								
0	0.788(0.019)	0.785(0.020)	0.783(0.020)	NA†	0.766(0.019)	0.763(0.020)	0.761(0.021)	NA
0.3	0.789(0.015)	0.787(0.016)	0.786(0.015)	NA	0.773(0.016)	0.770(0.016)	0.769(0.016)	NA
Example 2 (nonsmooth, $P_{true} = 5, P = 100$)								
0	0.655(0.019)	0.655(0.019)	0.658(0.019)	0.467(0.082)	0.658(0.023)	0.658(0.023)	0.661(0.021)	0.479(0.080)
0.3	0.669(0.019)	0.668(0.019)	0.670(0.020)	0.418(0.105)	0.669(0.022)	0.669(0.022)	0.670(0.023)	0.428(0.106)
Example 2 (nonsmooth, $P_{true} = 5, P = 1000$)								
0	0.645(0.024)	0.645(0.024)	0.648(0.025)	NA	0.649(0.026)	0.648(0.026)	0.651(0.027)	NA

	RMSE			
ρ	SSP	SSP0	gLASSO	GAM
Example 1 (smooth, $P_{true} = 5, P = 100$)				
0	0.412(0.007)	0.414(0.009)	0.415(0.009)	0.582(0.582)
0.3	0.411(0.007)	0.414(0.008)	0.415(0.008)	0.581(0.581)
0.7	0.409(0.007)	0.412(0.008)	0.413(0.008)	0.575(0.575)
Example 1 (smooth, $P_{true} = 5, P = 1000$)				
0	0.417(0.009)	0.420(0.011)	0.421(0.011)	NA
0.3	0.416(0.010)	0.418(0.011)	0.419(0.011)	NA
Example 2 (nonsmooth, $P_{true} = 5, P = 100$)				
0	0.425(0.010)	0.425(0.010)	0.424(0.010)	0.515(0.515)
0.3	0.424(0.011)	0.425(0.011)	0.423(0.010)	0.545(0.545)
Example 2 (nonsmooth, $P_{true} = 5, P = 1000$)				
0	0.430(0.013)	0.431(0.013)	0.430(0.013)	NA

† GAM involves no sparsity regularizations and cannot estimate a model with 1000 smooth components for $N = 50$.

Table S10. Average values of the deviance errors, RMSE CorRaw and CorTrans over 100 simulations using the **adaptive** SSP, SSP0 and gLASSO. Standard deviations are given in parentheses.

Example 1 (smooth, $P_{true} = 5, P = 100, \rho = 0$)								
	Deviance				RMSE			
ρ	^{a}SSP	^{a}SSP0	^{a}SSP-fixα	^{a}gLASSO	^{a}SSP	^{a}SSP0	^{a}SSP-fixα	^{a}gLASSO
0	0.016(0.007)	0.020(0.008)	0.016(0.008)	0.023(0.010)	0.408(0.006)	0.409(0.007)	0.408(0.408)	0.410(0.007)
	CorRaw				CorTrans			
	^{a}SSP	^{a}SSP0	^{a}SSP-fixα	^{a}gLASSO	^{a}SSP	^{a}SSP0	^{a}SSP-fixα	^{a}gLASSO
0	0.791(0.019)	0.790(0.019)	0.791(0.019)	0.790(0.019)	0.771(0.020)	0.770(0.020)	0.771(0.020)	0.769(0.020)

Table S11. Average values of the deviance errors, RMSE CorRaw and CorTrans over 100 simulations using the **the 1 SE rule** for SSP, SSP0 and gLASSO. Standard deviations are given in parentheses.

Example 1 (smooth, $P_{true} = 5, P = 100, \rho = 0$)						
	Deviance			RMSE		
ρ	SSP^{1SE}	SSP0^{1SE}	gLASSO^{1SE}	SSP^{1SE}	SSP0^{1SE}	gLASSO^{1SE}
0	0.060(0.028)	0.070(0.033)	0.070(0.032)	0.420(0.011)	0.422(0.012)	0.422(0.012)
	CorRaw			CorTrans		
ρ	SSP^{1SE}	SSP0^{1SE}	gLASSO^{1SE}	SSP^{1SE}	SSP0^{1SE}	gLASSO^{1SE}
0	0.785(0.020)	0.783(0.021)	0.783(0.021)	0.762(0.021)	0.761(0.021)	0.760(0.021)

Table S12. Average values of the number of TP and FP for simulation examples 1 and 2, using the **adaptive** SSP, SSP0, and gLASSO. Standard deviations are given in parentheses.

Example 1 (smooth, $P_{true} = 5, P = 100, \rho = 0$)								
	TP				FP			
ρ	^{a}SSP	^{a}SSP0	^{a}SSP-fixα	^{a}gLASSO	^{a}SSP	^{a}SSP0	^{a}SSP-fixα	^{a}gLASSO
0	4.99(0.10)	4.98(0.14)	4.99(0.10)	4.95(0.22)	16.18(6.53)	15.56(5.38)	16.38(6.72)	15.97(4.79)

Table S13. Average values of the number of TP and FP for simulation examples 1 and 2, using the **1 SE rule** for SSP, SSP0, and gLASSO. Standard deviations are given in parentheses.

Example 1 (smooth, $P_{true} = 5, P = 100, \rho = 0$)						
	TP			FP		
ρ	SSP^{1SE}	SSP0^{1SE}	gLASSO^{1SE}	SSP^{1SE}	SSP0^{1SE}	gLASSO^{1SE}
0	4.58(0.61)	4.59(0.62)	4.68(0.55)	1.77(1.98)	3.10(3.29)	2.93(3.26)

Table S14. Integrated Squared Bias (IBIAS^2), Integrated Variance (IVAR) and Integrated Mean Square Error (IMSE) of the first 10 varying coefficients of **Example 2 (non smooth)**, using SSP, SSP0, group LASSO and GAM.

		IBIAS^2				IVAR				IMSE			
ρ		SSP	SSP0	gLASSO	GAM	SSP	SSP0	gLASSO	GAM	SSP	SSP0	gLASSO	GAM
Example 2 (nonsmooth, $P = 100$)													
	$\beta_1(t)$	3.641	3.698	3.353	4.095	0.235	0.234	0.208	1.839	3.876	3.931	3.562	5.934
	$\beta_2(t)$	4.874	4.900	4.956	4.949	0.235	0.242	0.264	1.553	5.109	5.142	5.220	6.501
	$\beta_3(t)$	4.460	4.248	4.030	3.794	0.315	0.301	0.321	1.741	4.775	4.548	4.352	5.535
	$\beta_4(t)$	3.089	3.463	2.969	2.734	0.352	0.382	0.302	3.495	3.441	3.845	3.271	6.230
0	$\beta_5(t)$	0.385	0.438	0.534	3.977	0.296	0.316	0.294	3.626	0.682	0.754	0.828	7.603
	$\beta_6(t)$	1.7e-04	1.4e-04	6.3e-05	3.8e-02	1.4e-02	1.4e-02	9.6e-03	1.641	1.4e-02	1.4e-02	9.7e-03	1.679
	$\beta_7(t)$	7.2e-05	1.2e-04	1.4e-04	3.7e-02	8.6e-03	8.9e-03	8.4e-03	0.903	8.6e-03	9.1e-03	8.5e-03	0.940
	$\beta_8(t)$	1.6e-04	2.3e-04	2.1e-04	1.5e-02	1.3e-02	1.4e-02	1.1e-02	1.659	1.3e-02	1.4e-02	1.1e-02	1.674
	$\beta_9(t)$	1.1e-04	1.2e-04	1.3e-04	4.4e-02	9.2e-03	1.0e-02	5.9e-03	0.902	9.3e-03	1.0e-02	6.1e-03	0.946
	$\beta_{10}(t)$	4.6e-05	6.9e-05	4.7e-05	1.6e-03	1.1e-02	1.1e-02	5.9e-03	1.214	1.1e-02	1.1e-02	6.0e-03	1.215
	$\sum_1^{100}$	16.460	16.757	15.851	21.327	2.470	2.525	2.104	119.279	18.930	19.282	17.955	140.606
	$\beta_1(t)$	3.905	3.964	3.481	3.519	0.281	0.285	0.290	3.552	4.186	4.249	3.771	7.072
	$\beta_2(t)$	5.033	5.081	5.141	4.807	0.327	0.329	0.357	2.140	5.359	5.410	5.498	6.947
	$\beta_3(t)$	4.400	4.226	3.899	3.772	0.328	0.313	0.298	2.112	4.728	4.540	4.197	5.883
	$\beta_4(t)$	3.594	4.033	3.080	3.168	0.558	0.599	0.418	4.366	4.152	4.632	3.498	7.534
0.3	$\beta_5(t)$	0.415	0.471	0.638	3.394	0.328	0.354	0.386	4.819	0.743	0.825	1.025	8.212
	$\beta_6(t)$	6.0e-04	6.1e-04	5.5e-04	5.3e-03	9.9e-03	9.5e-03	8.0e-03	1.799	1.0e-02	1.0e-02	8.6e-03	1.804
	$\beta_7(t)$	4.1e-04	4.2e-04	4.6e-04	4.4e-03	1.3e-02	1.2e-02	1.2e-02	1.700	1.3e-02	1.3e-02	1.2e-02	1.705
	$\beta_8(t)$	5.1e-04	4.1e-04	3.3e-04	5.0e-03	1.2e-02	1.1e-02	8.4e-03	1.338	1.3e-02	1.2e-02	8.7e-03	1.343
	$\beta_9(t)$	3.5e-04	3.9e-04	4.4e-04	6.1e-02	1.2e-02	1.3e-02	9.9e-03	1.513	1.2e-02	1.3e-02	1.0e-02	1.573
	$\beta_{10}(t)$	1.2e-03	1.2e-03	9.6e-04	1.5e-02	1.4e-02	1.4e-02	1.3e-02	1.797	1.5e-02	1.5e-02	1.4e-02	1.812
	$\sum_1^{100}$	17.368	17.797	16.255	20.825	2.940	2.974	2.522	175.293	20.308	20.770	18.777	196.118
nonsmooth, $P = 1000$													
	$\beta_1(t)$	4.283	4.358	4.001	NA	0.308	0.306	0.310	NA	4.591	4.664	4.311	NA
	$\beta_2(t)$	5.448	5.476	5.601	NA	0.357	0.366	0.392	NA	5.805	5.842	5.993	NA
	$\beta_3(t)$	4.975	4.752	4.464	NA	0.319	0.313	0.325	NA	5.293	5.066	4.789	NA
	$\beta_4(t)$	4.653	5.201	4.098	NA	0.585	0.603	0.539	NA	5.238	5.804	4.637	NA
0	$\beta_5(t)$	1.035	1.112	1.250	NA	0.421	0.437	0.470	NA	1.456	1.550	1.720	NA
	$\beta_6(t)$	6.5e-06	6.1e-06	5.2e-06	NA	9.5e-04	8.1e-04	7.8e-04	NA	9.5e-04	8.1e-04	7.8e-04	NA
	$\beta_7(t)$	7.4e-06	5.5e-06	5.7e-06	NA	1.5e-03	1.7e-03	1.1e-03	NA	1.6e-03	1.7e-03	1.1e-03	NA
	$\beta_8(t)$	5.5e-06	3.3e-06	1.4e-06	NA	6.8e-04	7.2e-04	2.4e-04	NA	6.8e-04	7.2e-04	2.4e-04	NA
	$\beta_9(t)$	1.1e-05	1.0e-05	1.4e-05	NA	1.4e-03	1.3e-03	1.2e-03	NA	1.4e-03	1.3e-03	1.2e-03	NA
	$\beta_{10}(t)$	1.7e-05	1.7e-05	8.5e-06	NA	1.2e-03	1.4e-03	1.4e-03	NA	1.2e-03	1.4e-03	1.4e-03	NA
	$\sum_1^{1000}$	20.404	20.909	19.423	NA	2.910	2.940	2.784	NA	23.314	23.850	22.206	NA

Table S15. Integrated Squared Bias ($IBIAS^2$), Integrated Variance (IVAR) and Integrated Mean Square Error (IMSE) of the first 10 varying coefficients of **Example 1** ($P = 1000$), using SSP, SSP0, and group LASSO.

		$IBIAS^2$			IVAR			IMSE		
ρ		SSP	SSP0	gLASSO	SSP	SSP0	gLASSO	SSP	SSP0	gLASSO
	$\beta_1(t)$	0.947	1.362	1.560	0.409	0.539	0.641	1.356	1.901	2.201
	$\beta_2(t)$	1.356	1.960	1.845	0.284	0.292	0.310	1.641	2.252	2.156
	$\beta_3(t)$	1.345	2.013	2.113	0.586	0.745	0.802	1.931	2.757	2.915
	$\beta_4(t)$	0.966	1.241	1.365	0.134	0.116	0.104	1.100	1.357	1.469
	$\beta_5(t)$	1.898	2.233	2.261	0.343	0.407	0.401	2.241	2.640	2.662
0	$\beta_6(t)$	8.7e-07	3.8e-06	2.6e-06	2.4e-04	5.1e-04	4.7e-04	2.4e-04	5.1e-04	4.8e-04
	$\beta_7(t)$	2.1e-06	1.4e-05	1.4e-05	9.7e-04	2.0e-03	1.4e-03	9.7e-04	2.0e-03	1.5e-03
	$\beta_8(t)$	5.3e-06	1.1e-05	1.1e-05	8.8e-04	1.0e-03	1.5e-03	8.9e-04	1.0e-03	1.5e-03
	$\beta_9(t)$	8.1e-06	7.4e-06	2.1e-05	6.6e-04	4.7e-04	7.0e-04	6.7e-04	4.8e-04	7.2e-04
	$\beta_{10}(t)$	9.6e-06	8.8e-06	5.7e-06	1.2e-03	7.1e-04	4.9e-04	1.3e-03	7.2e-04	4.9e-04
	$\sum_1^{1000}$	6.523	8.819	9.156	2.623	3.070	3.197	9.146	11.889	12.353
	$\beta_1(t)$	0.636	0.908	1.165	0.487	0.623	0.733	1.123	1.531	1.899
	$\beta_2(t)$	1.461	2.014	1.908	0.332	0.351	0.357	1.793	2.365	2.265
	$\beta_3(t)$	1.070	1.590	1.641	0.354	0.479	0.530	1.424	2.070	2.172
	$\beta_4(t)$	0.893	1.093	1.141	0.149	0.157	0.149	1.042	1.249	1.290
	$\beta_5(t)$	1.291	1.368	1.356	0.487	0.554	0.537	1.778	1.922	1.893
0.3	$\beta_6(t)$	2.5e-04	3.7e-04	3.9e-04	2.8e-03	4.2e-03	4.7e-03	3.1e-03	4.5e-03	5.1e-03
	$\beta_7(t)$	3.4e-04	3.4e-04	2.1e-04	3.8e-03	4.5e-03	4.2e-03	4.1e-03	4.9e-03	4.4e-03
	$\beta_8(t)$	2.5e-04	4.2e-04	5.3e-04	7.6e-03	1.0e-02	1.4e-02	7.8e-03	1.1e-02	1.4e-02
	$\beta_9(t)$	2.0e-04	2.6e-04	2.8e-04	3.6e-03	4.8e-03	4.6e-03	3.8e-03	5.1e-03	4.9e-03
	$\beta_{10}(t)$	5.5e-04	7.5e-04	9.2e-04	6.3e-03	8.3e-03	9.6e-03	6.8e-03	9.1e-03	1.0e-02
	$\sum_1^{1000}$	5.360	6.984	7.224	2.493	2.950	3.100	7.853	9.934	10.324

Table S16. Average values of the deviance errors, RMSE, CorRaw and CorTrans over 100 simulations for simulation **Examples 3 and 4**. Standard deviations are given in parentheses.

		Deviance				RMSE			
P	P_{true}	SSP	SSP0	gLASSO	GAM	SSP	SSP0	gLASSO	GAM
50	5	0.049(0.021)	0.064(0.025)	0.063(0.023)	0.634(0.405)	0.405(0.009)	0.408(0.010)	0.409(0.010)	0.482(0.046)
	10	0.148(0.047)	0.169(0.047)	0.162(0.041)	0.690(0.345)	0.429(0.013)	0.433(0.014)	0.431(0.013)	0.495(0.038)
100	5	0.071(0.032)	0.080(0.030)	0.077(0.023)	0.593(0.243)	0.412(0.010)	0.414(0.010)	0.414(0.010)	0.483(0.030)
	10	0.190(0.069)	0.204(0.068)	0.194(0.050)	0.915(0.671)	0.438(0.016)	0.440(0.016)	0.439(0.014)	0.520(0.063)
150	5	0.072(0.030)	0.084(0.032)	0.083(0.028)	0.596(0.346)	0.413(0.011)	0.415(0.011)	0.414(0.010)	0.483(0.038)
	10	0.192(0.068)	0.202(0.063)	0.192(0.047)	0.918(0.401)	0.439(0.018)	0.441(0.018)	0.439(0.016)	0.525(0.046)
200	5	0.087(0.040)	0.098(0.042)	0.092(0.035)	0.598(0.276)	0.415(0.012)	0.417(0.013)	0.416(0.011)	0.484(0.034)
	10	0.236(0.104)	0.242(0.092)	0.227(0.070)	0.804(0.416)	0.444(0.021)	0.445(0.020)	0.443(0.018)	0.511(0.046)
1000	5	0.108(0.038)	0.115(0.036)	0.113(0.032)	NA	0.415(0.010)	0.417(0.009)	0.417(0.009)	NA
	10	0.272(0.076)	0.272(0.073)	0.274(0.073)	NA	0.447(0.018)	0.447(0.017)	0.447(0.017)	NA

		CorRaw				CorTrans			
P	P_{true}	SSP	SSP0	gLASSO	GAM	SSP	SSP0	gLASSO	GAM
50	5	0.757(0.023)	0.753(0.023)	0.753(0.022)	0.634(0.079)	0.729(0.022)	0.725(0.022)	0.725(0.021)	0.608(0.079)
	10	0.710(0.039)	0.703(0.039)	0.703(0.039)	0.569(0.087)	0.677(0.038)	0.669(0.038)	0.670(0.038)	0.542(0.084)
100	5	0.749(0.026)	0.747(0.026)	0.748(0.025)	0.616(0.067)	0.720(0.026)	0.717(0.025)	0.718(0.025)	0.588(0.065)
	10	0.700(0.035)	0.695(0.036)	0.696(0.034)	0.521(0.136)	0.665(0.035)	0.659(0.035)	0.661(0.033)	0.493(0.131)
150	5	0.751(0.024)	0.749(0.023)	0.748(0.023)	0.633(0.051)	0.723(0.023)	0.720(0.022)	0.720(0.022)	0.606(0.050)
	10	0.702(0.036)	0.699(0.035)	0.698(0.036)	0.513(0.113)	0.668(0.034)	0.664(0.033)	0.663(0.034)	0.487(0.108)
200	5	0.751(0.025)	0.749(0.025)	0.749(0.025)	0.630(0.065)	0.722(0.025)	0.719(0.024)	0.719(0.024)	0.601(0.063)
	10	0.695(0.048)	0.692(0.048)	0.692(0.047)	0.533(0.102)	0.660(0.047)	0.657(0.046)	0.656(0.046)	0.505(0.098)
1000	5	0.747(0.027)	0.745(0.027)	0.745(0.027)	NA	0.717(0.026)	0.716(0.026)	0.716(0.026)	NA
	10	0.683(0.047)	0.682(0.046)	0.679(0.045)	NA	0.647(0.045)	0.646(0.044)	0.643(0.043)	NA

D Additional data application methods and results

Data description In this study, participants were sampled from the CARTaGENE cohort, a population-based cohort of 43,000 subjects aged between 40 and 69 years, from Quebec, Canada. Firstly, the serum ACPA levels were measured for 3600 randomly-sampled individuals from the CARTaGENE cohort (`https://www.cartagene.qc.ca/`), based upon which individuals were classified as either ACPA positive or ACPA negative. Then, the whole blood samples of the ACPA positive individuals, and a selected subset of age-sex-and-smoking-status-matched ACPA negative individuals were sent for Targeted Custom Capture Bisulfite Sequencing. Specifically, the sequencing used blood cell-specific immune panels that cover the majority of human gene promoters, active regulatory regions observed in blood, blood-cell-lineage-specific enhancer regions and CpGs from Illumina Human Methylation 450 Bead Chips. Cell type proportions in the blood samples were also measured at the time of the sampling. We excluded the samples who reported a diagnosis of RA before the CARTaGENE study started and samples with missing information on cell type proportions. In our final analysis, there are 45 ACPA-positive and 53 ACPA-negative subjects.

We focused on autosomal analysis only. To better translate the methylation information in the sequence of the genome into biologically relevant knowledge, we defined the gene-specific methylation regions as the first exon and 2000 base pairs upstream of each protein-coding gene. For simplicity, we focused on regions with at least 20 CpG sites, and our final analysis includes 12,283 methylation regions, covering around 1.4 million CpG sites. For details on these regions, refer to Figure S10 for the distribution of the number of CpG sites, and the number of SNPs tested for each region.

SNP calling and imputing: Genotyping data was fetched from CARTaGENE, the details of pipeline used for the quality control of CARTaGENEs genotyping data can be found on their website. Because the data was generated through 5 genotyping arrays: Axiom, Omni 2.5M, GSA760, GSA4224, GSA5300, the genotyping data on the same samples were then imputed using the Sanger imputation service, the guidelines listed on the website were followed.

Imputed SNPs with INFO score greater than 0.4 were considered as acceptable well-imputed variants. Taking the sample size into consideration, we retained only SNPs with minor allele frequency (MAF) greater than 0.05 for subsequent analyses. Multiallelic sites and multiple variants sharing the same basepair coordinate or allele codes were excluded. We end up with around 4.4 million SNPs.

Stage I: Regional mQTL Mapping

Smoothness parameter selection vs. CpG density in mQTL analysis. Fig. S20 shows the distribution of the number of CpG sites per region versus the best-selected smoothness control parameter (α) across all 12,283 methylation regions and the 1,014 regions with detected mQTLs In the full set of 12,283 regions, the selected smoothness control parameters (α) spanned the entire range, with 47.7% of regions selecting $\alpha = 0$ and

7.8% selecting $\alpha = 0.999$ as the best value under cross-validation. While $\alpha = 0$ was most frequently selected, especially among bigger regions with more CpGs, the distribution of α in smaller regions was more dispersed, with both low and high values observed.

Among the 1,014 regions with detected mQTLs, the α distribution shifted substantially: only 17.8% selected $\alpha = 0$, while the majority selected mid-to-high values (33.7% at 0.8 and 28.2% at 0.999). These findings suggest that smoother fits are often preferred in regions with underlying geneticmethylation associations, possibly reflecting more structured or coordinated methylation changes. Biologically, this pattern may indicate that in functionally active regions, moderate-to-high smoothness helps capture localized regulatory variation, whereas regions without strong signals exhibit more heterogeneous smoothness behavior.

Comparison with marginal mQTL results

Supplementary FigureS21 shows the estimated coefficients from sparseSOMNiBUS alongside univariate p-values obtained using the *snpMatrix* package, with SNP-CpG pairs sorted by decreasing marginal significance. This comparison was performed without LD pruning, allowing more overlap between SNP-CpG pairs analyzed by both method. Due to a 12-hour wall time limit, this figure includes only regions for which sparseSOMNiBUS produced results within the allowed runtime.

Most strong marginal associations correspond to large penalized estimates, indicating consistency between the two approaches (evident in the left portion of the plot with tall blue bars aligned with low p-values). However, because sparseSOMNiBUS fits a joint model across all candidate SNPs, it can assign large coefficients to SNPs with moderate univariate signals if they provide complementary explanatory power—these appear as isolated spikes on the right side of the plot. Conversely, some marginally significant SNPCpG pairs receive attenuated coefficients after accounting for other predictors in the model, as indicated by short bars near the left tail. This contrast highlights the methods ability to recover key signals while refining variable selection based on joint effects.

Functional enrichment analysis for mQTL SNPs

To assess the functional relevance of identified mQTLs, we performed enrichment analyses using gene annotation, CpG region types, and enhancer elements. The analysis included 23,022 mQTL SNPs and 366,010 background SNPs after LD pruning ($r^2 < 0.2$). We quantified SNP overlap with genomic annotations and compared the enrichment frequency to that of the LD-pruned background set. Gene context annotations (e.g., promoters, exons, UTRs, introns) and CpG region types (islands, shores, shelves, inter-CGI) were obtained using the *annotatr* package. Enhancer regions were defined using ChromHMM 15-state mnemonics from the Roadmap Epigenomics Project for primary whole blood (E062), restricted to the Enhancers and Genic enhancers states. For each category, enrichment was evaluated using Fishers exact test, yielding odds ratios and p-values.

The enrichment analysis revealed distinct patterns of mQTL localization across genomic annotations. Within gene contexts (Figure S22(A)), mQTL SNPs were most strongly enriched in 15kb upstream regions (odds ratio ≈ 1.3; $-\log_{10}(p) > 20$), indicating a preferential localization near gene promoters. Moderate enrichment was also observed in 5UTRs, exons, and first exons. In contrast, intronic and intergenic regions were gen-

erally under-represented. For CpG features (Figure S22 (B)), mQTL SNPs were strongly over-represented in CpG shores and shelves, and moderately enriched in CpG islands. Conversely, they were significantly under-represented in inter-CGI regions. Finally, enhancer annotations (Figure S22 (C)) showed modest enrichment for distal enhancers (OR = 1.14, $p = 0.01$), although this finding is nominal and may not withstand multiple testing correction. Genic enhancers exhibited slightly higher odds ratios but did not reach statistical significance at the $\alpha = 0.05$ threshold. Together, these results support the functional localization of mQTLs to regulatory hotspots, particularly 15kb upstream regions, CpG shores, and enhancer elements in whole blood.

Stage II: Association Analysis Adjusting for mQTLs

In Stage II, we tested the association between regional DNA methylation and ACPA status (45 positive, 53 negative) using a quasi-binomial varying coefficient mixed-effects model (Zhao et al., 2024). This model accounts for both overdispersion and within-subject correlation across CpG sites, and provides region-level inference.

The association analyses adjusted for age, sex, smoking status, blood cell-type proportions, and the top 3 genotype principal components (PCs). These PCs were derived from genome-wide SNP data (4.4 million variants) without LD pruning, to capture global population structure (see Figure S23). In addtion, each model included the mQTL identified in Stage I as covariates to account for local genetic influences.

Specifically, we modeled the methylation outcome as follows:

$$
\begin{aligned}
\log \frac{\pi_{ij}}{1-\pi_{ij}} &= \beta_0(t_{ij}) + \sum_{p=1}^{P} \beta_p(t_{ij}) Z_{ip} + u_i, \\
u_i &\overset{iid}{\sim} N(0, \sigma_0^2) \\
\mathbb{V}\mathrm{ar}(Y_{ij} \mid u_i) &= \phi X_{ij} \pi_{ij} (1 - \pi_{ij})
\end{aligned}
$$

where $\pi_{ij} = \mathbb{E}\left(Y_{ij} \mid u_i\right)/X_{ij}$ is the individual's methylation proportion at site j for subject i, and $\beta_0(t_{ij})$ and $\{\beta_p(t_{ij})\}_{p=1}^{P}$ are functional parameters for the intercept and effects of covariates, including ACPA status, mQTL genotypes, and other adjusting variables. The subject-spcific random effect u_i accounts for within-subject correlation. ϕ is the multiplicative dispersion parameter.

Figure S24 contrasts the region-level ACPA association results with and without mQTL adjustment. Adjustment revealed stronger association for genes such as *SDHAP3*, *MESTIT1*, and *GNAS-AS1*, suggesting that genetic confounding had previously masked ACPA-related methylation changes. In contrast, signals in regions such as *SMIM24* and *RABEP1* weakened after adjustment, suggesting those effects were largely genetically driven.

These findings underscore the value of sparse mQTL mapping in refining epigenetic association analyses by disentangling true phenotype-related methylation patterns from genotype-driven variation.

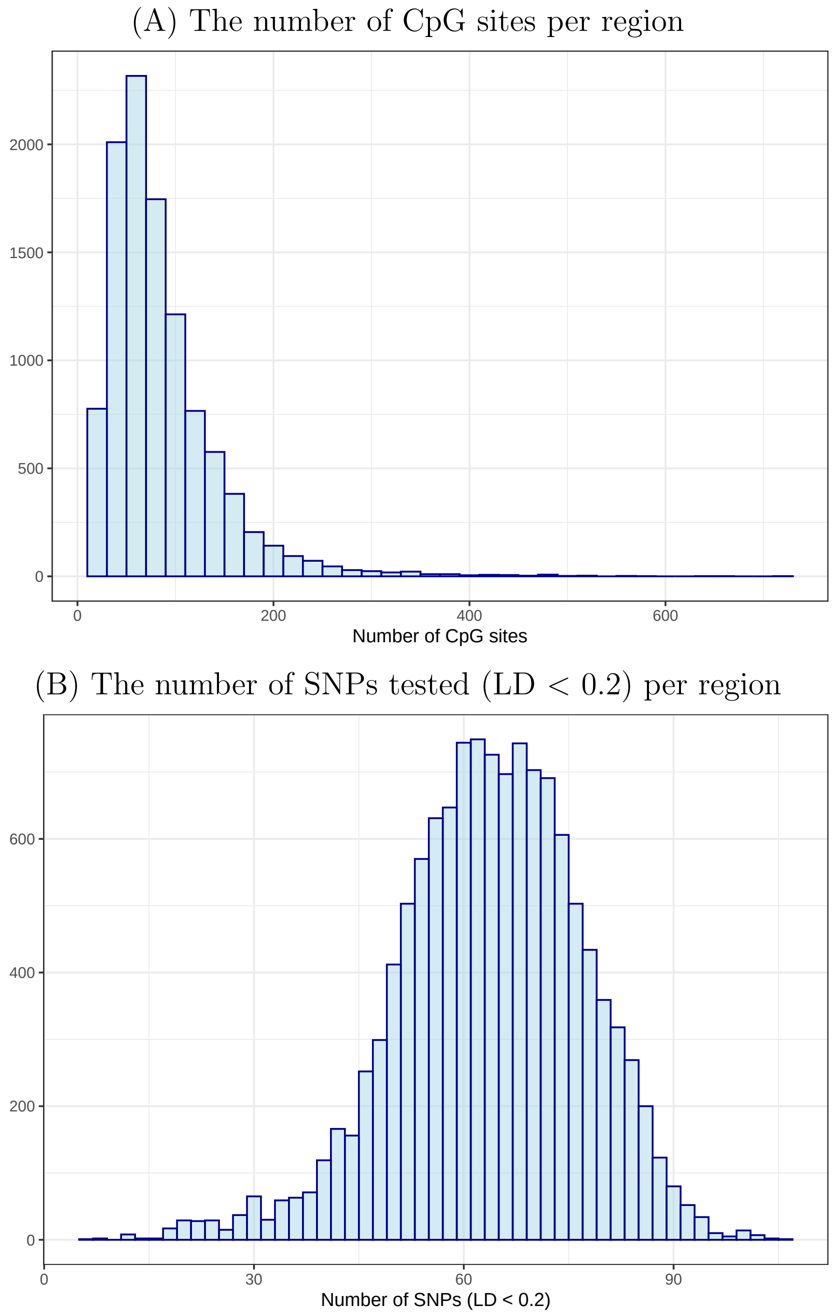

Figure S10. Distribution of region sizes in the mQTL analysis for the 12283 methylation regions analyzed in the data application.. (A) Number of CpG sites per methylation region. Regions were defined as the first exon plus 2,000bp upstream of each protein-coding gene and filtered to retain those with ≥ 20 CpGs. The distribution has a mean of 90.0 CpGs per region, with a 1st quartile of 49, median of 74, and 3rd quartile of 112. (B) Number of SNPs tested per region after LD pruning at threshold 0.2. SNPs were selected within $\pm$2.5Mb of each region and pruned using a sliding window to remove SNPs with pairwise correlation $>$0.2. The number of SNPs per region ranged from 6 to 106, with a mean of 64.3, 1st quartile of 56, median of 65, and 3rd quartile of 73.

References

Wood, S. N. (2017). *Generalized additive models: an introduction with R.* CRC press.

Zhao, K., Oualkacha, K., Zeng, Y., Shen, C., Klein, K., Lakhal-Chaieb, L., Labbe, A., Pastinen, T., Hudson, M., Colmegna, I., et al. (2024). Addressing dispersion in mis-measured multivariate binomial outcomes: A novel statistical approach for detecting differentially methylated regions in bisulfite sequencing data. *Statistics in medicine*, 43(20):3899–3920.

Table S17. Integrated Squared Bias ($IBIAS^2$), Integrated Variance (IVAR) and Integrated Mean Square Error (IMSE) of the first 5 varying coefficients of **Examples 3 and 4** ($N = 20, P = 50, 100$), using SSP, SSP0, group LASSO and GAM.

		$IBIAS^2$				IVAR				IMSE			
P	P_{true}	SSP	SSP0	gLASSO	GAM	SSP	SSP0	gLASSO	GAM	SSP	SSP0	gLASSO	GAM
50	5	0.568	0.870	1.090	2.426	3.0e-01	3.9e-01	4.2e-01	1.6e+00	0.868	1.256	1.512	4.048
50	5	1.480	2.165	2.083	1.359	5.8e-01	5.8e-01	5.9e-01	3.6e+00	2.057	2.744	2.675	4.977
50	5	0.584	0.946	1.091	1.709	4.2e-01	5.1e-01	5.4e-01	2.8e+00	1.005	1.451	1.627	4.477
50	5	0.921	1.169	1.290	0.308	2.9e-01	2.7e-01	2.3e-01	1.7e+00	1.211	1.441	1.523	2.017
50	5	0.864	1.152	1.241	0.160	2.9e-01	2.7e-01	2.3e-01	2.2e+00	1.156	1.419	1.470	2.387
50	10	0.829	1.058	1.174	1.662	6.7e-01	7.0e-01	7.0e-01	2.3e+00	1.499	1.760	1.872	3.986
50	10	1.530	2.066	2.075	2.016	7.5e-01	7.6e-01	7.0e-01	3.1e+00	2.283	2.828	2.775	5.083
50	10	0.974	1.212	1.317	1.216	7.4e-01	8.2e-01	8.2e-01	2.4e+00	1.712	2.028	2.138	3.650
50	10	1.047	1.191	1.301	0.319	3.6e-01	3.2e-01	2.7e-01	1.7e+00	1.403	1.514	1.568	2.033
50	10	0.974	1.172	1.316	0.206	4.4e-01	3.9e-01	3.4e-01	1.9e+00	1.417	1.565	1.654	2.071
100	5	1.200	1.402	1.554	3.159	4.4e-01	4.3e-01	4.3e-01	6.9e-01	1.644	1.832	1.984	3.848
100	5	1.563	2.280	2.189	3.557	5.6e-01	5.1e-01	5.2e-01	1.5e+00	2.121	2.790	2.706	5.025
100	5	1.045	1.392	1.500	3.480	5.1e-01	5.3e-01	5.5e-01	9.2e-01	1.551	1.920	2.049	4.403
100	5	1.129	1.412	1.513	0.281	2.0e-01	1.6e-01	1.5e-01	1.5e+00	1.330	1.567	1.665	1.815
100	5	1.289	1.515	1.603	0.474	1.7e-01	1.5e-01	1.2e-01	8.9e-01	1.464	1.661	1.719	1.361
100	10	1.878	2.048	2.203	2.964	4.7e-01	4.5e-01	4.3e-01	9.2e-01	2.349	2.493	2.631	3.880
100	10	1.460	1.955	1.998	3.284	7.6e-01	7.4e-01	6.8e-01	1.5e+00	2.215	2.697	2.677	4.798
100	10	1.656	1.904	2.071	2.517	6.2e-01	5.9e-01	5.7e-01	2.2e+00	2.272	2.499	2.640	4.766
100	10	1.329	1.519	1.621	0.647	2.5e-01	2.0e-01	1.8e-01	1.5e+00	1.580	1.715	1.801	2.117
100	10	1.391	1.542	1.634	0.461	2.3e-01	1.9e-01	1.5e-01	1.3e+00	1.619	1.734	1.788	1.728

Table S18. Integrated Squared Bias (IBIAS^2), Integrated Variance (IVAR) and Integrated Mean Square Error (IMSE) of the first 5 varying coefficients of **Examples 3 and 4 (**$N = 20, P = 150, 200, 1000$**)**, using SSP, SSP0, group LASSO and GAM.

		IBIAS^2				IVAR				IMSE			
P	P_{true}	SSP	SSP0	gLASSO	GAM	SSP	SSP0	gLASSO	GAM	SSP	SSP0	gLASSO	GAM
150	5	1.326	1.638	1.849	3.720	3.6e-01	3.9e-01	3.9e-01	4.2e-01	1.684	2.027	2.238	4.144
150	5	1.818	2.566	2.501	3.287	4.3e-01	4.1e-01	4.2e-01	1.3e+00	2.249	2.981	2.922	4.610
150	5	1.314	1.678	1.817	3.623	5.6e-01	5.3e-01	5.4e-01	6.8e-01	1.879	2.212	2.352	4.299
150	5	1.464	1.647	1.726	0.614	1.5e-01	1.1e-01	8.7e-02	8.7e-01	1.611	1.760	1.813	1.483
150	5	1.266	1.531	1.585	0.460	2.0e-01	1.5e-01	1.3e-01	7.9e-01	1.467	1.678	1.717	1.249
150	10	2.311	2.473	2.531	3.117	3.8e-01	3.9e-01	4.1e-01	9.9e-01	2.695	2.863	2.940	4.106
150	10	1.853	2.460	2.514	3.619	6.1e-01	5.5e-01	5.0e-01	1.7e+00	2.467	3.013	3.016	5.317
150	10	1.887	2.068	2.164	3.644	6.2e-01	6.2e-01	6.1e-01	9.8e-01	2.507	2.691	2.772	4.628
150	10	1.434	1.572	1.677	0.879	2.3e-01	2.0e-01	1.6e-01	1.1e+00	1.668	1.775	1.832	2.007
150	10	1.445	1.616	1.703	0.764	1.8e-01	1.6e-01	1.4e-01	1.2e+00	1.626	1.774	1.847	1.945
200	5	1.552	1.757	1.917	4.151	5.6e-01	5.4e-01	5.0e-01	2.3e-01	2.110	2.292	2.417	4.381
200	5	1.670	2.438	2.290	4.455	4.3e-01	4.1e-01	4.3e-01	8.1e-01	2.102	2.851	2.718	5.265
200	5	1.629	2.030	2.115	4.433	4.1e-01	4.0e-01	4.1e-01	3.2e-01	2.035	2.426	2.525	4.751
200	5	1.494	1.665	1.735	0.957	1.6e-01	1.3e-01	1.2e-01	6.4e-01	1.657	1.793	1.851	1.601
200	5	1.536	1.727	1.751	0.705	9.5e-02	6.7e-02	6.8e-02	9.0e-01	1.631	1.793	1.820	1.604
200	10	2.147	2.276	2.390	3.570	5.8e-01	5.3e-01	4.9e-01	5.7e-01	2.726	2.803	2.876	4.137
200	10	2.153	2.664	2.685	4.331	6.2e-01	5.6e-01	5.4e-01	9.2e-01	2.777	3.229	3.221	5.248
200	10	2.832	3.002	3.092	4.917	4.4e-01	4.3e-01	4.2e-01	2.4e-02	3.275	3.433	3.511	4.941
200	10	1.648	1.738	1.799	1.139	2.2e-01	1.8e-01	1.5e-01	7.2e-01	1.868	1.921	1.948	1.864
200	10	1.613	1.721	1.798	1.064	1.3e-01	1.0e-01	8.6e-02	5.9e-01	1.745	1.824	1.884	1.659
1000	5	3.157	3.334	3.448	NA	2.3e-01	2.0e-01	1.7e-01	NA	3.385	3.532	3.618	NA
1000	5	2.676	3.262	3.189	NA	4.4e-01	4.0e-01	4.0e-01	NA	3.114	3.659	3.584	NA
1000	5	3.028	3.184	3.320	NA	3.7e-01	3.5e-01	3.1e-01	NA	3.403	3.530	3.627	NA
1000	5	1.862	1.953	1.997	NA	5.3e-02	4.2e-02	3.3e-02	NA	1.915	1.995	2.031	NA
1000	5	1.862	1.945	1.972	NA	6.2e-02	4.6e-02	4.0e-02	NA	1.925	1.992	2.012	NA
1000	10	3.936	3.961	3.986	NA	1.8e-01	1.6e-01	1.6e-01	NA	4.114	4.123	4.149	NA
1000	10	3.338	3.622	3.830	NA	4.9e-01	4.5e-01	4.1e-01	NA	3.825	4.069	4.236	NA
1000	10	3.642	3.750	3.885	NA	2.8e-01	2.5e-01	2.3e-01	NA	3.921	3.998	4.114	NA
1000	10	1.949	1.999	2.018	NA	3.5e-02	2.4e-02	2.3e-02	NA	1.984	2.023	2.041	NA
1000	10	1.934	1.955	1.976	NA	7.6e-02	7.9e-02	6.8e-02	NA	2.010	2.034	2.044	NA

Table S19. The **aggregated** Integrated Squared Bias (IBIAS^2), Integrated Variance (IVAR) and Integrated Mean Square Error (IMSE) across all the varying coefficents of **Examples 3 and 4** ($N = 20$), using SSP, SSP0, group LASSO and GAM.

		IBIAS^2				IVAR				IMSE			
P	P_{true}	SSP	SSP0	gLASSO	GAM	SSP	SSP0	gLASSO	GAM	SSP	SSP0	gLASSO	GAM
50	5	4.447	6.330	6.819	8.742	3.141	3.278	3.161	67.968	7.588	9.608	9.979	76.710
	10	12.162	14.919	15.893	13.916	8.636	8.631	7.924	80.401	20.798	23.550	23.817	94.317
100	5	6.256	8.031	8.385	13.132	3.274	3.124	3.041	56.516	9.530	11.155	11.426	69.648
	10	16.753	19.262	20.084	22.470	8.684	8.165	7.609	82.141	25.436	27.428	27.693	104.610
150	5	7.221	9.090	9.506	13.391	3.181	3.028	2.998	52.189	10.402	12.118	12.504	65.580
	10	19.276	21.556	22.272	27.436	8.313	7.857	7.274	76.277	27.589	29.413	29.547	103.712
200	5	7.909	9.644	9.833	16.301	3.009	2.835	2.690	54.774	10.918	12.479	12.523	71.075
	10	21.991	24.006	24.711	31.823	7.784	7.308	6.770	69.310	29.776	31.314	31.481	101.133
1000	5	12.617	13.707	13.954	NA	2.761	2.521	2.369	NA	15.379	16.228	16.323	NA
	10	30.592	31.621	32.434	NA	5.945	5.494	4.860	NA	36.537	37.116	37.295	NA

A. Mean methylation levels stratified by genotype at the top identified SNP for each gene-based methylation region. Individual samples are shown underneath using smaller points with slightly varied gradient colors.

ARRB2 (rs113739199) *MRI1* (rs1205169)

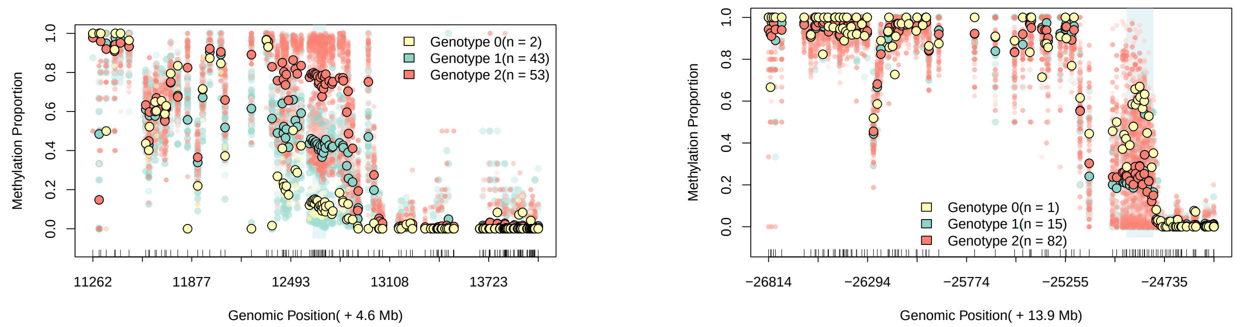

B. Estimated smooth effect of the top SNP across CpG positions, obtained from a joint model including all candidate SNPs in Panel C. Gene-based annotations are overlaid to provide genetic context.

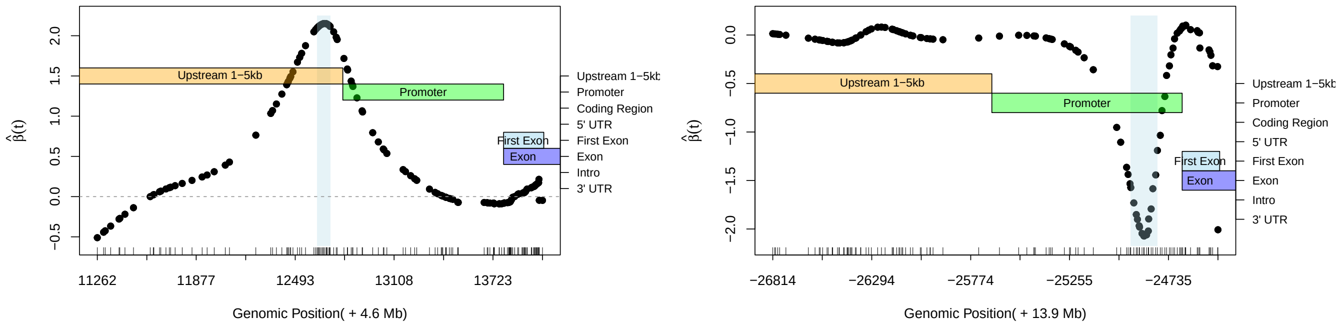

C. One-number summaries of SNP-region associations: peak effect estimates from individual SNP curves for all candidate SNPs within a 2.5Mb window. Each star represents a SNP positioned along the genomic axis; the methylation region is marked by a vertical blue dashed line.

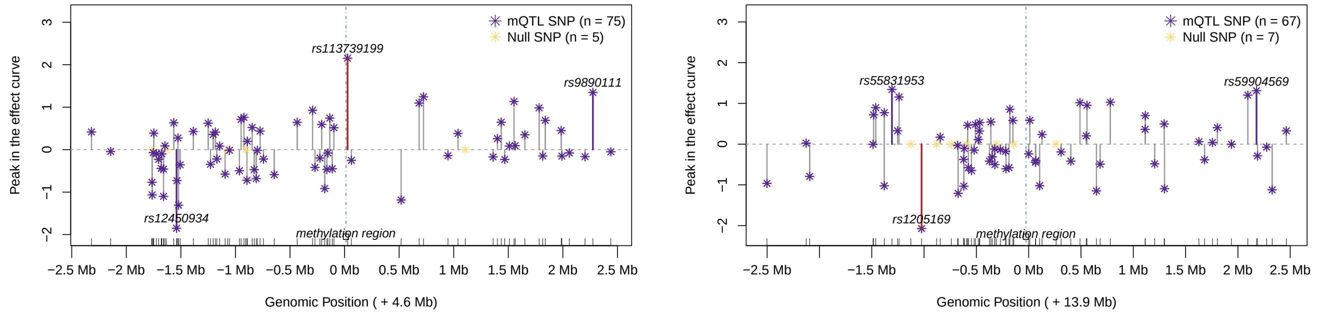

Figure S11. Regional mQTL patterns across two gene-based methylation regions. Shown are two representative regions—*ARRB2* and *MRI1*—ranked 5th and 6th, respectively, out of 12,569 gene-defined regions. The rows display (A) methylation proportions by genotype, (B) estimated smooth SNP effects, and (C) peak effect magnitudes across all candidate SNPs within a 2.5Mb window. The shaded region marks the top CpG site (defined as the site showing the peak signal with the top-associated SNP) and its surrounding context, defined as the 8 nearest upstream and 7 nearest downstream CpGs along the genomic axis. Together, these patterns illustrate the need to model both smooth and sparse genetic effects in regional mQTL analysis.

Table S20. Summary of results under different LD-pruning thresholds. For each gene, regional mQTL analyses were conducted using SNP sets pruned at LD thresholds of 0.2, 0.5, and 0.8. Due to computational constraints, this comparison was limited to a subset of representative genes. The corresponding computational times are presented in Table S21 and visualized in Figure S15.

	Gene-based Methylation Region					Top mQTL and Effect Summary								Overall Model Summary		
Rank	Gene	Chr	Region	Width (bp)	# of CpGs	LD cutoff	# of SNPs	Top SNP	SNP Pos	Subregion with $\lvert\beta(t)\rvert \geq \log(2)$	Peak CpG Pos	SNP-CpG Dis (bp)	Peak $\beta(t)$	# of mQTLs	% of mQTLs	α
2	RHOU	1	[-9521, -8890]	631	63	0.2	62	rs34530485	+1657936	[-9521, -9439]	-9521	-1667457	2.745	62	100	0
						0.5	505	rs113447861	+722923	[-9521, -9291]	-9458	-732381	-1.137	209	41.39	0
						0.8	1261	rs113447861	+722923	[-9506, -9235]	-9458	-732381	-1.003	109	8.64	0
						0.9	1777	rs4846856	+1655692	[-9521, -9439]	-9521	-1665213	0.982	101	5.68	0
3	MIR4520-1	17	[-415, +56]	471	24	0.2	79	rs138292190	+1790881	[-343, +56]	+56	-1790825	2.293	74	93.67	0.999
						0.5	643	rs9905053	+91371	[-343, +56]	+56	-91315	1.172	39	6.07	0.8
						0.8	1536	rs4796533	+2290	[-318, +56]	-120	-2410	-1.77	1	0.07	0.5
						0.9	2157	rs4796533	+2290	[-318, +56]	-120	-2410	-1.772	1	0.05	0.5
4	IRGM	5	[-84, +687]	771	28	0.2	69	rs10067831	-41514	[-14, +362]	+238	41752	-2.246	34	49.28	0.999
						0.5	447	rs9324656	-55476	[-14, +362]	+269	55745	-2.604	15	3.36	0.8
						0.8	1047	rs10078968	+18188	[-84, +362]	+299	-17889	-2.685	1	0.1	0
						0.9	1459	rs10078968	+18188	[-84, +687]	+299	-17889	-2.679	1	0.07	0
7	MIR4520-2	17	[-1048, +47]	1095	50	0.2	77	rs9905053	-91311	[-752, +47]	-363	90948	2.062	68	88.31	0.999
						0.5	644	rs9905053	-91311	[-588, +47]	-374	90937	1.37	44	6.83	0
						0.8	1533	rs4796533	-2230	[-704, +47]	-483	1747	-2.33	1	0.07	0
						0.9	2146	rs4796533	-2230	[-659, +47]	-483	1747	-2.311	1	0.05	0
8	SDHAP3	5	[-15712, -14815]	897	76	0.2	76	rs73023414	-500387	[-15300, -14815]	-14852	485535	1.942	75	98.68	0.2
						0.5	726	rs37022	+164066	NA	-14815	-178881	-0.553	83	11.43	0
						0.8	1752	rs415144	-1657738	NA	-14989	1642749	-0.642	77	4.39	0
						0.9	2447	rs415144	-1657738	NA	-14983	1642755	-0.598	82	3.35	0
9	LCLAT1	2	[-526, +108]	634	46	0.2	64	rs74910977	+107411	[-526, -212]	-364	-107775	-1.822	22	34.38	0.8
						0.5	437	rs28378040	-3292	[-526, -212]	-366	2926	-1.735	2	0.46	0.5
						0.8	1120	rs72853154	-3593	[-526, -183]	-366	3227	-1.567	1	0.09	0.2
						0.9	1704	rs1615605	-12591	[-526, -171]	-364	12227	-1.712	1	0.06	0.5
10	LINC02610	2	[-1092, +337]	1429	82	0.2	79	rs72992741	+6677	[-73, +92]	-32	-6709	-1.776	55	69.62	0
						0.5	624	rs113905150	+13544	[-51, +13]	-32	-13576	-0.785	64	10.26	0
						0.8	1487	rs934945	-14735	NA	-32	14703	-0.611	49	3.3	0
						0.9	2049	rs934945	-14735	NA	-32	14703	-0.626	56	2.73	0
11	COMTD1	10	[-3090, +159]	3249	121	0.2	47	rs4746260	+153526	[-2431, -1983]	-2211	-155737	1.774	3	6.38	0.2
						0.5	247	rs72803500	+159512	[-2436, -1946]	-2211	-161723	1.796	3	1.21	0.2
						0.8	552	rs72803500	+159512	[-2431, -1983]	-2211	-161723	1.496	6	1.09	0.2
						0.9	797	rs79232706	+32996	[-2436, -1955]	-2211	-35207	1.711	1	0.13	0.2
13	SPRN	10	[+14, +138]	124	22	0.2	65	rs12243610	+612428	[+14, +138]	+14	-612414	1.654	64	98.46	0.5
						0.5	452	rs4625384	+1645040	[+30, +73]	+57	-1644983	-0.875	96	21.24	0.2
						0.8	1062	rs2035807	+1253406	[+14, +45]	+40	-1253366	0.819	84	7.91	0.2
						0.9	1475	rs869252	+1268249	[+32, +62]	+45	-1268204	0.839	96	6.51	0.2
14	PM20D1	1	[-373, +237]	610	32	0.2	68	rs56161922	-1990557	[-333, +237]	+237	1990794	-1.6	67	98.53	0.2
						0.5	425	rs10900525	+9634	[-333, +237]	+218	-9416	1.134	41	9.65	0
						0.8	988	rs823154	+56870	[-333, +237]	+237	-56633	-1.398	25	2.53	0
						0.9	1380	rs1772143	+19289	[-373, +237]	+237	-19052	-2.134	19	1.38	0
15	ADCY10P1	6	[-317, +142]	459	43	0.2	71	rs991762	-145363	[-317, +142]	-317	145046	-1.578	55	77.46	0.8
						0.5	460	rs62396316	+25355	[-317, +142]	-21	-25376	1.435	37	8.04	0.2
						0.8	1123	rs11963445	-55049	[-317, +142]	-21	55028	2.368	7	0.62	0
						0.9	1594	rs11963445	-55049	[-317, +142]	-265	54784	2.031	7	0.44	0
17	NLRP7	19	[-19310, -18581]	729	35	0.2	95	rs2914359	+1687189	[-19310, -18882]	-19310	-1706499	-1.523	89	93.68	0.8
						0.5	1103	rs34674770	+1343062	NA	-19310	-1362372	0.562	90	8.16	0
						0.8	2572	19:55486167	-27294	NA	-19310	7984	-0.485	95	3.69	0
						0.9	3489	rs6509911	-8204	NA	-19310	-11106	0.439	92	2.64	0
18	ZMAT5	22	[-1469, +22]	1491	67	0.2	58	rs5752590	+2199461	[-601, -373]	-496	-2199957	1.522	20	34.48	0
						0.5	405	rs132304	+606230	[-562, -396]	-481	-606711	-1.152	19	4.69	0
						0.8	1043	rs149125672	+607632	[-562, -423]	-481	-608113	-0.959	19	1.82	0
						0.9	1468	rs9613477	+2173079	[-514, -441]	-481	-2173560	-0.75	18	1.23	0
19	SPRNP1	10	[+578, +689]	111	21	0.2	65	rs4880435	+734133	[+578, +619]	+578	-733555	1.516	63	96.92	0.5
						0.5	427	rs11017777	+2419163	[+578, +586]	+578	-2418585	-1.007	73	17.1	0.2
						0.8	1012	rs11156513	+1919007	[+578, +591]	+578	-1918429	0.842	63	6.23	0.2
						0.9	1404	rs2492648	+1274053	[+578, +584]	+578	-1273475	-0.757	63	4.49	0.2
20	NBEAP1	15	[-85337, -84686]	651	44	0.2	41	rs17766894	+294357	[-84954, -84686]	-84861	-379218	1.501	35	85.37	0.8
						0.5	167	rs4966273	+41560	[-85008, -84686]	-84686	-126246	1.939	98	58.68	0.999
						0.8	315	15:21147536	-270939	[-85083, -84686]	-84686	186253	1.815	94	29.84	0.999
						0.9	412	15:21147536	-270939	[-84937, -84686]	-84855	186084	1.045	65	15.78	0.8

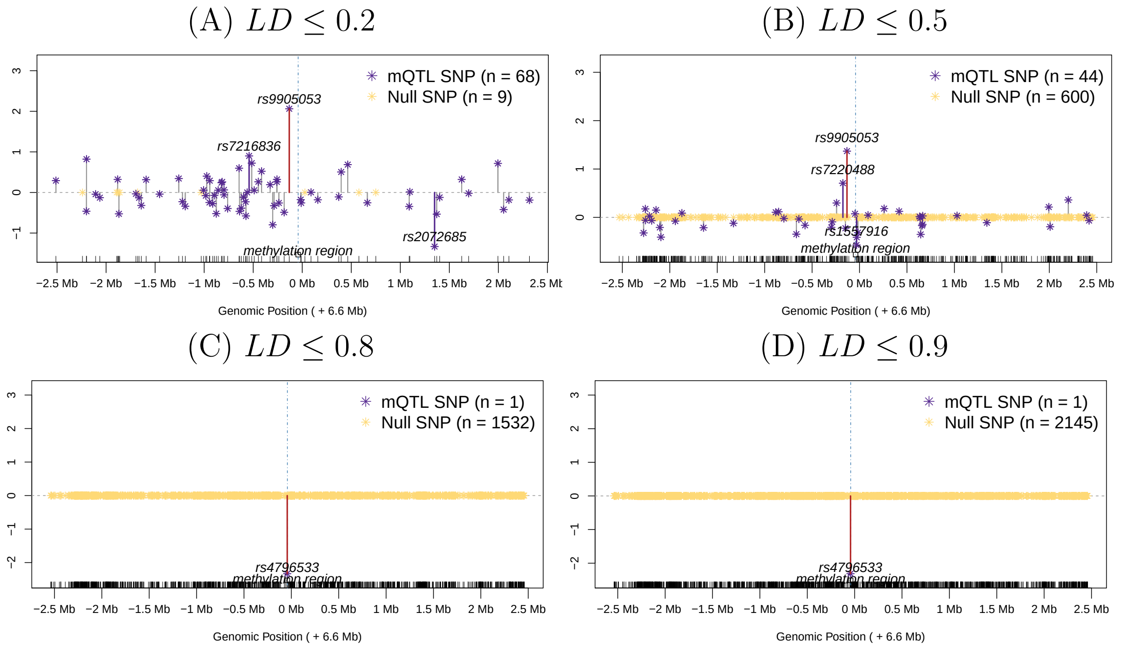

Figure S12. Peak estimated effects of each candidate SNP on the methylation region *MIR4520-2*, under different LD pruning thresholds.

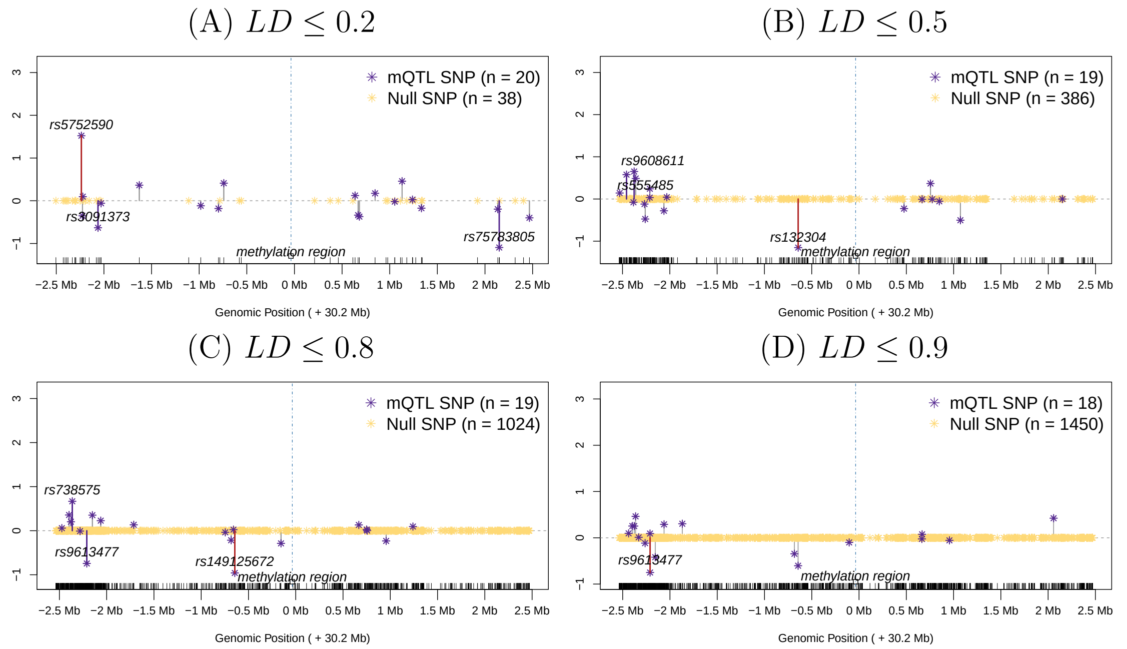

Figure S13. Peak estimated effects of each candidate SNP on the methylation region *ZMAT5*, under different LD pruning thresholds.

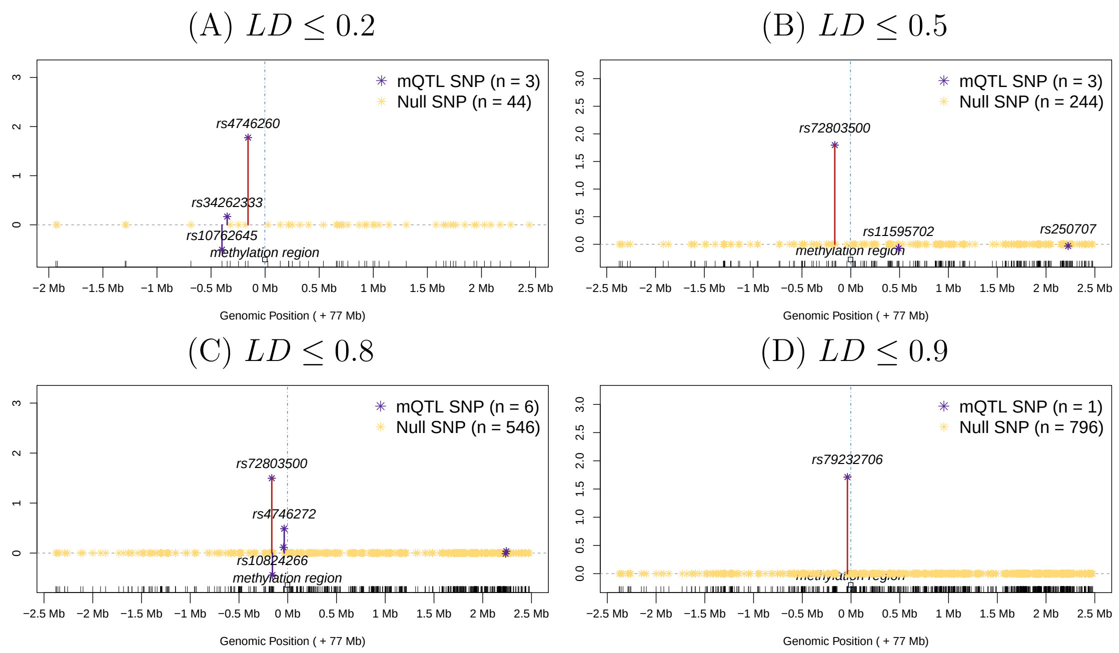

Figure S14. Peak estimated effects of each candidate SNP on the methylation region *COMTD1*, under different LD pruning thresholds.

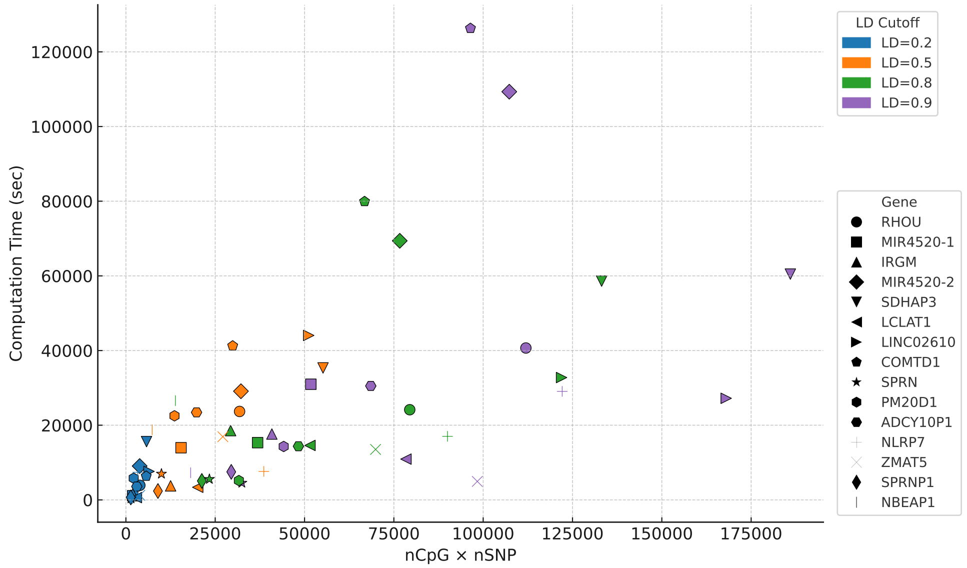

Figure S15. Computation time for sparseSOMNiBUS, using the adaptive SSP penalty with tuning parameter selected via 5-fold CV (1-SE rule), Each point represents a regional mQTL mapping run for a gene, with candidate SNPs specified under a given LD pruning threshold. The x-axis showing the product of the number of CpGs and LD-pruned SNPs in the region. Shapes indicate genes; colors denote LD thresholds (0.2, 0.5, 0.8). Computation time scales approximately linearly with the number of CpGs and SNPs included in the joint model. Exact timing results are shown in Table S21.

Gene	# of CpGs	LD cutoff	# of candidate SNPs	# of identified mQTLs	α	Computational time (in hours)
RHOU	63	0.2	62	62	0	1.084
		0.5	505	209	0	6.58
		0.8	1261	109	0	6.709
		0.9	1777	101	0	11.3
MIR4520-1	24	0.2	79	74	0.999	0.31
		0.5	643	39	0.8	3.881
		0.8	1536	1	0.5	4.258
		0.9	2157	1	0.5	8.605
IRGM	28	0.2	69	34	0.999	0.439
		0.5	447	15	0.8	1.044
		0.8	1047	1	0	5.154
		0.9	1459	1	0	4.9
MIR4520-2	50	0.2	77	68	0.999	2.518
		0.5	644	44	0	8.085
		0.8	1533	1	0	19.276
		0.9	2146	1	0	30.369
SDHAP3	76	0.2	76	75	0.2	4.337
		0.5	726	83	0	9.818
		0.8	1752	77	0	16.277
		0.9	2447	82	0	16.8
LCLAT1	46	0.2	64	22	0.8	0.168
		0.5	437	2	0.5	0.939
		0.8	1120	1	0.2	4.052
		0.9	1704	1	0.5	3.035
LINC02610	82	0.2	79	55	0	2.119
		0.5	624	64	0	12.237
		0.8	1487	49	0	9.102
		0.9	2049	56	0	7.558
COMTD1	121	0.2	47	3	0.2	1.767
		0.5	247	3	0.2	11.462
		0.8	552	6	0.2	22.202
		0.9	797	1	0.2	35.085
SPRN	22	0.2	65	64	0.5	0.096
		0.5	452	96	0.2	1.937
		0.8	1062	84	0.2	1.542
		0.9	1475	96	0.2	1.263
PM20D1	32	0.2	68	67	0.2	1.612
		0.5	425	41	0	6.255
		0.8	988	25	0	1.44
		0.9	1380	19	0	3.974
ADCY10P1	43	0.2	71	55	0.8	0.995
		0.5	460	37	0.2	6.511
		0.8	1123	7	0	3.99
		0.9	1594	7	0	8.478
NLRP7	35	0.2	95	89	0.8	2.056
		0.5	1103	90	0	2.119
		0.8	2572	95	0	4.73
		0.9	3489	92	0	8.074
ZMAT5	67	0.2	58	20	0	0.344
		0.5	405	19	0	4.702
		0.8	1043	19	0	3.756
		0.9	1468	18	0	1.375
SPRNP1	21	0.2	65	63	0.5	0.184
		0.5	427	73	0.2	0.659
		0.8	1012	63	0.2	1.411
		0.9	1404	63	0.2	2.086
NBEAP1	44	0.2	41	35	0.8	0.093
		0.5	167	98	0.999	5.216
		0.8	315	94	0.999	7.384
		0.9	412	65	0.8	2.016

Table S21. Computation time for `sparseSOMNiBUS` using the adaptive SSP penalty with tuning parameters selected via 5-fold cross-validation (1-SE rule). Each row represents a regional mQTL mapping run for a gene, with candidate SNPs specified under a given LD pruning threshold.

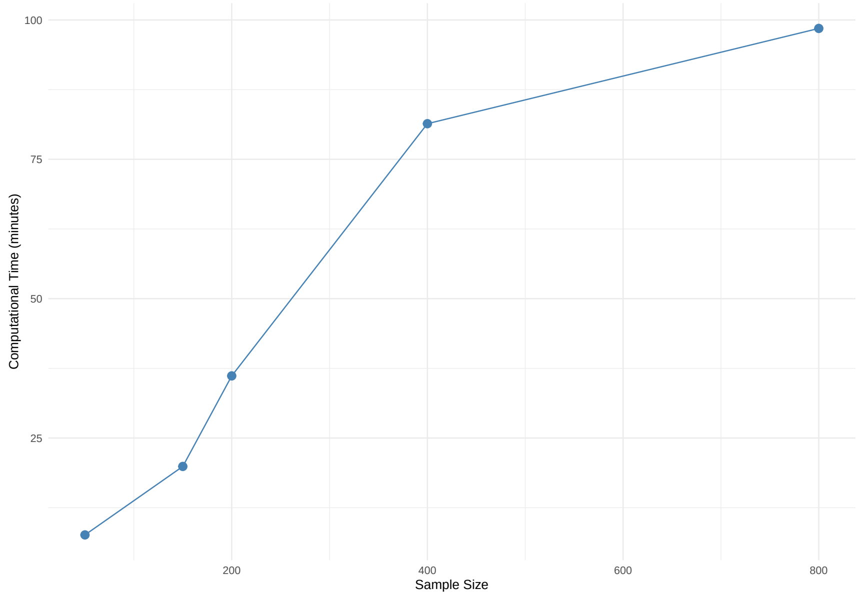

Figure S16. Computation time versus sample size under Simulation Example 1, with $P_{\text{true}} = 5$ mQTLs among a total of $P = 100$ candidate SNPs. Genotypes Z_p were simulated with a block-diagonal correlation matrix $\boldsymbol{\Sigma} \in \mathbb{R}^{P \times P}$, where each 20×20 block was defined as $\boldsymbol{\Sigma}^{\text{sub}} = (1-\rho)\boldsymbol{I} + \rho\boldsymbol{1}$ with correlation coefficient $\rho = 0.3$. The computation time increases approximately linearly with sample size.

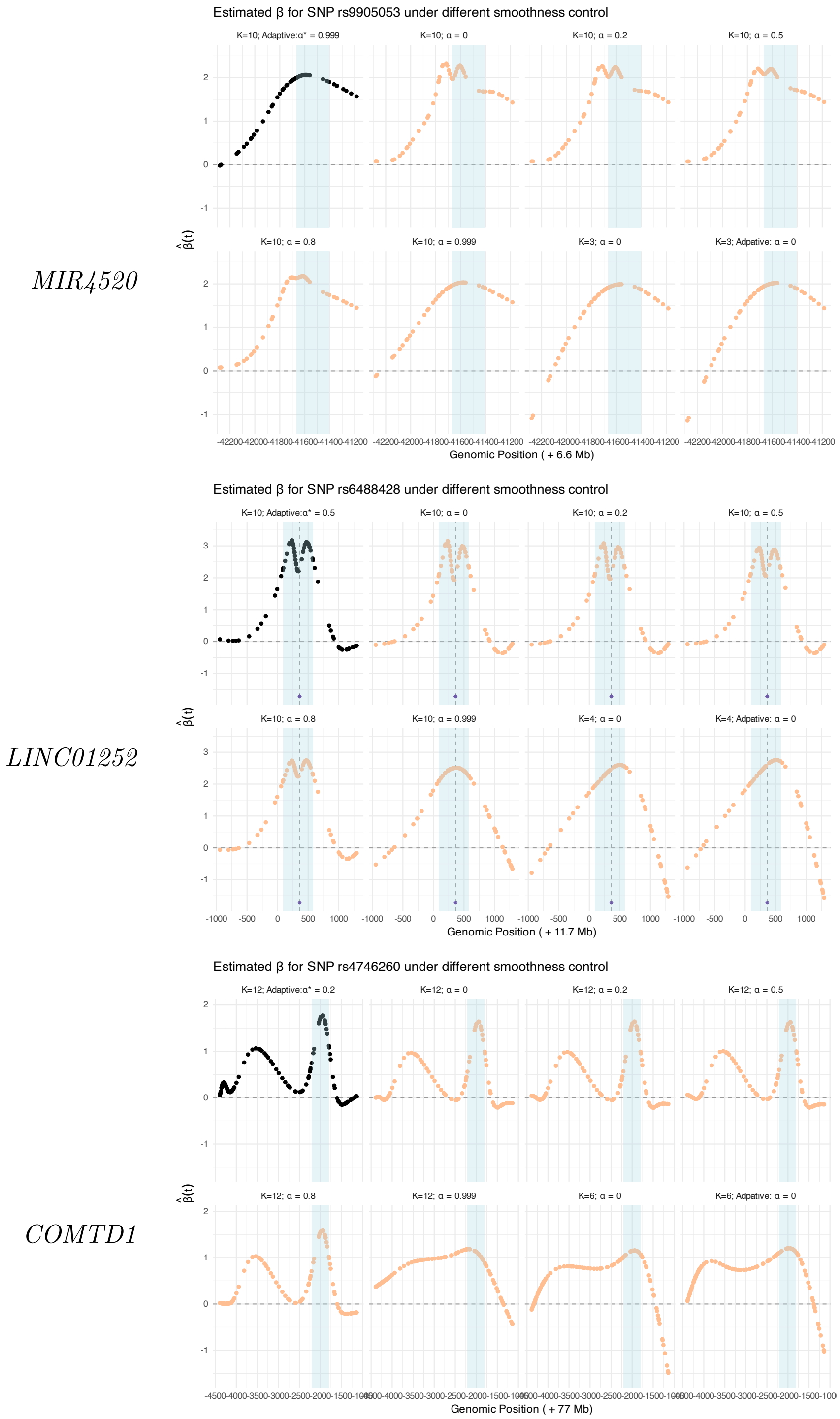

Figure S17. Estimated varying coefficient functions for the top associated SNP in three example genes (*MIR4520*, *LINC01252*, *COMTD1*) under different levels of smoothness control (α) and basis expansion (K). For each gene, the first panel shows the final model fit obtained using adaptive SSP with the optimal tuning parameters selected via cross-validation. The results illustrate how our method flexibly adapts to both localized and smooth signal patterns, highlighting the impact of smoothness control on signal detection.

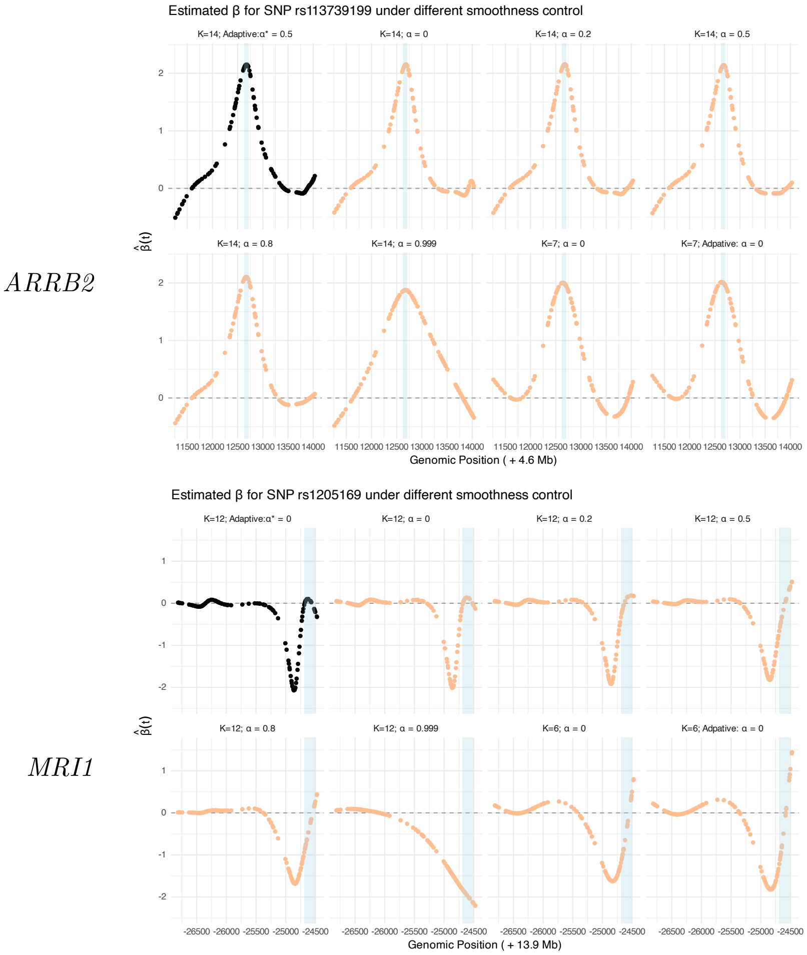

Figure S18. Estimated varying coefficient functions for the top associated SNP in two example genes (*ARRB2* and *MRI1*) under different levels of smoothness control (α) and basis expansion (K). For each gene, the first panel shows the final model fit obtained using adaptive SSP with the optimal tuning parameters selected via cross-validation. The results illustrate how our method flexibly adapts to both localized and smooth signal patterns, highlighting the impact of smoothness control on signal detection.

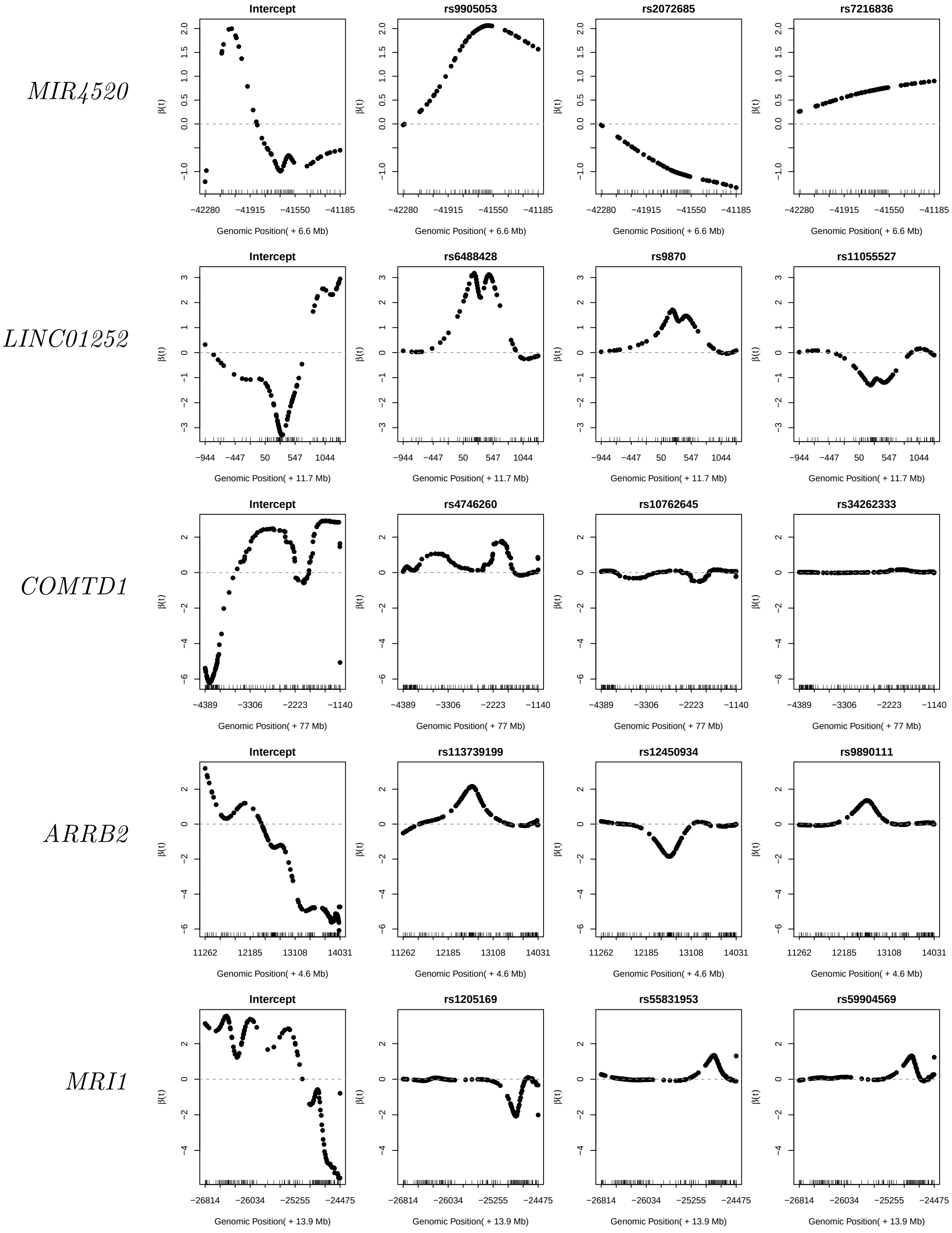

Figure S19. Estimated varying coefficient functions for the intercept and the top three SNPs in five example genes (*MIR4520*, *LINC01252*, *COMTD1*, *ARRB2* and*MRI1*). The results were obtained using adaptive SSP with with smoothness levels selected via cross-validation: $\alpha = 0.999$ for *MIR4520*, 0.5 for *LINC01252*, 0.2 for *COMTD1*), 0.5 for *ARRB2*, 0 for *MRI1*. The intercept function $\beta_0(t)$ is left unpenalized and often displays more wiggly patterns than the penalized genetic effect functions.

(A)

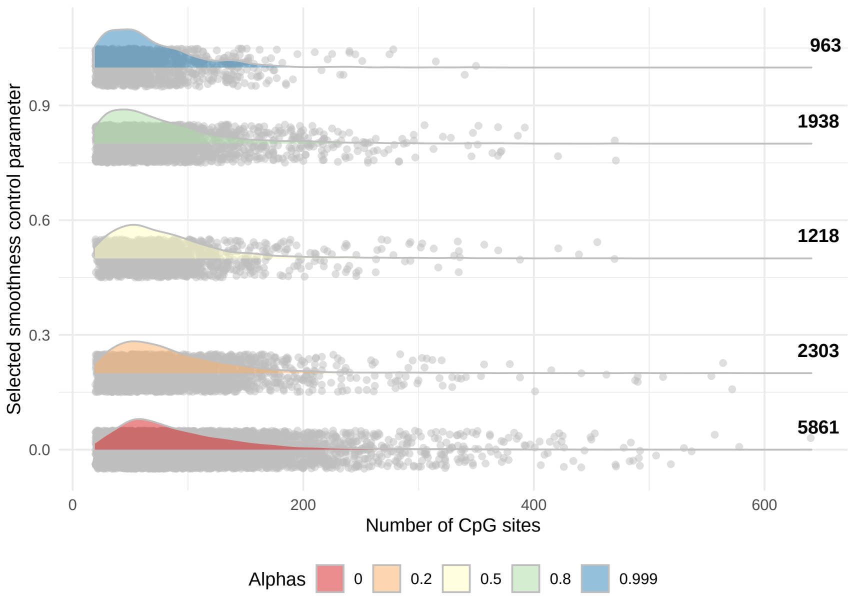

(B)

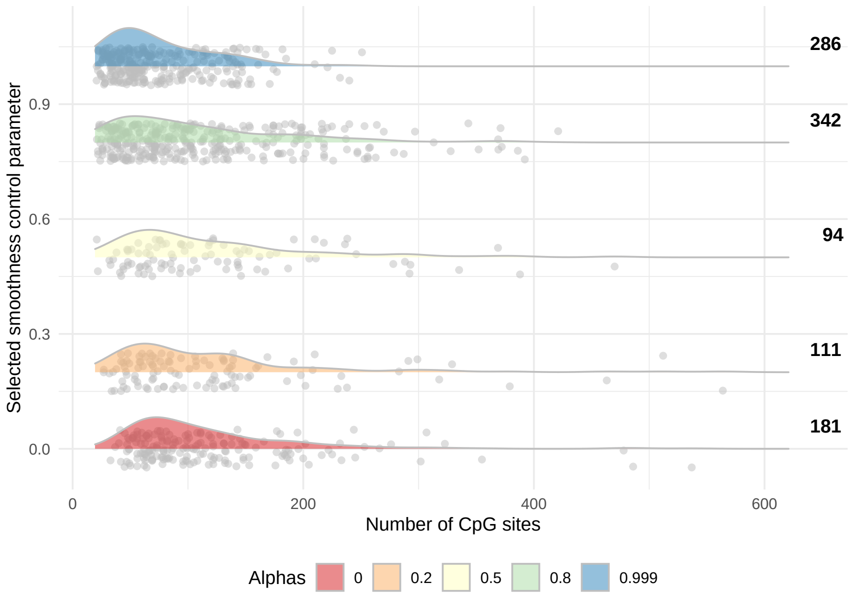

Figure S20. Distribution of selected smoothness control parameters (α) by number of CpG sites per region. (A) Results for all 12,283 methylation regions analyzed. $\alpha = 0$ was the most frequently selected overall (47.7%), particularly among regions with more CpGs; smaller regions showed greater variability, with both low and high α values observed. (B) Results for the 1,014 regions with detected mQTLs. In contrast to the full dataset, these regions exhibited a clear shift toward smoother fits, with a majority selecting moderate-to-high α values (33.7% at 0.8 and 28.2% at 0.999), and only 17.8% selecting $\alpha = 0$. These patterns suggest that smoother models better capture structured methylation variation in mQTL-active regions, while regions without such signals show more heterogeneous smoothness behavior.

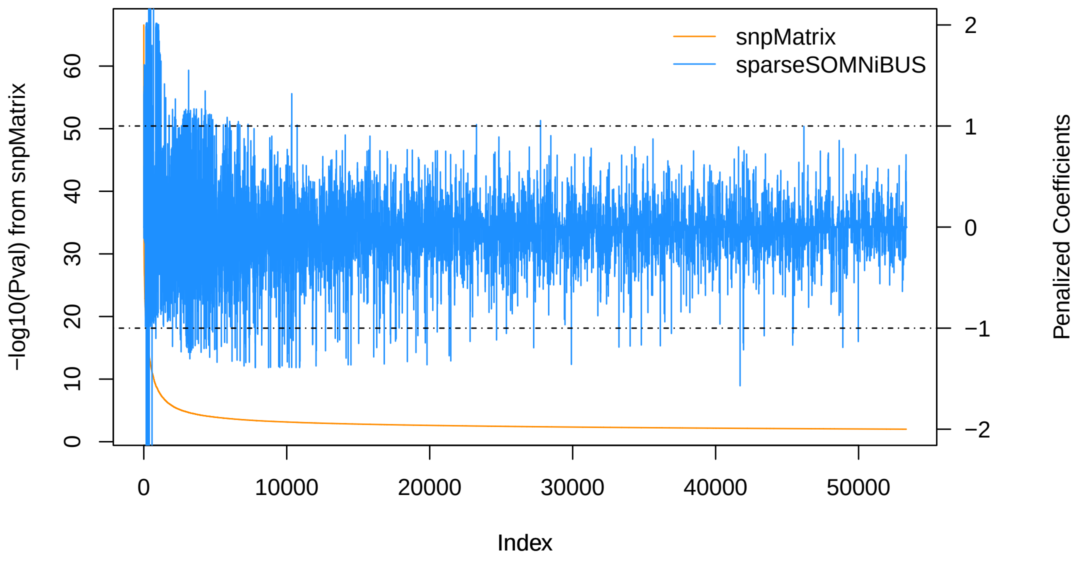

Figure S21. Comparison of the sparseSOMNiBUS coefficents and univariate p-values from snpMatrix. Each SNP-CpG pair is indexed on the x-axis in order of increasing univariate p-value (from *snpMatrix*). The orange curve (left y-axis) shows the $-\log_{10}$ univariate p-values, while the blue bars (right y-axis) show the corresponding penalized effect estimates from sparseSOMNiBUS. Horizontal dashed lines at ± 1 highlight the threshold for substantial estimated effects.

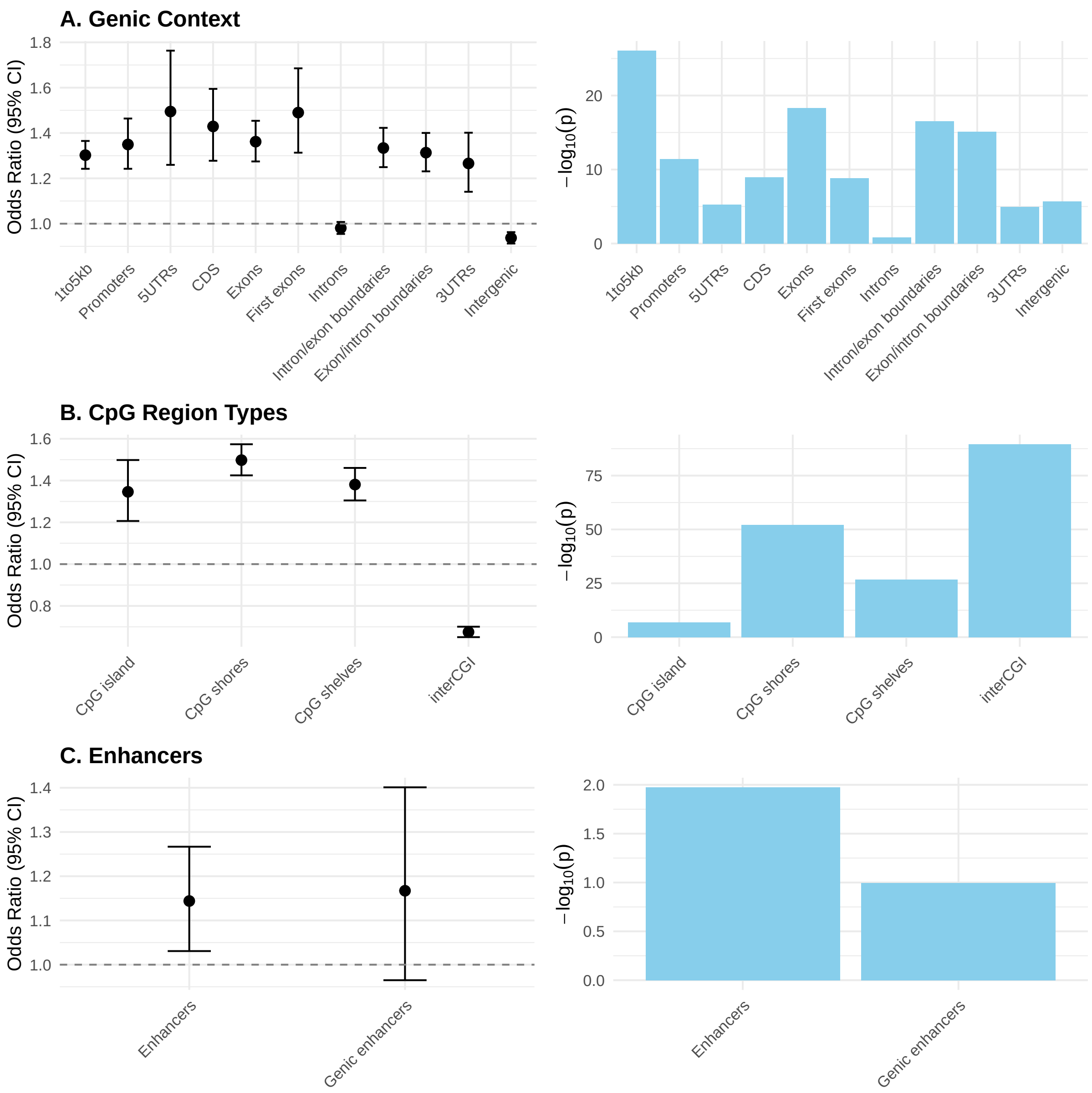

Figure S22. Functional enrichment analysis of mQTL SNPs across gene contexts (A), CpG region types (B), and enhancer annotations (C). In each panel, the left plot displays odds ratios with 95% confidence intervals, and the right barplot shows statistical significance ($-\log_{10}(p\text{-value})$). Enrichment was evaluated using Fishers exact test, comparing the overlap of mQTL SNPs with annotation categories against all LD-pruned SNPs tested ($r^2 < 0.2$). An odds ratio greater than 1 indicates over-representation of mQTLs in the corresponding annotation.

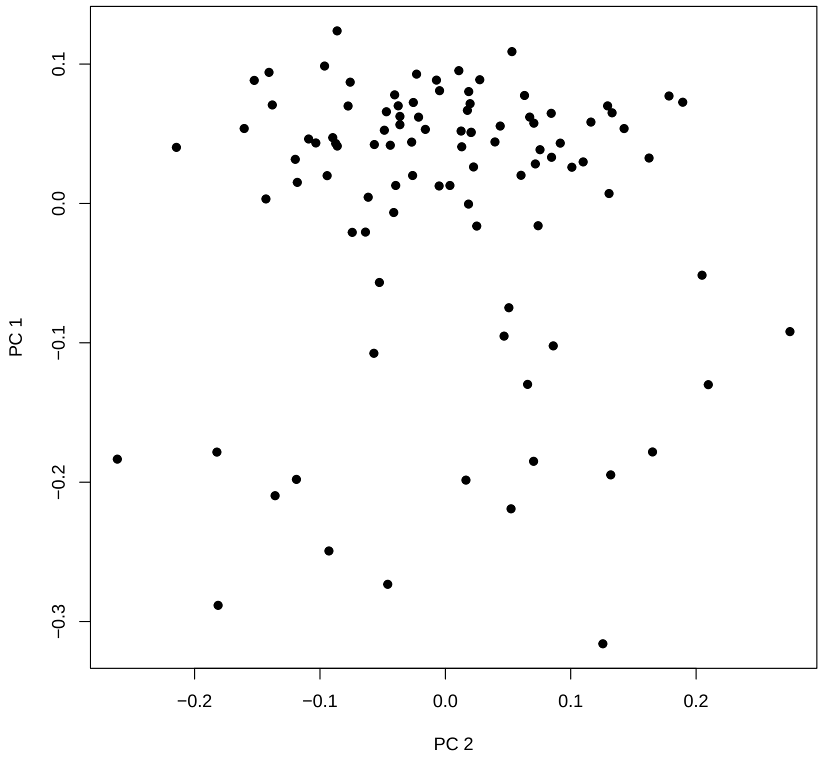

Figure S23. Scatterplot of the first two genotype principal components (PC1 vs. PC2) for all 98 individuals based on genome-wide SNP data (4,410,034 SNPs). The continuous spread of points and absence of discrete clusters suggest minimal population structure in the cohort.

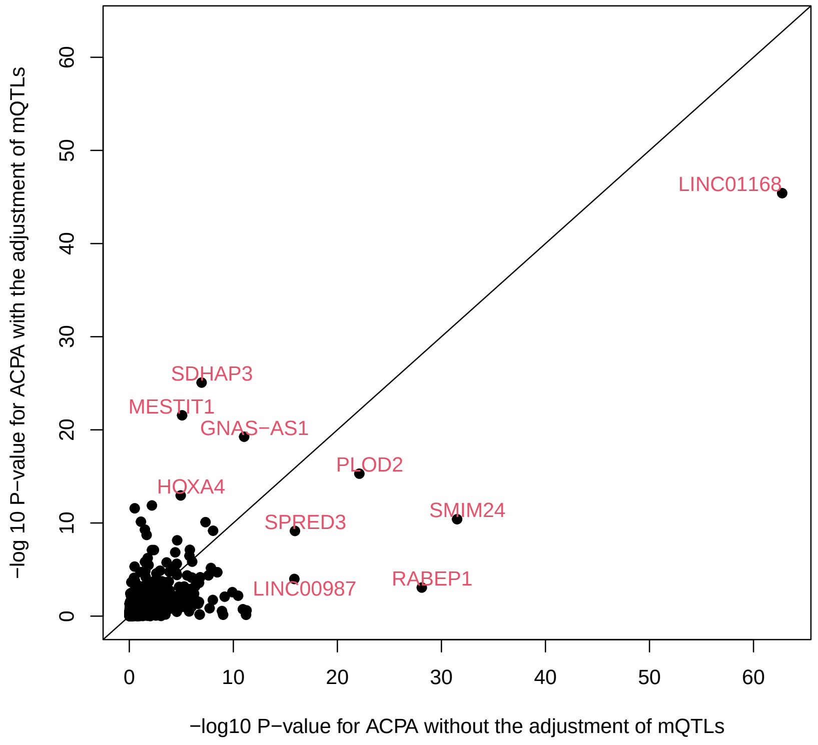

Figure S24. P-values (on the $-\log_{10}$ scale) for the effect of ACPA on regional methylation patterns in the 1014 genes with at least one mQTL. The horizontal axis displays the results from analysis without the adjustment of mQTLs, while the vertical axis displays the results from analysis with the adjustment of mQTLs.

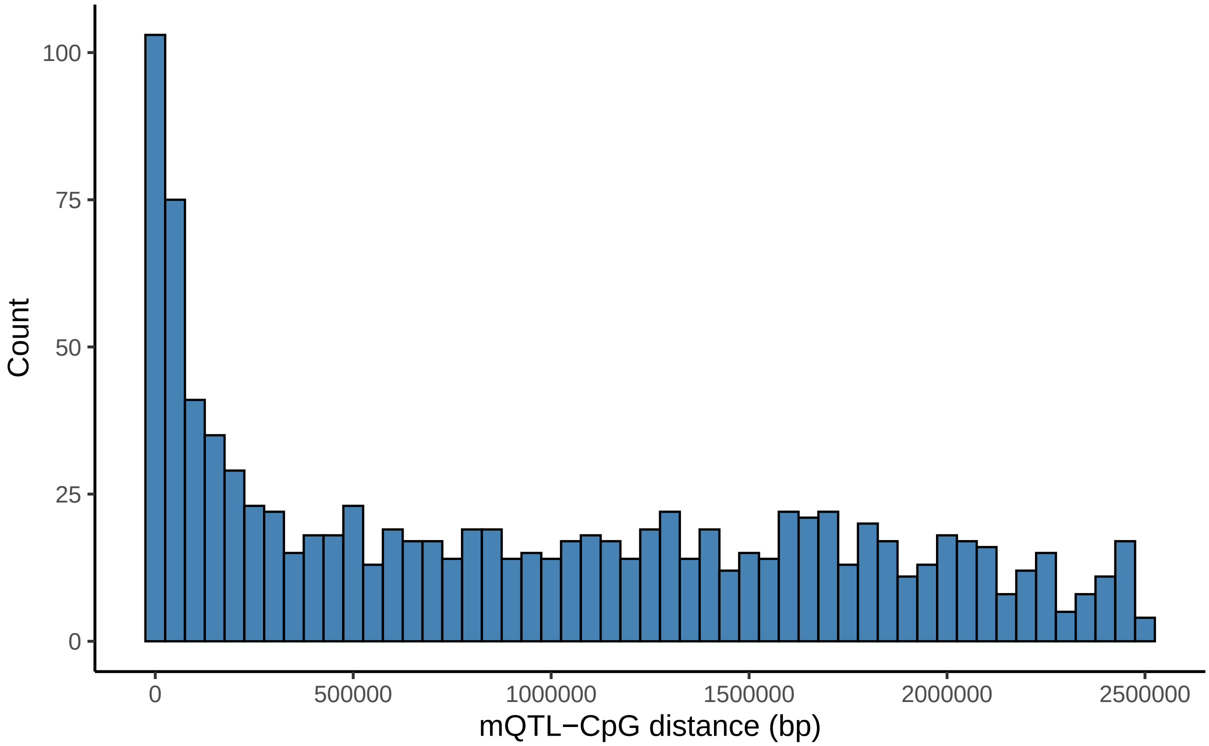

Figure S25. Histogram of top SNP-peak CpG distances (bp) across the 1,014 detected methylation regions. The distribution shows a sharp peak at 0-50 kb, followed by a relatively flat tail extending to 2.5 Mb, indicating enrichment of proximal associations together with appreciable distal effects.
